# Differential Effects of Prenatal Stress in 5-Htt Deficient Mice: Towards Molecular Mechanisms of Gene × Environment Interactions

**DOI:** 10.1371/journal.pone.0022715

**Published:** 2011-08-12

**Authors:** Daniel Van den Hove, Sissi Brigitte Jakob, Karla-Gerlinde Schraut, Gunter Kenis, Angelika Gertrud Schmitt, Susanne Kneitz, Claus-Jürgen Scholz, Valentina Wiescholleck, Gabriela Ortega, Jos Prickaerts, Harry Steinbusch, Klaus-Peter Lesch

**Affiliations:** 1 Institute of Molecular Psychiatry, Laboratory of Translational Neuroscience, Department of Psychiatry, Psychosomatics and Psychotherapy, University of Wuerzburg, Wuerzburg, Germany; 2 Department of Neuroscience, School for Mental Health and Neuroscience, Maastricht University, Maastricht, The Netherlands; 3 Laboratory of Translational Neuroscience, Department of Psychiatry, Psychosomatics and Psychotherapy, University of Wuerzburg, Wuerzburg, Germany; 4 Laboratory for Microarray Applications, Interdisciplinary Centre for Clinical Research, University of Wuerzburg, Wuerzburg, Germany; 5 Department of Neurophysiology, Ruhr University Bochum, Bochum, Germany; University of Queensland, Australia

## Abstract

Prenatal stress (PS) has been shown to influence the development of the fetal brain and to increase the risk for the development of psychiatric disorders in later life. Furthermore, the variation of human serotonin transporter (5-HTT, SLC6A4) gene was suggested to exert a modulating effect on the association between early life stress and the risk for depression. In the present study, we used a 5-Htt×PS paradigm to investigate whether the effects of PS are dependent on the 5-Htt genotype. For this purpose, the effects of PS on cognition, anxiety- and depression-related behavior were examined using a maternal restraint stress paradigm of PS in C57BL6 wild-type (WT) and heterozygous 5-Htt deficient (5-Htt +/−) mice. Additionally, in female offspring, a genome-wide hippocampal gene expression profiling was performed using the Affymetrix GeneChip® Mouse Genome 430 2.0 Array. 5-Htt +/− offspring showed enhanced memory performance and signs of reduced anxiety as compared to WT offspring. In contrast, exposure of 5-Htt +/− mice to PS was associated with increased depressive-like behavior, an effect that tended to be more pronounced in female offspring. Further, 5-Htt genotype, PS and their interaction differentially affected the expression of numerous genes and related pathways within the female hippocampus. Specifically, MAPK and neurotrophin signaling were regulated by both the 5-Htt +/− genotype and PS exposure, whereas cytokine and Wnt signaling were affected in a 5-Htt genotype×PS manner, indicating a gene×environment interaction at the molecular level. In conclusion, our data suggest that although the 5-Htt +/− genotype shows clear adaptive capacity, 5-Htt +/− mice –particularly females– at the same time appear to be more vulnerable to developmental stress exposure when compared to WT offspring. Moreover, hippocampal gene expression profiles suggest that distinct molecular mechanisms mediate the behavioral effects of the 5-Htt genotype, PS exposure, and their interaction.

## Introduction

Physical or emotional stress during pregnancy has been shown to influence the development of the fetal brain thereby increasing the risk for neuropsychiatric disorders in adulthood, particularly disorders of emotion regulation such as depression (see review by [1]). Likewise, prenatal stress (PS) exposure in rodents, particularly when exposure occurs during the last phase of pregnancy, is associated with a dysregulated hypothalamo–pituitary–adrenal (HPA) axis, concomitant with an increase in learning and memory deficits, as well as increased anxiety and depressive-like behavior in adulthood ([2]–[4]; see also reviews by [5], [6]). Nevertheless, the biological mechanisms by which PS exposure renders subjects vulnerable to the development of neuropsychiatric disorders are as yet not fully understood.

Moreover, variation in the serotonin transporter (5-HTT) gene were suggested to exert a modulating effect on the association between adverse experiences and the risk for depression [7], [8]. It has been argued that carriers of the short (s)-allele of the 5-HTT gene-linked polymorphic region (5-HTTLPR) are at greater risk for developing mood disorders associated with poor stress adaptation, while carriers of the long (l)-allele are relatively protected in this respect [9]. For example, a higher risk for depression was detected in maltreated children homozygous for the s-allele [10].

5-Htt deficient mice have been used to model the human allelic variation in 5-HTT function [11]. Generally, these mice show altered stress coping abilities, elevated anxiety- and depressive-like behaviors and memory deficits like a disability in extinction recall [12]–[18]. Interestingly, heterozygous 5-Htt deficient (5-Htt+/−) mice, which display a gene dose-dependent 50% reduction of the 5-Htt protein level, show an intermediate behavioral phenotype when compared to 5-Htt null mutant (5-Htt −/−) mice. This implies that 5-Htt +/− mice require an additional (stressful) trigger to develop signs of impaired emotional regulation. For example, Carola and colleagues revealed that maternal neglect early in life is required to increase anxiety-like behavior in 5-Htt +/− mice, an effect which was accompanied with an elevated *brain derived neurotrophic factor (Bdnf)* expression in the hippocampus [19]. As such behavioral responses to gene (G)×environment (E) interactions resemble findings of human studies [20], it was suggested that stress exposure of 5-Htt deficient rodents could serve as a model for the increased vulnerability to early life adversity in individuals with one or two 5-HTTLPR s-alleles [7]. Less is known, however, about the possible interaction between variation in the 5-HTT genotype and prenatal stress (PS) exposure.

The present study aimed to examine the effects of PS on cognition, anxiety- and depressive-like behavior using a maternal restraint stress paradigm of PS in C57BL6 wild-type (WT) and 5-Htt +/− mice. More knowledge on the molecular basis of such a G×E interaction might help to identify novel targets for the diagnosis and treatment of disorders of cognition and emotion regulation. For that purpose, we performed a genome-wide expression profiling on the hippocampus derived from the female offspring, which showed most pronounced behavioral changes mediated by variation in 5-Htt genotype, PS, and their interaction.

## Materials and Methods

### Ethics Statement

This study was approved by the Animal Ethics Board of Maastricht University, The Netherlands (Permit number: OE 2007-109). All efforts were made to minimize suffering.

### Animals and procedures

For this study, acclimatized male 5-Htt +/− and female 5-Htt +/− deficient mice ([B6.129(Cg)-Slc6a4tm1Kpl/J] [21]) were used for breeding. The animals were housed individually within a temperature-controlled environment (21±1°C) with 12 h light/12 h dark cycle (lights on from 7.00 h) and standard rodent chow and water available *ad libitum*. Pregnancy was determined by observation of vaginal plugs (embryonic day 0–E0). Prenatal maternal stress (n = 15) was performed daily during the last part of pregnancy (E13–E17) by restraining the dams in transparent 250 ml glass cylinders filled up to a height of 5 mm with water, whilst being exposed to bright light, 3 times daily (between 8.00 and 10.00 h, 12.00 and 14.00 h and 16.00 and 18.00 h), for 45 min per session (adapted from [22]).Control pregnant females (n = 14) were left undisturbed in their home cages. Maternal weight was measured at E0, E12 and E17. Litters were left undisturbed for 5 days after birth (P5) to prevent cannibalism. Only litters of 5 or more pups were included in the present study. Genotyping was performed by using polymerase chain reaction (PCR). DNA-fragments of either 225 bp refer to 5-Htt +/+, 272 bp to 5-Htt −/− or both to 5-Htt +/− mice. Offspring were individually housed in ventilated cages (TouchSLIMLine, Techniplast, Italy) after weaning (P25) under a reversed day-night cycle (12 h light/12 h dark cycle; lights on from 19.00 h). Pup mortality was monitored from P5 onwards. No more than two male and/or two female pups per litter were used to prevent litter effects [23]. When the offspring reached the age of 2 months (P60), behavioral experiments were started (n = 10–14/group). First, memory abilities were assessed using the object recognition task (ORT). Following this, anxiety- and depressive-like behavior were tested using the elevated zero maze (EZM) and forced swim task (FST), respectively. Tests were always performed in the dark phase (between 9.00 and 17.00 h for the ORT and between 9.00 and 13.00 h for the other tasks). In all experiments, males and females were tested separately. One week after behavioral tests, stress-induced plasma corticosterone (CORT) secretion was examined. One week later, the mice were sacrificed and brains removed. In addition, the adrenals were removed and weighted. Brains and blood samples were immediately placed on dry ice and stored at −80°C for future experiments.

#### Object recognition task (ORT)

Object recognition memory with mice was performed as described elsewhere [24]. The apparatus consisted of a circular arena, 43 cm in diameter. The test was performed with a constant illumination of approximately 20 lux. Two objects were placed symmetrically 5 cm away from the wall. We used four objects, (1) a cone made of brass (maximal diameter 6 cm and total height 3.8 cm), (2) a transparent glass bottle (diameter 2.7 cm, height 8.5 cm) filled with sand, (3) a metal cube (2.5 cm×5 cm×7.5 cm) with two holes (diameter 1.5 cm), and (4) an aluminium cube with a tapering top (4.5 cm×4.5 cm×8.5 cm). Each object was available in triplicate. In the first week, the animals were handled daily and were allowed to explore the arena, without any objects, twice for 5 min each day. Next, the mice were tested until they showed a good discrimination performance. A testing session comprised of two trials. The duration of each trial was 5 min. During the first trial (T1) the apparatus contained two identical objects. A mouse was always placed in the apparatus facing the wall at the middle of the front (transparent) segment. After the first exploration period, the mouse was put back in its home cage. Subsequently, after a predetermined delay interval (2, 3 or 4 h), the mouse was put back in the apparatus for the second trial (T2), but now with two dissimilar objects, a familiar one and a new one. The times spent exploring each object during T1 and T2 were recorded manually using a personal computer. Exploration was defined as follows: directing the nose to the object at a distance of no more than 2 cm and/or touching the object with the nose. Sitting on the object was not considered as exploratory behavior. In order to avoid the presence of olfactory cues the objects were always thoroughly cleaned with 70% ethanol before each trial. All combinations and locations of objects were used in a balanced manner to reduce potential biases due to preferences for particular locations or objects. Each delay interval was tested once in each animal. At least a two-day period was in between any delay test sessions with a particular animal. The testing order was determined randomly. The relative discrimination index (RDI) [time spent on new object in the second trial T2 - time spent on familiar object in T2]/total exploration time during T2) was determined for all mice.

#### Elevated zero maze (EZM)

The EZM is a task to measure anxiety-like behavior [25]. The test was carried out on a maze constructed of black plastic, transparent for infrared light. The circular runway was 50 cm in diameter, with a pathway width of 5 cm placed 10 cm above floor level. The maze was equally divided in 2 opposite open and 2 opposite closed parts enclosed by 50 cm high side walls. To prevent falls, a 5 mm high rim lined the open parts. A mouse was placed into the middle of one of the open parts, facing the outside of the maze and was allowed to explore the arena for a 5 min period. The distance travelled and % of time spent in the open parts of the maze was determined under low light conditions (20 lux) by use of an infrared video tracking system (Ethovision Pro, Noldus, The Netherlands; [26]). In order to avoid the presence of olfactory cues the arena was always thoroughly cleaned with 70% ethanol before each trial.

#### Forced swim test (FST)

The FST is commonly used to score behavioral despair in rodents [26], [27]. Animals were individually placed in a transparent perspex cylinder (40 cm tall; 19 cm in diameter; filled to a height of 15 cm with water of 31°C; [26]). Distance moved, as an indicator of mobility of the mice was scored in a 5 min session using a computerized system (Ethovision Pro, Noldus, The Netherlands).

### CORT response

When the offspring used in the behavioral testing reached an age of 3 months (P90) a blood sample was taken from the saphenous vein (basal CORT level). Subsequently, these mice were subjected to 20 min of restraint stress by a procedure identical to the PS procedure applied to the dams. Immediately following restraint stress, a second blood sample was taken (stress-induced CORT level). The mice were then returned to their home-cage for a 40 min recovery period, after which a third and final blood sample was taken (‘recovery’ CORT level). Blood collection, sample preparation and determination of plasma CORT levels was done as described in detail previously [28]. All blood samples were taken between 10:30–13:00 h.

### RNA isolation and microarray

In this study the left half of the hippocampus of females were used. The tissue was homogenized using 500 µl PegGOLD RNAPure (Peglab, Erlangen, Germany) and metal beads (3 min, 20 Hz in a Tissue Lyser (Qiagen, Hilden, Germany)). After that 100 µl chloroform was added and centrifuged (5 min, 4°C, 12.000× g). The water phase was then mixed with 250 µl ethanol and from that point on the protocol of the RNeasy Mini Kit (Qiagen, Hilden, Germany) was followed. RNA-quality was checked via Experion (Bio-Rad, Munich, Germany). Afterwards, the RNA was pooled, creating 3 pools per group. Prior to hybridization, RNA integrity and comparability were tested by a BioAnalyzer (Agilent Technologies, Palo Alto, CA). RNA integrity numbers (RIN) of all RNAs was between 8.3 and 8.6. cDNA synthesis, labelling and the actual microarray analysis was performed by the Interdisciplinary Centre for Clinical Research (IZKF) at the University of Wuerzburg. Generation of double-stranded cDNA, preparation and labelling of cRNA, hybridization to GeneChip® Mouse Genome 430 2.0 Arrays (Affymetrix, Santa Clara, CA) and washing was performed according to the standard Affymetrix protocol. The arrays were scanned using a GeneChip® Scanner 3000 (Affymetrix Santa Clara, CA). Data analysis was done using different R packages from the Bioconductor project (www.bioconductor.org). Probe sets were summarized using the PLIER algorithm. Resulting signal intensities (signal intensity from a specific probeset is referred to as the expression of the associated gene from here onwards) were normalized by variance stabilization normalization (VSN) [29]. Quality and comparability of all data sets were tested by density plot, RNA degradation plot and correspondence analysis. All data is MIAME compliant and the raw data has been deposited in the Gene Expression Omnibus (GEO) (accession number: GSE26025).

### Enriched pathway analysis

The Database for Annotation, Visualization and Integrated Discovery (DAVID) 2007 Functional Annotation Clustering was used to search the database of the Kyoto Encyclopedia of Genes and Genomes (KEGG) [30], [31] in order to identify significantly over-represented pathways in the subset of differentially expressed genes. More specifically, the latter is a curated pathway database comprising biological signaling pathways that are based on current knowledge of molecular interactions involved in various cellular processes. Settings used were: Count (2); EASE (0.1); P<0.05.

### Quantitative PCR

The validity of the microarray results was subsequently tested via quantitative real-time PCR (qRT-PCR) employing the Bio-Rad CFX384 Real-Time PCR Detection System (in technical triplicates). For validation we selected 8 genes which showed a fold change (FC)>1.5 (or 2.5) in the microarray (see below). The same RNA as for the microarray was utilized for cDNA synthesis which was performed by the use of the iScript™ kit (Bio-Rad, Munich, Germany) according to the manufacturer's instructions. Mean efficiencies were calculated by LinReg [32]. Reference genes for normalization were selected from the microarray and tested for stability using geNorm [33]. *CCCTC-binding factor (Ctcf), guanosine diphosphate (GDP) dissociation inhibitor 2 (Gdi2)* and *gap junction protein*, *alpha 1 (Gja1)* were used for normalization. Relative expression data were calculated with the normalization factors obtained from geNorm and the mean efficiencies from LinReg.

### Statistical analysis

Percentage of maternal weight increase over the last week of gestation was compared using a one-way ANOVA (condition). EZM and FST data were explored by three-way ANOVAs (genotype×condition×sex). Data on the ORT were analyzed using a repeated measures ANOVA, as well as by a separate analysis at the distinct time-points. Furthermore, for the ORT the RDI from every group was compared to an RDI of 0 (no discrimination) as described previously [24]. CORT data were ln-transformed prior to ANOVA and were analyzed using a repeated measures ANOVA, as well as by a separate analysis at the distinct time-points. Overall interaction effects were examined in more detail using Least Significant Difference (LSD) tests. In the absence of an interaction, main effects of genotype and condition were analyzed by an additional stratified analysis – i.e. stratified per genotype and sex in case of a condition effect, in order to test whether overall effects were specific to, or more pronounced in, a particular genotype or sex. This was expected, since the 5-HTT genotype is known to have specific effects on various behavioral phenotypes and may selectively affect the interaction with stressful life events (see e.g. [34]), whereas, in addition, PS is known for its sex-specific effects on offspring outcome (e.g. [22]). The failure to detect significant interactions using the three-way ANOVA approach may be explained by the relative conservative nature of F-tests in general, in combination with the intricate logistical experimental design (with its associated breeding restrictions), resulting in a relative lack of statistical power. Perinatal and post-weaning mortality were examined using a one-sided Fisher's exact test. Statistical analysis to select differentially expressed genes was performed using the Linear Models for Microarray Analysis (LIMMA) package [35], [36]. LIMMA is a library for the analysis of gene expression microarray data, especially concerning the use of linear models for analyzing designed experiments and the assessment of differential gene expression profiles. As an output a table of the top-ranked genes from the linear model fit including a gene list, ratio on the log2 scale, average gene intensities, moderated t-statistic, adjusted P-value (false discovery rate) and log odds were created. By using LIMMA we calculated the following differences: between 5-Htt +/− (HET) and WT mice [(HETPS+HETC)-(WTPS+WTC)] (G effect), between PS and control (C) animals [(WTPS+HETPS)-(WTC+HETC)] (E effect) and the interaction of G and E [(HETPS-HETC)-(WTPS-WTC)] (G×E effects). Genes were identified as differentially expressed if they showed a nominal P-value less than 0.01. There was no “cut off” for the linear FC concerning the microarray data (FC of 1 indicates no change, while a FC of 2 equals a double amount of cRNA). Results of the microarray were validated by means of qRT-PCR. For the G and E effects we considered only genes with a FC>1.5 to achieve reliable validation. We further restricted the selection by only choosing genes with an annotation grade (see affymetrix.com) of A or B into account. For an exact validation, we used only those genes for which we were able to amplify the same sequence as recognized by the microarray. These criteria encouraged us to pick *FBJ osteosarcoma oncogene* (*Fos*) and *paired-like homeobox 2a* (*Phox2a*) out of the 15 genes altered >1.5 fold by PS. Of the 29 genes altered >1.5 fold by the 5-Htt+/− genotype we selected *XIAP associated factor 1* (*Xaf1*), *zinc finger*, *ZZ-type with EF hand domain 1* (*Zzef1*), *protein phosphatase 1, regulatory (inhibitor*) *subunit 1B* (*Ppp1r1b*), *Kv channel-interacting protein 2* (*Kcnip2*) and *myelin basic protein* (*Mbp*). In addition, we validated the G×E effect of the *thyrotropin releasing hormone receptor* (*Trhr*), as the expression of this gene between WTC and WTPS differed 2.5 fold. Gene expression data using qRT-PCR were analysed by two-way ANOVA (genotype×condition). The level of statistical significance was assumed to exist at P<0.05 in all tests. Except for microarray data analysis, all statistical analyses were performed using the SPSS 15.0 software package.

## Results

### Dam weights during gestation and litter sizes

Dam weight increase during pregnancy is depicted in Table 1. No differences in weight increase during the first two weeks of gestation between stressed versus unstressed dams were observed. Over the last week of pregnancy stressed dams gained significantly less weight when compared to control animals (F_1,25_ = 25.024; P<0.001). Further, no differences were found in the litter sizes from stressed and control dams.

**Table 1:** Dam weight during pregnancy and litter size.

Condition	% Weight increase	% Weight increase	Litter size
	(E0–E12)	(E12–E17)	
**C**	33.78±2.41	30.04±1.16	7.08±0.72
**PS**	33.44±2.13	17.52±1.94***	7.25±0.66

During the last part of pregnancy (E12–E17), stressed (PS) dams gained significantly less weight compared to control (C) animals (***P<0.001). Data represent means ± S.E.M. N = 12–15 litters/condition.

### Pre- and postweaning mortality

Preweaning mortality was not different between the various groups (data not shown). A significantly higher postweaning mortality (P = 0.04) was observed in PS offspring (7 out of 51 [13.7%] in PS animals vs. 1 out of 44 [2.3%] in controls; data not shown). Genotype had no effect on postweaning mortality.

### Offspring cognition, anxiety, and depressive-like behavior

Memory performance in the object recognition task is depicted in Table 2. With increasing interval duration, animals showed lower recognition scores (F_6,58_ = 21.993; P<0.001). Further, overall, an interval×genotype (F_6,58_ = 24.851; P = 0.027) and a condition×sex interaction (F_7,58_ = 4.273; P = 0.043) were observed, the latter of which indicated that PS particularly impaired memory performance in female offspring. When looking at the individual intervals, at the 2-hour interval, all animals were able to distinguish the old from the new object. Further, a significant condition×sex interaction was seen (F_7,60_ = 6.518; P = 0.013). Specifically, PS was associated with impaired memory performance in female offspring as post-hoc analysis was showing a significant decrease in memory performance in PS versus control females (P = 0.049). At the 3-hour interval, among males, only WT controls were able to remember the old object. Further, all 5-Htt +/− groups still showed intact memory performance. In line with this, a significant overall effect of genotype was observed at this interval (F_7,62_ = 4.501; P = 0.038), with 5-Htt +/− mice showing improved memory function as compared to WT animals. At the 4-hour interval, all groups showed impaired memory performance. Of note, no differences in exploration times between groups were observed at any interval.

**Table 2:** Memory performance as assessed in the Object Recognition Test (ORT).

Group			2 h	3 h	4 h	Exploration Time
**WT**	**M**	**C**	**0.286±0.092**	**0.296±0.066**	0.081±0.074	18,161±0,838
		**PS**	**0.452±0.080**	0.195±0.103	0.148±0.066	17,825±0,730
	**F**	**C**	**0.358±0.078**	0.096±0.065	0.225±0.086	18,373±0,831
		**PS**	**0.319±0.082**	0.116±0.102	−0.132±0.123	17,976±0,951
**5Htt +/−**	**M**	**C**	**0.355±0.067**	**0.248±0.070**	0.054±0.085	18,578±0,669
		**PS**	**0.436±0.075**	**0.393±0.056**	−0.014±0.066	17,910±0,742
	**F**	**C**	**0.509±0.087**	**0.281±0.062**	0.001±0.099	18,131±1,400
		**PS**	**0.217±0.071**	**0.266±0.098**	0.056±0.093	17,657±0,758

**Bold data indicate intact memory performance, i.e., when animals were able to distinguish the old from the new object. At the 2-hour interval, a significant condition×sex interaction was observed (P = 0.013). At the 3-hour interval, a significant overall effect of 5-Htt genotype was observed (P = 0.038). See** **results** **section for more details. Data in the first three columns represent mean relative discrimination index (RDI) ± S.E.M. The last column shows the average exploration times (the average time spent exploring each object during T1 and T2, averaged over the 3 intervals), which did not differ between groups. Abbreviations: WT, wild-type; M, males; F, females; C, control offspring; PS, prenatally stressed offspring. N = 7–10 mice/group.**

**Figure 1:**
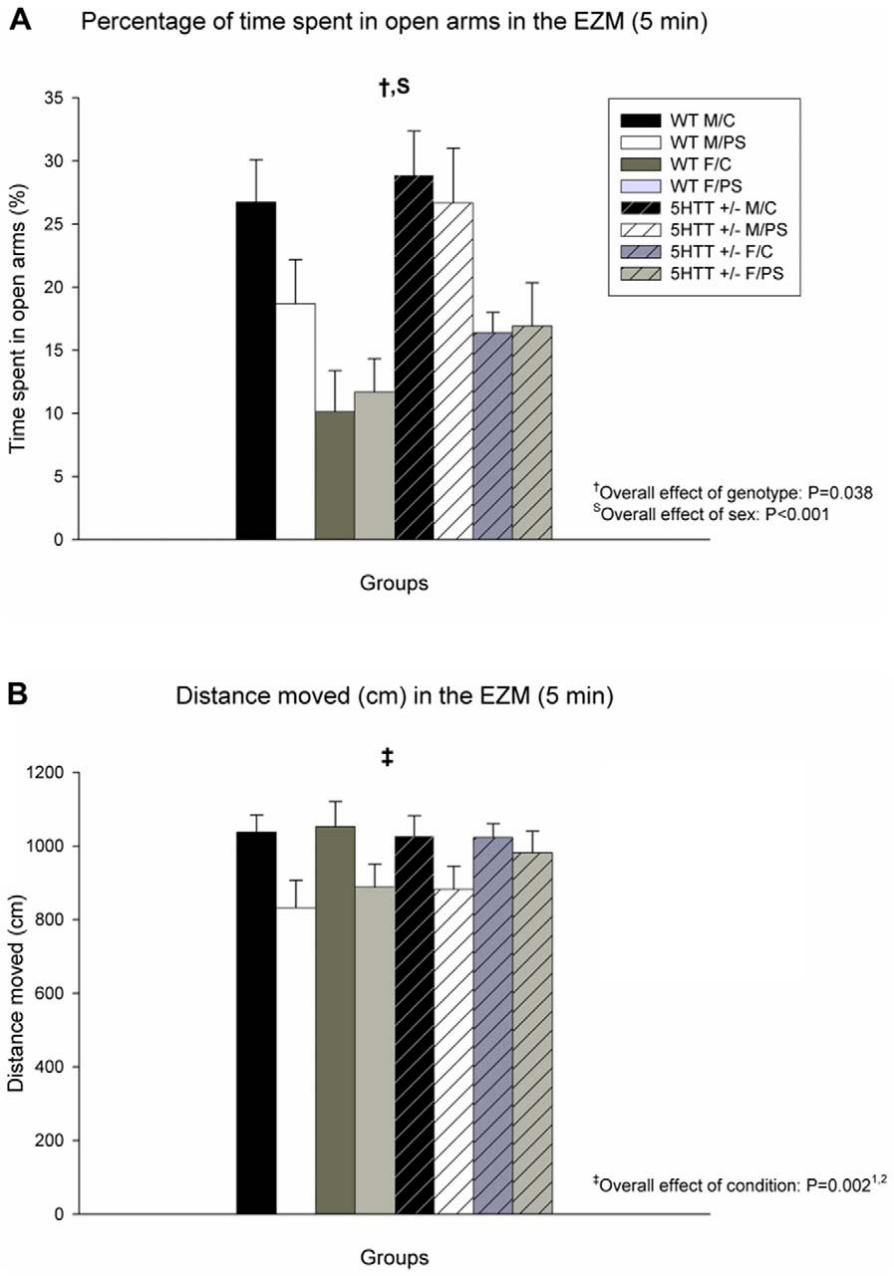
Performance in the Elevated Zero Maze (EZM). A) Time spent in the open arms of the EZM was significantly increased in 5-Htt +/− versus wild-type (WT) animals (P = 0.038). In addition, females (F) spent less time in the open arms of the EZM when compared to male (M) offspring (P<0.001). B) Distance covered within the EZM was decreased by prenatal stress (PS; P = 0.002). Data represent mean+S.E.M. Abbreviation: C, control offspring. N = 7–10 mice/group. ^1^Only significant in WT offspring when stratified for genotype; ^2^Only significant in male offspring when stratified for sex (see results section for more details).

The effects of PS on anxiety-like behavior in the EZM are shown in Figure 1. Time spent in the open arms of the EZM was significantly increased in 5-Htt +/− versus WT animals (F_7,64_ = 4.466; P = 0.038), indicating lower levels of anxiety in 5-Htt +/− offspring. In addition, females spent less time in the open arms of the EZM (F_7,64_ = 20.091; P<0.001), indicating higher levels of anxiety in this sex. Distance covered within the EZM was decreased by PS (F_7,64_ = 10.314; P = 0.002). When stratifying the analysis per genotype, the observed PS effect was only significant in WT, but not in 5-Htt +/− offspring (F_3,29_ = 8.343; P = 0.007, versus F_3,30_ = 2.493; P = 0.123, respectively). Similarly, when stratifying for sex, the PS effect was only significant for males and not for females (F_3,34_ = 7.199; P = 0.011, versus F_3,30_ = 3.527; P = 0.070, respectively).

**Figure 2:**
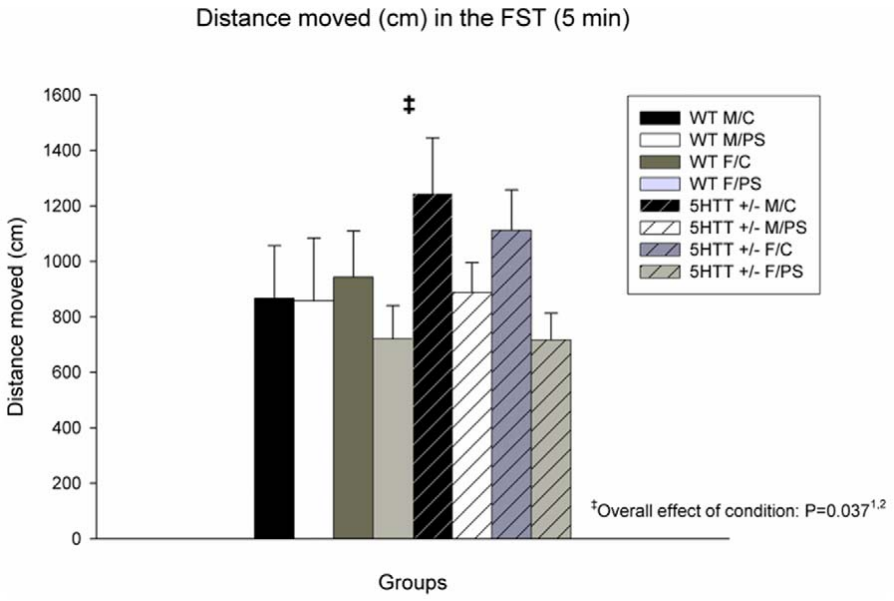
Performance in the Forced Swim Test (FST). Distance swum in the FST (5-minute period) was significantly decreased in prenatally stressed (PS) versus control (C) animals (P = 0.037). Data represent mean+S.E.M. Abbreviation: WT, wild-type. N = 7–10 mice/group. ^1^Only significant in 5-Htt +/− offspring when stratified for genotype; ^2^Only significant in female offspring when stratified for sex (see results section for more details).

Depressive-like behavior in the FST is depicted in Figure 2. Overall, a significant effect of condition was observed, indicating that PS animals exhibit more depressive-like behavior (F_7,67_ = 4.544; P = 0.037). When stratifying the FST analysis per genotype, the observed PS effect was only significant in 5-Htt +/−, but not in WT offspring (F_3,36_ = 6.869; P = 0.013, versus F_3,31_ = 0.395; P = 0.534, respectively). Similarly, when stratifying for sex, the PS effect was only significant for females (F_3,35_ = 0.980; P = 0.329 versus F_3,32_ = 5.494; P = 0.025, for males and females, respectively). Of note, these observations also suggests that the effect in the FST was independent from the lower mobility as observed in the EZM, the latter of which was primarily seen in WT male offspring (Figure 1B).

### Stress-induced plasma corticosterone (CORT) secretion

**Figure 3:**
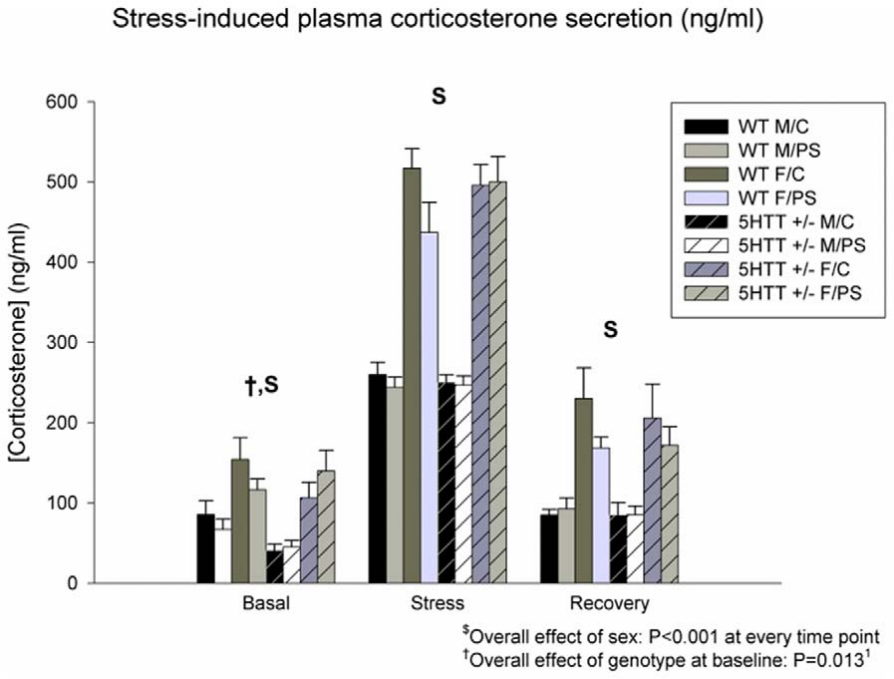
Stress-induced plasma corticosterone (CORT) secretion. At all time points, female (F) offspring had higher CORT levels when compared to male (M) offspring (overall sex effect; P<0.001 in all cases). At baseline, a significant genotype effect was observed (P = 0.013). Abbreviations: WT, wild-type; C, control offspring. Data represent mean+S.E.M. N = 7–10 mice/group. ^1^Only significant in male offspring when stratified for sex (see results section for more details).

Data on stress-induced plasma CORT secretion of the offspring are shown in Figure 3. Overall, a significant effect of time was seen (F_6,63_ = 263.413; P<0.001). Furthermore, a time×genotype interaction was observed (F_6,63_ = 3.594; P = 0.030). In addition, over all 3 time points, significant effects of genotype and sex (F_7,64_ = 4.000; P = 0.050 and F_7,64_ = 92.908; P<0.001, respectively) were seen, indicating lower CORT levels in 5-Htt +/− versus WT mice and higher levels in female versus male offspring, respectively. When looking at the individual time points, female offspring had higher CORT levels when compared to male offspring at all time points (overall sex effect; F>37.187; P<0.001 in all cases). Further, at baseline, an overall genotype effect (F_7,65_ = 6.476; P = 0.013) was observed, indicating that basal CORT levels were lower in 5-Htt +/− as compared to WT mice, an effect that tended to be more profound in male offspring (when stratified for sex; F_3,33_ = 6.678; P = 0.014, versus F_3,32_ = 1.444; P = 0.238, in males versus females; see Figure 3).

### Adrenal weight

**Figure 4:**
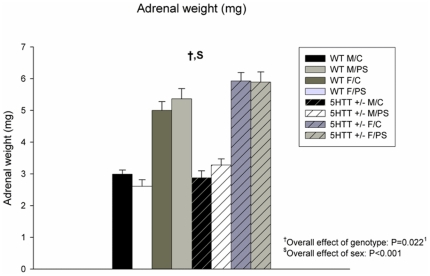
Adrenal weight. Adrenal weight was higher in female (F) versus male (M) offspring (P<0.001). Adrenal weight was increased in 5-Htt +/− offspring when compared to wild-type (WT) animals (P = 0.022). Data represent mean+S.E.M. Abbreviations: C, control offspring; PS, prenatally stressed offspring. N = 7–10 mice/group. ^1^Only significant in female offspring when stratified for sex (see results section for more details).

Adrenal weight was higher in female versus male offspring (F_7,56_ = 166.817; P<0.001; Figure 4). In addition, overall, adrenal weight was higher in 5-Htt +/− offspring when compared to WT animals (F_7,56_ = 5.524; P = 0.022), an effect that seemed to be particularly present in females (F_3,30_ = 5.902; P = 0.021, versus F_3,26_ = 0.517; P = 0.478, for females and males, respectively; see Figure 4 for more details).

### Gene expression analysis

#### Microarray analysis

**Figure 5:**
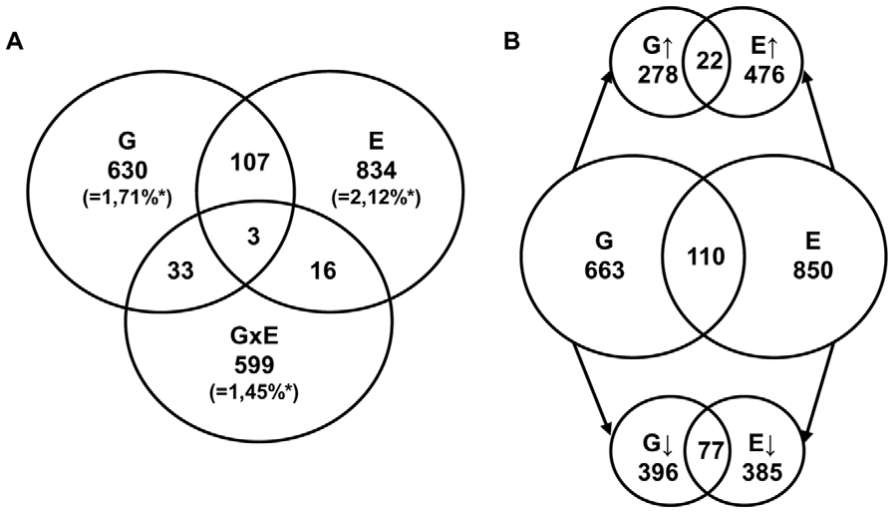
Gene expression analysis. A) Venn diagram illustrating the number of genes altered by the genotype (G; i.e. 5-Htt +/− versus wild-type, 773 genes), the environment (E; i.e. prenatal stress versus control, 960 genes), both (110 genes), or in an interactive manner (G×E; i.e. indicating those genes of which the effect of the environment depends upon the genotype, 651 genes). B) Venn diagram illustrating the number of genes regulated by G, E or both, including the corresponding direction in which the 5-Htt +/− genotype and PS regulated their expression. Eleven genes were regulated in an opposite direction by G and E (not shown); *Percentage of probesets examined.

To further investigate the molecular mechanisms underlying the behavioral observations in female offspring –which showed most pronounced behavioral changes mediated by variation in 5-Htt genotype, PS, and their interaction–, we conducted a microarray-based expression profiling on the female hippocampus, a brain region participating in learning and memory as well as in emotion regulation [37]. For microarray analysis we focused on three different comparisons. We assessed the changes between 5-Htt +/− and WT mice (G effect), in the differences between PS and control mice (E effect), and, moreover, in the interaction between G and E (G×E effects), i.e. indicating those genes of which the effect of PS exposure depended upon the 5-Htt genotype. In brief, the 5-Htt +/− genotype and PS exposure altered the expression of 773 and 960 genes, respectively (Figure 5; also see Text S1 and S2 for a complete overview of all genes significantly affected by G and E, respectively). Furthermore, 651 genes were affected in a G×E manner (Text S3; also see Figure 5). In addition, G and E showed overlap in the expression of 110 genes. Of those, 22 genes were upregulated, and 77 were downregulated by both. 11 genes were affected by G and E in an opposite direction (see Figure 5) whereas the expression of 3 genes was altered by G, E and in a G×E manner. To functionally categorize the differentially expressed genes we performed a pathway analysis using DAVID. We found 10 KEGG pathways affected by G, 9 by E and 10 by G×E (Table 3).

**Table 3:** Significant KEGG pathways^1^ affected by genotype (G; i.e. 5-Htt +/− versus wild-type), the environment (E; i.e. prenatal stress versus control) or in an interactive manner (GxE; i.e. indicating those genes of which the effect of the environment depends upon the genotype).

Factor	DAVID ID	Name	# Genes	P-Value
Genotype (G)	mmu04010	MAPK signaling pathway	26	1.79E-10
	mmu05200	Pathways in cancer	20	0.011
	mmu05412	Arrhythmogenic right ventricular cardiomyopathy (ARVC)	8	0.012
	mmu04810	Regulation of actin cytoskeleton	15	0.013
	mmu05410	Hypertrophic cardiomyopathy (HCM)	8	0.021
	mmu04722	Neurotrophin signaling pathway	10	0.029
	mmu05218	Melanoma	7	0.030
	mmu05414	Dilated cardiomyopathy	8	0.033
	mmu04114	Oocyte meiosis	9	0.037
	mmu04142	Lysosome	9	0.044
Environment (E)	mmu04010	MAPK signaling pathway	24	5.24E-11
	mmu04510	Focal adhesion	18	0.003
	mmu04012	ErbB signaling pathway	10	0.009
	mmu04722	Neurotrophin signaling pathway	12	0.018
	mmu04150	mTOR signaling pathway	7	0.023
	mmu00052	Galactose metabolism	5	0.024
	mmu05219	Bladder cancer	6	0.028
	mmu04520	Adherens junction	8	0.037
	mmu04114	Oocyte meiosis	10	0.047
G×E	mmu04060	Cytokine-cytokine receptor interaction	12	0.009
	mmu04310	Wnt signaling pathway	9	0.009
	mmu04672	Intestinal immune network for IgA production	5	0.022
	mmu04640	Hematopoietic cell lineage	6	0.023
	mmu05310	Asthma	4	0.027
	mmu05330	Allograft rejection	5	0.028
	mmu04012	ErbB signaling pathway	6	0.029
	mmu04514	Cell adhesion molecules (CAM)	8	0.033
	mmu04510	Focal adhesion	9	0.043
	mmu04360	Axon guidance	7	0.045

1 **Acquired using DAVID analysis.**

#### Gene expression changes accompanying variations in 5-Htt genotype

Of the 773 genes affected by the 5-Htt +/− genotype, 300 genes were upregulated and 473 downregulated (Figure 5; also see Text S1 for a complete overview of all genes regulated by 5-Htt genotype). Amongst others, DAVID analysis revealed the mitogen-activated protein kinase (MAPK) signaling pathway and neurotrophin signaling as significantly overrepresented pathways affected by G (see Table 3 for a complete overview of the functionally enriched pathways). In the MAPK signaling pathway, 73% of the 26 genes were downregulated, such as *fibroblast growth factor 1 (Fgf1), calcium channel voltage dependent, L type, alpha 1D subunit (Cacna1d)* and *mitogen-activated protein kinase 8 interacting protein 3* (*Mapk8ip3*; see Table 4 for an overview of all genes affected in the MAPK signaling pathway). In the neurotrophin signaling pathway, 10 genes were significantly affected, among which was the *neurotrophic tyrosine kinase*, *receptor type 2 (Ntrk2*; also known as *TrkB receptor*) and *transformation related protein 53 (Trp53*; see Table 4 for a complete overview). Of note, the MAPK and neurotrophin signaling KEGG pathway partially overlap. One gene, namely the *v-crk sarcoma virus CT10 oncogene homolog (avian; Crk)*, was affected in both KEGG pathways. Of note, the expression of the *solute carrier family 6*, *member 4* (*Slc6a4*, encoding for the 5-Htt) was upregulated in 5Htt +/− mice 10.6 fold indicating an upregulation of the allele leading to a truncated protein (see [38]).

**Table 4:** Differentially expressed genes within the mitogen-activated protein kinase (MAPK) signaling and neurotrophin signaling pathways (5-Htt +/− versus wild-type).

MAPK signaling pathway						
Effect	Affy ID	DAVID Gene name	Entrez Symbol	# (%) Genes	FC^1^	P-Value
**up**	1432647_at	*epidermal growth factor receptor*	*Egfr*		1.27	0.007
	1444344_at	*fibroblast growth factor 10*	*Fgf10*		1.22	0.007
	1460296_a_at	*fibroblast growth factor 22*	*Fgf22*		1.26	0.005
	1421473_at	*interleukin 1 alpha*	*Il1a*		1.17	0.005
	1452383_at	*ribosomal protein S6 kinase polypeptide 3*	*Rps6ka3*		1.21	0.009
	1449901_a_at	*mitogen-activated protein kinase kinase kinase 6*	*Map3k6*		1.24	0.007
	1460176_at	*v-crk sarcoma virus CT10 oncogene homolog (avian)*	*Crk*	**7 (27%)**	1.39	4.68E-04
**down**	1438031_at	*RAS, guanyl releasing protein 3*	*Rasgrp3*		1.27	0.003
	1421297_a_at	*calcium channel, voltage-dependent, L type, alpha 1C subunit*	*Cacna1c*		1.17	0.008
	1428051_a_at	*calcium channel, voltage-dependent, L type, alpha 1D subunit*	*Cacna1d*		1.21	0.004
	1425812_a_at	*calcium channel, voltage-dependent, N type, alpha 1B subunit*	*Cacna1b*		1.37	0.004
	1450520_at	*calcium channel, voltage-dependent, gamma subunit 3*	*Cacng3*		1.33	0.001
	1450869_at	*fibroblast growth factor 1*	*Fgf1*		1.19	0.004
	1418498_at	*fibroblast growth factor 13*	*Fgf13*		1.35	0.009
	1425911_a_at	*fibroblast growth factor receptor 1*	*Fgfr1*		1.34	0.003
	1427776_a_at	*fibroblast growth factor receptor 4*	*Fgfr4*		1.26	0.003
	1426677_at	*filamin, alpha*	*Flna*		1.15	0.009
	1450097_s_at	*guanine nucleotide binding protein, alpha 12*	*Gna12*		1.27	0.002
	1417885_at	*microtubule-associated protein tau*	*Mapt*		1.29	0.001
	1425679_a_at	*mitogen-activated protein kinase 8 interacting protein 1*	*MAPK8ip1*		1.19	0.008
	1416437_a_at	*mitogen-activated protein kinase 8 interacting protein 3*	*MAPK8ip3*		1.32	0.003
	1421446_at	*protein kinase C, gamma*	*Prkcc*		1.39	6.73E-04
	1424287_at	*protein kinase, X-linked*	*Prkx*		1.30	0.004
	1450368_a_at	*protein phosphatase 3, regulatory subunit B, alpha isoform (calcineurin B, type I)*	*Ppp3r1*		1.36	0.007
	1427739_a_at	*transformation related protein 53*	*Trp53*		1.28	0.010
	1420837_at	*neurotrophic tyrosine kinase, receptor, type 2*	*Ntrk2*	**19 (73%)**	1.57	2.93E-04
**Total**				**26**		
**Neurotrophin signaling pathway**						
**up**	1437122_at	*predicted gene 3655; B-cell leukemia/lymphoma 2*	*Bcl2*		1.16	0.008
	1454378_at	*predicted gene, EG546165; predicted gene 2423; hypothetical protein LOC674211; tyrosine 3-monooxygenase/tryptophan 5-monooxygenase activation protein, theta polypeptide*	*Ywhaq*		1.78	0.008
	1452383_at	*ribosomal protein S6 kinase polypeptide 3*	*Rps6ka3*		1.21	0.009
	1439005_x_at	*tyrosine 3-monooxygenase/tryptophan 5-monooxygenase activation protein, zeta polypeptide; predicted gene 4202*	*Ywhaz* *		1.26	0.009
	1460176_at	*v-crk sarcoma virus CT10 oncogene homolog (avian)*	*Crk*	**5 (45%)**	1.39	4.68E-04
**down**	1448668_a_at	*interleukin-1 receptor-associated kinase 1*	*Irak1*		1.20	0.005
	1420837_at	*neurotrophic tyrosine kinase, receptor, type 2*	*Ntrk2*		1.57	2.93E-04
	1425070_at	*neurotrophic tyrosine kinase, receptor, type 3; similar to neurotrophic tyrosine kinase, receptor, type 3*	*Ntrk3*		1.23	0.008
	1427739_a_at	*transformation related protein 53*	*Trp53*		1.28	0.010
	1452325_at	*transformation related protein 73*	*Trp73*		1.21	0.008
	1448218_s_at	*tyrosine 3-monooxygenase/tryptophan 5-monooxygenase activation protein, zeta polypeptid; predicted gene 4202*	*Ywhaz* *	**6 (55%)**	1.24	0.004
**Total**				**11**		

1 **Abbreviation: FC, fold change.**

*Probesets recognize different transcripts.

#### Gene expression changes induced by PS exposure

The expression of 960 genes was changed by PS exposure, of which 462 were upregulated and 498 downregulated (Figure 5; also see Text S2 for a complete overview of all genes regulated by PS exposure). Furthermore, 9 KEGG pathways with significant P-values were detected by DAVID analysis (see Table 3). As the 5Htt +/− genotype, PS affected the MAPK and the neurotrophin signaling pathway (Table 5; see Table 3 for a complete overview of the functionally enriched pathways). The *calcium channel, voltage-dependent, gamma subunit 3 (Cacng3)* and *protein kinase C, gamma (PrKcc)* are examples of genes negatively influenced by both G and E.

**Table 5:** Differentially expressed genes within the mitogen-activated protein kinase (MAPK) signaling and neurotrophin signaling pathways (PS versus control).

MAPK signaling pathway						
Effect	Affy ID	DAVID Gene name	Entrez Symbol	# (%) Genes	FC^1^	P-Value
**up**	1444199_at	*ELK4, member of ETS oncogene family*	*Elk4*		1.16	0.007
	1423100_at	*FBJ osteosarcoma oncogene*	*Fos*		1.76	0.001
	1417409_at	*Jun oncogene*	*Jun*		1.22	0.005
	1438992_x_at	*activating transcription factor 4*	*Atf4*		1.30	1.16E-04
	1447511_at	*calcium channel, voltage-dependent, N type, alpha 1B subunit*	*Cacna1b*		1.28	0.006
	1420287_at	*calcium channel, voltage-dependent, P/Q type, alpha 1A subunit*	*Cacna1a*		1.32	7.17E-04
	1449773_s_at	*growth arrest and DNA-damage-inducible 45 beta*	*Gadd45b*		1.19	0.003
	1452318_a_at	*heat shock protein 1B*	*Hspa1b*		1.35	0.001
	1448950_at	*interleukin 1 receptor, type I*	*Il1r1*		1.20	0.006
	1443540_at	*mitogen-activated protein kinase kinase kinase 1*	*Map3k1*		1.24	0.009
	1438908_at	*mitogen-activated protein kinase kinase kinase 12*	*Map3k12*		1.19	0.003
	1447667_x_at	*mitogen-activated protein kinase kinase kinase 4*	*Map3k4*		1.14	0.010
	1456467_s_at	*nemo like kinase*	*Nlk*		1.15	0.007
	1421416_at	*predicted gene 14378; similar to transforming growth factor, beta receptor III (betaglycan, 300kDa); mitogen-activated protein kinase kinase 7*	*Map2k7*	**14 (58%)**	1.18	0.004
**down**	1447941_x_at	*Braf transforming gene*	*Braf*		1.21	0.006
	1450520_at	*calcium channel, voltage-dependent, gamma subunit 3*	*Cacng3*		1.34	3.88E-04
	1424932_at	*epidermal growth factor receptor*	*Egfr*		1.13	0.007
	1425911_a_at	*fibroblast growth factor receptor 1*	*Fgfr1*		1.27	0.006
	1426677_at	*filamin, alpha*	*Flna*		1.19	0.001
	1453712_a_at	*mitogen-activated protein kinase kinase 5*	*Map2k5*		1.22	0.010
	1455441_at	*mitogen-activated protein kinase kinase kinase 7; predicted gene 8188*	*Map3k7*		1.23	0.005
	1421446_at	*protein kinase C, gamma*	*Prkcc*		1.31	0.001
	1451943_a_at	*protein phosphatase 1A, magnesium dependent, alpha isoform*	*Ppm1a*		1.18	0.006
	1429759_at	*ribosomal protein S6 kinase polypeptide 6*	*Rps6ka6*	**10 (42%)**	1.31	1.48E-04
**Total**				**24**		
**Neurotrophin signaling pathway**						
**up**	1417409_at	*Jun oncogene*	*Jun*		1.21	0.005
	1438992_x_at	*activating transcription factor 4*	*Atf4*		1.29	1.16E-04
	1443540_at	*mitogen-activated protein kinase kinase kinase 1*	*Map3k1*		1.24	0.009
	1425514_at	*phosphatidylinositol 3-kinase, regulatory subunit, polypeptide 1 (p85 alpha)*	*Pik3r1*		1.18	0.005
	1421416_at	*predicted gene 14378; similar to transforming growth factor, beta receptor III (betaglycan, 300kDa); mitogen-activated protein kinase kinase 7*	*Map2k7*		1.18	0.004
	1454378_at	*predicted gene, EG546165; predicted gene 2423; hypothetical protein LOC674211; tyrosine 3-monooxygenase/tryptophan 5-monooxygenase activation protein, theta polypeptide*	*Ywhaq*		1.79	0.004
	1439005_x_at	*tyrosine 3-monooxygenase/tryptophan 5-monooxygenase activation protein, zeta polypeptide*	*Ywhaz*		1.36	5.72E-04
	1436981_a_at	*tyrosine 3-monooxygenase/tryptophan 5-monooxygenase activation protein, zeta polypeptide*	*Ywhaz*	**8 (62%)**	1.28	0.005
**down**	1447941_x_at	*Braf transforming gene*	*Braf*		1.21	0.006
	1455869_at	*calcium/calmodulin-dependent protein kinase II, beta*	*Camk2b*		1.77	9.13E-04
	1453712_a_at	*mitogen-activated protein kinase kinase 5*	*Map2k5*		1.22	0.010
	1429759_at	*ribosomal protein S6 kinase polypeptide 6*	*Rps6ka6*		1.31	1.48E-04
	1438839_a_at	*tyrosine 3-monooxygenase/tryptophan 5-monooxygenase activation protein, epsilon polypeptide*	*Ywhae*	**5 (38%)**	1.19	0.008
**Total**				**13**		

1 **Abbreviation: FC, fold change.**

#### Gene expression profiles indicating a gene×environment interaction

At the G×E level, 651 genes showed a significant expression pattern (Figure 5; see Text S3 for a complete overview of all genes regulated in a G×E fashion). DAVID pathway analysis revealed that G×E significantly enriched 10 pathways (Table 3). The most significantly enriched biological process was cytokine-cytokine receptor interactions (Table 6; see Table 3 for a complete overview of the functionally enriched pathways). Examples of genes affected within this pathway are *interleukin 4 (Il4), interleukin 12a (Il12a)* and *tumor necrosis factor receptor superfamily, member 1a (Tnfrsf1a)*. Another pathway significantly influenced in a G×E manner was the Wnt signaling pathway amongst which the *RAS-related C3 botulinum substrate 2 (Rac2), Rho-associated coiled-coil containing protein kinase 1 (Rock1), calcium/calmodulin-dependent protein kinase II, delta (Camk2d)* and *presenilin 1(Psen1)* were differentially expressed.

**Table 6:** Differentially expressed genes within the cytokine-cytokine receptor interactions and Wnt signaling pathways involving genes regulated in a genotype (G)×environment (E) manner, i.e. indicating those genes of which the effect of the (prenatal) environment depends upon the 5-Htt genotype).

Cytokine-cytokine receptor interactions			
Affy ID	DAVID Gene name	Entrez Symbol	P-Value
1437382_at	*activin receptor IIA*	*Acvr2a*	0.004
1421188_at	*chemokine (C-C motif) receptor 2*	*Ccr2*	0.003
1421843_at	*interleukin 1 receptor accessory protein*	*Il1rap*	0.005
1425454_a_at	*interleukin 12a*	*Il12a*	0.004
1422397_a_at	*interleukin 15 receptor, alpha chain*	*Il15ra*	0.008
1449864_at	*interleukin 4*	*Il4*	0.009
1415855_at	*kit ligand*	*Kitl*	0.006
1450272_at	*tumor necrosis factor (ligand) superfamily, member 8*	*Tnfsf8*	0.009
1430259_at	*tumor necrosis factor receptor superfamily, member 11a*	*Tnfrsf11a*	0.003
1427600_at	*tumor necrosis factor receptor superfamily, member 19*	*Tnfrsf19*	0.005
1417291_at	*tumor necrosis factor receptor superfamily, member 1a*	*Tnfrsf1a*	0.006
1448951_at	*tumor necrosis factor receptor superfamily, member 1b*	*Tnfrsf1b*	0.007
**Wnt signaling pathway**			
1417620_at	*RAS-related C3 botulinum substrate 2*	*Rac2*	4.86E-04
1441162_at	*Rho-associated coiled-coil containing protein kinase 1*	*Rock1*	0.007
1422659_at	*calcium/calmodulin-dependent protein kinase II, delta*	*Camk2d*	0.009
1449730_s_at	*frizzled homolog 3 (Drosophila)*	*Fzd3*	0.007
1458002_at	*mitogen-activated protein kinase 10*	*Mapk10*	0.002
1434275_at	*naked cuticle 2 homolog (Drosophila)*	*Nkd2*	0.007
1425549_at	*presenilin 1*	*Psen1*	0.005
1443270_at	*prickle-like 2 (Drosophila)*	*Prickle2*	0.005

#### Validation of microarrays by qRT-PCR

Using qRT-PCR we attempted to confirm the microarray data. Of the 8 genes, 6 were replicated in terms of the overall G, E or G×E effects, such as *Fos* (F_3,32_ = 7.076; P = 0.012), *Mbp* (G effect: F_3,32_ = 9.421; P = 0.004; E effect: F_3,32_ = 12.152; P = 0.001; G×E effect: F_3,32_ = 4.897; P = 0.034), *Ppp1r1b* (F_3,32_ = 9.055; P = 0.005), *Trhr* (E effect: F_3,32_ = 5.087; P = 0.031; G×E effect: F_3,32_ = 15.599; P<0.001), *Xaf1* (F_3,32_ = 102.615; P<0.001) and *Zzef1* (F_3,32_ = 14.128; P = 0.001). For the *Kcnip2* (G effect: F_3,32_ = 2.048; P = 0.162; G×E effect: F_3,32_ = 5.612; P = 0.024), the overall effect of 5-Htt genotype and for the *Phox2a* (F_3,30_ = 2.871; P = 0.101) the overall effect of PS was not replicated (see table 7 for more details).

**Table 7:** Genes validated by qRT-PCR.

Gene	WTFC	WTFPS	5-Htt +/− FC	5-Htt +/− FPS	Main effect(s) Microarray	Main effect(s) qRT-PCR
*Fos*	100±22.2	123.9±10.5	94.3±14.0	137.7±15.6	E (↑)	E (↑)
*Kcnip2*	100±5.3	97.6±6.8	86.1±5.4	101.0±3.8	G (↓)	G×E
*Mbp*	100±4.7	127.4±7.7	95.9±4.9	102.0±5.8	G (↓)	G (↓), E (↑), G×E
*Phox2a*	100±20.0	98.6±9.2	77.4±11.6	118.6±19.7	E (↑)	-
*Ppp1r1b*	100±14.3	118.4±13.6	89.1±8.2	83.6±2.9	G (↓)	G (↓)
*Trhr*	100±20.4	51.2±20.8	74.9±9.5	88.2±8.3	G×E	E (↓), G×E
*Xaf1*	100±14.3	478.0±117.9	2582.7±8.3	2599.3±9.4	G (↑)	G (↑)
*Zzef1*	100±3.5	102.7±3.7	86.6±5.2	91.3±6.1	G (↓)	G (↓)

**Values indicate average expression as percentage of wild-type female control offspring. Data represent mean ± S.E.M. Abbreviations: WT, wild-type; F, females; C, control offspring; PS, prenatally stressed offspring.**

## Discussion

The present study demonstrates that exposure of 5-Htt +/− mice to prenatal maternal stress is associated with increased depressive-like behavior, an effect that appeared to be more pronounced in female offspring. Conversely, adult 5-Htt +/− mice showed enhanced memory performance as well as signs of reduced anxiety as compared to WT offspring. Further, female 5-Htt genotype, PS and their interaction were associated with distinct hippocampal gene expression profiles, the implications of which are discussed in more detail below.

### Combined behavioral effects of offspring 5-Htt genotype and PS

This is the first study assessing the effects of developmental stress exposure on adult cognition, anxiety and depressive-like behavior as well as HPA axis responsivity in both male and female 5-Htt +/− offspring.

While, 5-Htt +/− mice showed improved memory performance in the ORT when compared to WT offspring, PS exposure seemed to impair object memory performance in the same task. Further, rather unexpectedly, 5-Htt +/− mice seemed to be less anxious when compared to WT mice, as indicated by an increased time spent in the open arms of the EZM. While PS exposure did not affect this particular type of anxiety-like behavior, it did reduce exploratory behavior in the same task, as indicated by a reduced distance moved, primarily in WT offspring. In addition, exposure to the FST was associated with increased depressive-like behavior in PS mice, an effect which appeared to be particularly manifest in 5-Htt +/− female offspring. Furthermore, 5-Htt +/− mice showed reduced basal CORT levels when compared to WT offspring.

While, the 5-Htt +/− genotype conveyed beneficial effects in the ORT, PS exposure seemed to impair object memory performance, which is in line with previous observations [22]. The ‘protective’ genotype effect is in contrast with a previous study by Olivier et al. [39], which showed that 5-Htt +/− rats have impaired object memory when using longer intervals in the ORT. Evidence for a role of the human 5-HTT genotype in learning and memory is limited. In line with our data, Roiser and colleagues [40] found that individuals homozygous for the 5HTTLPR s-allele show improved memory and attention as compared to ll-carriers. Recently, it has been suggested that the beneficial cognitive effects of the s-allele may explain why genetic variation resulting in low 5-HTT function has been maintained throughout evolution [34]. The exact role of the 5-HTT in cognition remains to be elucidated, though. For example, it would be of particular interest to study the effects of 5-Htt genotype variation in spatial, hippocampus-dependent memory processing, by e.g. using a spatial variant of the ORT, i.e., the object location test (OLT; see [41]).

A recent study by Heiming et al. [18], which assessed exposure of 5-Htt +/− females to olfactory cues of unfamiliar adult males during pregnancy and lactation did not reveal any differences in anxiety between 5-Htt +/− and WT offspring. Another recent investigation by Jones and coworkers [42] showed that offspring of both 5-Htt +/− and WT dams stressed during pregnancy showed signs of decreased anxiety in the elevated plus maze (increased time spent in the open arms; as compared to control offspring), whereas, at the same time, maternal stress exposure increased anxiety-like behavior in the open field test (reduced time spent in the centre) in offspring from WT mothers only. Another study by Carola and colleagues [19] showed clear signs of evidence for increased anxiety in male 5-Htt +/− mice when exposed to low levels of maternal care during early life. The observed discrepancy between the various investigations might be explained by the different study designs, involving e.g. diverse breeding schemes, concomitant with 1) variations in maternal and/or paternal genotype, 2) different experimentally induced variations in the pre- and postnatal environment, and 3) a distinct age at which the behavioral testing was performed. For example, in the study by Heiming et al. [18], 5-Htt +/− dams were exposed to stress during both pregnancy and the postpartum period. In the study by Jones and colleagues [42], next to a different breeding design, a chronic variable stress paradigm was used, starting from gestational day 6 and lasting until parturition. In addition, offspring genotype was not taken into account in that study, which makes accurate interpretation of the data difficult. In the study by Carola et al. [19], reciprocal inter-crossing between C57BL/6J and BALB/cByJ was applied, in order to assess the effect of variations in maternal care. Both strains carried different alleles of the serotonin synthesizing enzyme Tph2, thereby introducing additional genetic variation to the study design. In fact, the Tph2 genotype significantly affected the behavioral outcome, indicating a G×G×E effect. Furthermore, only WT mothers were used for breeding in that study. All in all, when it comes to adult anxiety-like behavior, exposure of 5-Htt +/− mice to developmental stress may have various, differential, complex programming effects, the nature of which is dependent on numerous factors.

Interestingly, predominantly male 5-Htt +/− mice appeared to have lower basal CORT levels when compared to WT offspring, while, conversely, female 5-Htt +/− mice appeared to have enlarged adrenals as compared to WT mice. Thus, it is tempting to speculate that the 5-Htt +/− genotype is associated with a sex-dependent alteration in the set-point of the HPA axis, which, in turn, may be related to the different vulnerability of both sexes when it comes to e.g. developmental stress exposure. In this context, Wuest and colleagues revealed that male ss-allele carriers display the lowest cortisol awakening response (an indirect assessment of the basal cortisol secretion) when compared to sl- and ll-carriers, whereas female ss-carriers showed the highest [43]. Whether the decreased basal CORT levels in 5-Htt +/− offspring contribute to the reduced levels of anxiety as seen in the EZM in 5-Htt +/− mice remains to be elucidated.

#### Adaptive capacity of variations in the 5-Htt genotype

All in all, the enhanced memory performance and reduced anxiety as seen in 5-Htt +/− mice underscore the adaptive capacity of this specific genetic variation [34]. In fact, along similar lines, when looking more closely at the FST data, control 5-Htt +/− offspring even seemed to show lower levels of depressive-like behavior when compared to WT mice, while only 5-Htt +/− mice exposed to PS showed increased levels of depressive-like behavior. Taken together, these findings reiterate the notion that the classical deficit-oriented association of the 5-HTTLPR variants may be oversimplified. In effect, our current data suggest that variation in the 5-Htt genotype acts in concert with variations in the pre-/perinatal environment, thereby determining (i.e. programming), in a sex-specific manner, whether a response to an acute environmental challenge in adulthood (e.g. cognitive and/or emotional) will turn out to be positive or negative.

Altogether, the current data suggest that although the 5-Htt +/− genotype shows clear adaptive capacity, it at the same time seems to increase the vulnerability to developmental stress exposure, predominantly in female offspring.

### Hippocampal gene expression profiles

Microarray analysis revealed various effects of female 5-Htt genotype, PS, and their interaction on hippocampal gene expression profiles, which may, at least in part, explain the distinct behavioral phenotypes observed among the different experimental groups. Below, we discuss the role of several relevant genes and biological pathways in more detail.

#### MAPK signaling

DAVID analysis revealed an overall negative effect of the 5-Htt +/− genotype and a neutral to positive effect of PS on the MAPK signaling pathway within the female hippocampus. MAPK signaling is known to play an important role in embryogenesis, cell differentiation, cell proliferation and cell death [44], [45], whereas aberrant signaling has been implicated in the course and development of several psychiatric disorders [46], [47]. The MAPK pathway comprises three major signaling cascades, i.e. the extracellular signal-regulated kinases 1 and 2 (ERK 1/2) cascade, the c-Jun N-terminal kinases (JNK) cascade and the p38 pathway, all of which are regulated in a complex, interactive manner. Downstream signaling is mediated via a kinase phosphorylation cascade culminating in the activation of transcription factors and the expression of specific genes.

When looking more closely to effects of the 5-Htt +/− genotype and PS exposure, we found that PS, but not the 5-Htt +/− genotype, exerts a strong positive impact on the JNK cascade, whereas the p38 cascade remains nearly unaffected. Liu *et al.* [48] and Meller *et al.* [49] showed a similar effect after acute stress in both the mouse and rat hippocampus. We found three JNK-activating genes, the *interleukin 1 receptor, type 1 (Il1r1), mitogen-activated protein kinase kinase kinase 12 (Map3k12*, also known as *Muk)* and *mitogen-activated protein kinase kinase 7 (Map2k7*, also known as *Mkk7)* and one Jnk-substrate, *jun oncogene (Jun*, also known as *c-Jun*) being up-regulated by PS. Further, both the 5Htt +/− genotype and PS exhibited an overall negative effect on the ERK1/2 cascade. Genes which were down-regulated by both factors are e.g. the *calcium channel, voltage-dependent, gamma subunit 3 (Cacng3), protein kinase C (Prkcc)* and the *fibroblast growth factor receptor 1 (Fgfr1)*. In addition, the 5Htt +/− genotype reduced the expression of *fibroblast growth factor 1 (Fgf1)*, the major ligand of *Fgfr1*. Interestingly, a dysfunction in FGF signaling has been suggested to play an important role in the etiology of mood disorders [50], which is underscored by the finding that patients with major depression disorder (MDD) show an increased *FGFR1* expression in various hippocampal subregions [51]. In addition, the expression of *Fos*, a recognized transcription factor, which represents a critical downstream target of the ERK pathway, was increased after PS exposure (an effect which was validated by RT-PCR).

#### Neurotrophin signaling pathway

The neurotrophin family of proteins consists of BDNF, nerve growth factor (NGF), neurotrophin 3 (NT-3), and neurotrophin 4 (NT-4), which bind to the tyrosine kinase (Trk receptor family (including TrkA, TrkB and TrkC as well as to the p75 neurotrophin receptor; p75^NTR^), leading to the activation of different downstream signaling cascades which modulate neuronal and synaptic plasticity. Thus, the neurotrophin signaling pathway is implicated in the etiology and therapy of depression (“neurotrophin hypothesis of depression”; [52], [53]). We found that both the 5-Htt +/− genotype and PS exposure significantly affect neurotrophin signaling indicating that both genetic and environmental factors contribute to dynamic neuronal and synaptic plasticity in the hippocampus. More specifically, TrkB signaling was affected by both the 5Htt +/− genotype and PS. For example, *Ntrk2 (TrkB)* receptor expression itself was decreased in 5-Htt +/− offspring. Next to its essential role in promoting long-term potentiation, hippocampal TrkB signaling is critical in cell survival ([54]; see below). The TrkB receptor is activated by Bdnf and controls three different major pathways, the P13 kinase cascade, the PLC-γ1 cascade and the ERK1/2 MAPK cascade [55], the last one of which was predominantly affected by both 5-Htt +/− and PS. This cascade targets Creb1 whose nuclear activation is an important component of a general switch that converts short-term into long-term plasticity [56]. Before inducing Creb1-dependent transcription, its transcriptional repressor Creb2 (or activating transcription factor 4; Atf4) has to be released. Consequently, it could be postulated that the observed decrease in *Ntrk2* expression in 5-Htt +/− animals, in combination with the increase in *Creb2* expression, induced by PS exposure, contributed to impaired Bdnf signaling and related neuronal and synaptic plasticity, thereby eliciting depressive-like behavior [57].

While both 5-Htt +/− genotype and PS seem to impair TrkB signaling in a similar manner, G and E seem to affect p75^NTR^ signaling in an opposite way. The majority of genes affected by the 5-Htt +/− genotype showed decreased expression patterns, among which the *transformation-related protein 73 (Trp73), transformation-related protein 53 (Trp53), tyrosine 3-monooxygenase/tryptophan 5-monooxygenase activation protein, zeta polypeptide (Ywhaz)*, and the *interleukin-1 receptor-associated kinase 1 (Irak1)*. On the other hand, the vast majority of genes affected by PS in this pathway showed increased expression patterns, among which the *mitogen-activated protein kinase kinase kinase 1 (Map3k1*, also known as *Mekk1*), *mitogen-activated protein kinase kinase 7 (Map2k7*, also known as *Mekk7*) and *c-Jun*. Activation of the P75^NTR^ pathway is suggested to play a prodepressive role [58]. In more detail the activation of the P75^NTR^ pathway has been linked to an increase in long term depression (LTD) [59]. Emerging evidence supports the idea that LTD may have a role in regulating stress- and depressive-like behavior. For example, several studies reported a correlation between (behavioral) stress and the induction of LTD in adult rats [60], [61]. Moreover, chronic mild stress–induced LTD could be reversed by chronic treatment by antidepressant treatment [61]. All in all, hypothetically, dysfunctional neurotrophin signaling, might mediate, at least in part, the altered depressive-like behavior observed in PS 5-Htt +/− mice.

#### Cytokine-Cytokine receptor interaction

Although the 5-Htt +/− genotype and PS exposure often act on distinct specific molecular targets, there is a considerable degree of overlap when it comes to the biological signaling pathways they affect (*independently* from each other). When considering hippocampal gene expression profiles that indicate a G×E interaction, i.e. those genes of which the regulatory effect of PS is *dependent* upon the 5-Htt genotype, a different pattern is observed. In this respect, our data suggest that cytokine-cytokine receptor interactions play a vital role when a dysfunctional 5-HT system and stress interact. This idea is supported by the study of Fredericks *et al.* [62] who found that healthy women homozygous for the s-allele of the 5-HTTLPR have elevated pro-inflammatory cytokine levels and a higher IL-6/IL-10 ratio both at baseline and during stress, when compared to (ll) individuals. Interestingly, the risk for developing a clinically relevant depression after cytokine therapy is increased in people who carry the s-allele [63]. Further, the pro-inflammatory cytokine level of patients suffering from major depression is higher compared to non-depressed individuals [64]–[67]. However, treatment with selective serotonin reuptake inhibitors (SSRIs) is able to reduce this enhancement [65], [68]. In addition, after experimental or therapeutic administration of pro-inflammatory cytokines, humans with originally no signs of depression, display depressive symptoms [69]–[72]. For example, about half of all patients treated for a long period with interferon get depressed, and this state of mood can be meliorated by SSRI treatment [73]. Interestingly, it has been suggested that interferon-induced immune activation on depression may be explained in part by alterations in neurotrophin signaling capacity, reflected by decreases in serum BDNF following interferon treatment [74]. In the present study neurotrophin signaling was affected by both G and E. In this context, it has been suggested that pro-inflammatory cytokines like interferon-γ (INF-γ) and tumour necrosis factor α (TNFα) reduce the availability of tryptophan, the precursor for 5-HT, by inducing indoleamine-2,3-dioxygenase (IDO) [75]–[77]. In addition, the expression of several members of the Tnf and Tnf receptor superfamily, such as *Tnfsf8*, *Tnfrsf11a*, *Tnfrsf1a*, *Tnfrsf1b*, was regulated in a G×E manner. Further evidence for a molecular interaction between 5-HT and cytokines is given by the observation that mice lacking the interleukin-15 receptor (Il15ra), the expression of which was also affected within our model in a G×E manner, showed increased depressive-like behavior, whereas fluoxetine was able to reduce it. These Il15ra knockout mice showed decreased hippocampal expression of 5-Ht_1A_ receptor, increased hippocampal expression of 5-Ht_2C_, and region-specific changes of 5Htt immunoreactivity [78]. Furthermore, the lack of Il15ra resulted in reduced anxiety in these mice [79], which indicates a comparable behavioral phenotype as observed in the present study. All in all, it may be hypothesized that, when challenged by e.g. developmental stress exposure, a dysfunctional 5-HT system could lead to a disturbed cytokine balance thereby increasing the vulnerability to stress, eventually resulting in psychiatric conditions.

#### Wnt signaling pathway

Next to cytokine-cytokine receptor interactions, also Wnt signaling was significantly affected in a G×E manner. Wnt proteins are required for basic developmental processes and act via at least 3 different Wnt pathways: the canonical pathway, the planar cell polarity (PCP) pathway and the Wnt/Ca^2+^ pathway [80]. All 3 cascades are initiated via Wnt binding to frizzled (Fzd). Interestingly, the mRNA expression of *frizzled homolog 3 (Fzd3)* was altered in the present study in a G×E fashion, indicating that all three abovementioned cascades are influenced in our model. Furthermore, our microarray analysis revealed 4 other affected genes in the PCP signaling pathway, among which were *Ras-related C3 botulinum substrate 2 (Rac2)* and *Rho-associated coiled-coil containing protein kinase 1 (Rock1)*. With respect to this pathway, binding to Fzd3 leads to the activation of the small GTPases RhoA and Rac1, which activate the stress kinase Jnk (Jun N-terminal kinase) and Rock, which eventually induces remodelling of the cytoskeleton and associated changes in cell adhesion and motility. The canonical Wnt pathway and especially 2 of its key players, glycogen synthase kinase 3beta (GSK-3beta) and beta-catenin, have been highly implicated in the etiology of psychiatric disorders such as depression, schizophrenia and bipolar disorder [81]–[84]. Moreover, decreased Gsk-3beta and increased beta-catenin levels in the mouse brain have been associated with a better performance in the FST [81]. Although these two key players were not directly affected in terms of gene expression within our 5-Htt×PS model, the mRNA expression of *presenilin1 (psen1)*, whose protein product is known to bind and stabilize beta-catenin [85], was regulated in a G×E fashion.

#### *Xaf1*, *Mbp* and *Trhr*

Quantitative RT-PCR validation indicated significant genotype effects for e.g. *Xaf1*, *Mbp* and *Trhr*. Xaf1 is known to play an important role in programmed cell death by inhibiting the anti-apoptotic functions of the X-linked inhibitor of apoptosis (XIAP) and of other members of the family of inhibitors of apoptosis (IAP), like survivin [86]. Xiap has trophic effects on hippocampal neurons by increasing *Bdnf* and *TrkB* activity [87]. Thus, the observed increase in *Xaf1* and the decrease in *TrkB* expression (see above) in adult 5-Htt +/− mice suggest a decreased resistance to apoptosis in these animals. Previous investigations by Ravary *et al.* [38] and Persico *et al.* [88] showed no signs of increased neuronal apoptosis in 5-Htt +/− versus WT mice. Whether additional environmental stress exposure affects the levels of apoptosis in 5-Htt +/− animals remains to be elucidated. Next to *Xaf1*, the effects of e.g. *Mbp* and *Trhr* were also validated by RT-PCR. Mbp is known to be involved in myelinisation of the central nervous system and has recently been associated with schizophrenia [89]. Trhr plays a role in the hormone system and neuromodulation. Interestingly, mice with a deficiency for the *Trhr1* show an anxiety- and depressive-like behavior [90]. Evidently, the implications of the present findings await further research.

### Limitations

It should be noted that the animals were housed individually from weaning onwards in order to prevent the establishment of a hierarchy. Although the cages were in close proximity to each other (enabling visual contact between neighbouring mice) and the home cage was enriched with paper tissues and a cardboard tube, the isolated housing conditions could represent an additional stressor to the animals. Further, it is likely that the behavioral testing paradigms exerted an independent effect on hippocampal gene expression profiles. Although behavioral task exposure was identical for all groups, one cannot exclude that the animals' response to it was different among groups. Thus, behavioral testing may have left a permanent imprint on hippocampal gene expression patterns in a genotype- and/or condition-dependent manner. Evidently, examining behavior and its underlying biological mechanisms in the same set of animals enables the possibility of linking both features in a more direct way. Similarly, prior behavioral testing may have had an influence on the basal and stress-induced CORT levels. Nevertheless, an acute effect can be excluded, since the animals were left undisturbed for a week in between behavioral test sessions and blood sampling. Another remarkable notion is the fact that PS was associated with a higher degree of mortality in the offspring. Although a similar effect has been reported previously (e.g. [91]), in the present study this effect was observed immediately after weaning, when the offspring were moved to IVC cages. Personal observations suggest that the affected mice were too small and weak to force enough water through the IVC sipper tubes. In light of previous work from our group, which has shown a direct correlation between low birth weight –as a consequence of restricted fetal growth associated with PS exposure– and, amongst others, adult depressive-like behavior [4], the consequent loss of these mice in the behavioral comparison later in life may have even weakened some of the observed behavioral effects of PS.

### Conclusion

Taken together, the present data suggest that the 5-Htt +/− genotype is associated with improved object memory function as well as signs of reduced anxiety. In contrast, exposure of 5-Htt +/− mice to PS was associated with increased depressive-like behavior, an effect that tended to be more pronounced in female offspring. Furthermore, 5-Htt genotype, PS and their interaction differentially affected the expression of numerous genes and related pathways within the female hippocampus. Whereas MAPK and neurotrophin signaling were regulated by both the 5-Htt +/− genotype and PS exposure, cytokine and Wnt signaling were affected in a 5-Htt genotype×PS manner, indicating a G×E interaction at the molecular level. Thus, the present study indicates that the long-term stress- and depression-related behavioral effects of PS in C57BL6 mice are partly dependent on the 5-Htt genotype. Moreover, our gene expression findings provide evidence for a molecular basis of such a G×E interaction, which eventually might help to identify novel targets for the diagnostic assessment and treatment of disorders of emotion regulation.

## Supporting Information

Text S1 (DOC)

Text S2 (DOC)

Text S3 (DOC)

## References

[1] Weinstock M (2008). The long-term behavioural consequences of prenatal stress.. Neurosci Biobehav Rev.

[2] Pallares ME, Scacchi Bernasconi PA, Feleder C, Cutrera RA (2007). Effects of prenatal stress on motor performance and anxiety behavior in Swiss mice.. Physiol Behav.

[3] Zuena AR, Mairesse J, Casolini P, Cinque C, Alema GS (2008). Prenatal restraint stress generates two distinct behavioral and neurochemical profiles in male and female rats.. PLoS One.

[4] van den Hove DL, Kenis G, Steinbusch HW, Blanco CE, Prickaerts J (2010). Maternal stress-induced reduction in birth weight as a marker for adult affective state.. Front Biosci (Elite Ed).

[5] Weinstock M (2001). Alterations induced by gestational stress in brain morphology and behaviour of the offspring.. Prog Neurobiol.

[6] Huizink AC, Mulder EJ, Buitelaar JK (2004). Prenatal stress and risk for psychopathology: specific effects or induction of general susceptibility?. Psychol Bull.

[7] Caspi A, Hariri AR, Holmes A, Uher R, Moffitt TE (2010). Genetic Sensitivity to the Environment: The Case of the Serotonin Transporter Gene and Its Implications for Studying Complex Diseases and Traits.. Am J Psychiatry.

[8] Lesch KP, Bengel D, Heils A, Sabol SZ, Greenberg BD (1996). Association of anxiety-related traits with a polymorphism in the serotonin transporter gene regulatory region.. Science.

[9] Canli T, Lesch KP (2007). Long story short: the serotonin transporter in emotion regulation and social cognition.. Nat Neurosci.

[10] Kaufman J, Yang BZ, Douglas-Palumberi H, Grasso D, Lipschitz D (2006). Brain-derived neurotrophic factor-5-HTTLPR gene interactions and environmental modifiers of depression in children.. Biol Psychiatry.

[11] Murphy DL, Lesch KP (2008). Targeting the murine serotonin transporter: insights into human neurobiology.. Nat Rev Neurosci.

[12] Holmes A, Yang RJ, Lesch KP, Crawley JN, Murphy DL (2003). Mice lacking the serotonin transporter exhibit 5-HT(1A) receptor-mediated abnormalities in tests for anxiety-like behavior.. Neuropsychopharmacology.

[13] Holmes A, Yang RJ, Murphy DL, Crawley JN (2002). Evaluation of antidepressant-related behavioral responses in mice lacking the serotonin transporter.. Neuropsychopharmacology.

[14] Jansen F, Heiming RS, Lewejohann L, Touma C, Palme R (2010). Modulation of behavioural profile and stress response by 5-HTT genotype and social experience in adulthood.. Behav Brain Res.

[15] Wellman CL, Izquierdo A, Garrett JE, Martin KP, Carroll J (2007). Impaired stress-coping and fear extinction and abnormal corticolimbic morphology in serotonin transporter knock-out mice.. J Neurosci.

[16] Bartolomucci A, Carola V, Pascucci T, Puglisi-Allegra S, Cabib S (2010). Increased vulnerability to psychosocial stress in heterozygous serotonin transporter knockout mice.. Dis Model Mech.

[17] Carroll JC, Boyce-Rustay JM, Millstein R, Yang R, Wiedholz LM (2007). Effects of mild early life stress on abnormal emotion-related behaviors in 5-HTT knockout mice.. Behav Genet.

[18] Heiming RS, Jansen F, Lewejohann L, Kaiser S, Schmitt A (2009). Living in a dangerous world: the shaping of behavioral profile by early environment and 5-HTT genotype.. Front Behav Neurosci.

[19] Carola V, Frazzetto G, Pascucci T, Audero E, Puglisi-Allegra S (2008). Identifying molecular substrates in a mouse model of the serotonin transporter×environment risk factor for anxiety and depression.. Biol Psychiatry.

[20] Caspi A, Sugden K, Moffitt TE, Taylor A, Craig IW (2003). Influence of life stress on depression: moderation by a polymorphism in the 5-HTT gene.. Science.

[21] Bengel D, Murphy DL, Andrews AM, Wichems CH, Feltner D (1998). Altered brain serotonin homeostasis and locomotor insensitivity to 3, 4-methylenedioxymethamphetamine (“Ecstasy”) in serotonin transporter-deficient mice.. Mol Pharmacol.

[22] Behan AT, van den Hove DL, Mueller L, Jetten MJ, Steinbusch HW (2011). Evidence of female-specific glial deficits in the hippocampus in a mouse model of prenatal stress.. Eur Neuropsychopharmacol.

[23] Chapman RH, Stern JM (1979). Failure of severe maternal stress or ACTH during pregnancy to affect emotionality of male rat offspring: implications of litter effects for prenatal studies.. Dev Psychobiol.

[24] Sik A, van Nieuwehuyzen P, Prickaerts J, Blokland A (2003). Performance of different mouse strains in an object recognition task.. Behavioural Brain Research.

[25] Shepherd JK, Grewal SS, Fletcher A, Bill DJ, Dourish CT (1994). Behavioural and pharmacological characterisation of the elevated “zero-maze” as an animal model of anxiety.. Psychopharmacology (Berl).

[26] van Donkelaar A, Martin RV, Brauer M, Kahn R, Levy R (2010). Global estimates of ambient fine particulate matter concentrations from satellite-based aerosol optical depth: development and application.. Environ Health Perspect.

[27] Borsini F, Meli A (1988). Is the forced swimming test a suitable model for revealing antidepressant activity?. Psychopharmacology (Berl).

[28] Van den Hove DL, Steinbusch HW, Scheepens A, Van de Berg WD, Kooiman LA (2006). Prenatal stress and neonatal rat brain development.. Neuroscience.

[29] Huber W, von Heydebreck A, Sultmann H, Poustka A, Vingron M (2002). Variance stabilization applied to microarray data calibration and to the quantification of differential expression.. Bioinformatics.

[30] Dennis G, Sherman BT, Hosack DA, Yang J, Gao W (2003). DAVID: Database for Annotation, Visualization, and Integrated Discovery.. Genome Biol.

[31] Huang da W, Sherman BT, Lempicki RA (2009). Systematic and integrative analysis of large gene lists using DAVID bioinformatics resources.. Nat Protoc.

[32] Ruijter JM, Ramakers C, Hoogaars WM, Karlen Y, Bakker O (2009). Amplification efficiency: linking baseline and bias in the analysis of quantitative PCR data.. Nucleic Acids Res.

[33] Vandesompele J, De Preter K, Pattyn F, Poppe B, Van Roy N (2002). Accurate normalization of real-time quantitative RT-PCR data by geometric averaging of multiple internal control genes.. Genome Biol.

[34] Homberg JR, Lesch KP (2011). Looking on the bright side of serotonin transporter gene variation.. Biol Psychiatry.

[35] Smyth GK, Speed T (2003). Normalization of cDNA microarray data.. Methods.

[36] Smyth GK, Michaud J, Scott HS (2005). Use of within-array replicate spots for assessing differential expression in microarray experiments.. Bioinformatics.

[37] Fanselow MS, Dong HW (2010). Are the dorsal and ventral hippocampus functionally distinct structures?. Neuron.

[38] Ravary A, Muzerelle A, Darmon M, Murphy DL, Moessner R (2001). Abnormal trafficking and subcellular localization of an N-terminally truncated serotonin transporter protein.. Eur J Neurosci.

[39] Olivier JD, Jans LA, Korte-Bouws GA, Korte SM, Deen PM (2008). Acute tryptophan depletion dose dependently impairs object memory in serotonin transporter knockout rats.. Psychopharmacology (Berl).

[40] Roiser JP, Muller U, Clark L, Sahakian BJ (2007). The effects of acute tryptophan depletion and serotonin transporter polymorphism on emotional processing in memory and attention.. International Journal of Neuropsychopharmacology.

[41] Vanmierlo T, Rutten K, Dederen J, Bloks VW, van Vark-van der Zee LC (2009). Liver X receptor activation restores memory in aged AD mice without reducing amyloid.. Neurobiol Aging.

[42] Jones KL, Smith RM, Edwards KS, Givens B, Tilley MR (2010). Combined effect of maternal serotonin transporter genotype and prenatal stress in modulating offspring social interaction in mice.. Int J Dev Neurosci.

[43] Wust S, Kumsta R, Treutlein J, Frank J, Entringer S (2009). Sex-specific association between the 5-HTT gene-linked polymorphic region and basal cortisol secretion.. Psychoneuroendocrinology.

[44] Keshet Y, Seger R (2010). The MAP kinase signaling cascades: a system of hundreds of components regulates a diverse array of physiological functions.. Methods Mol Biol.

[45] Asaoka Y, Nishina H (2010). Diverse physiological functions of MKK4 and MKK7 during early embryogenesis.. J Biochem.

[46] Li H, Zhang L, Huang Q (2009). Differential expression of mitogen-activated protein kinase signaling pathway in the hippocampus of rats exposed to chronic unpredictable stress.. Behav Brain Res.

[47] Pearson G, Robinson F, Beers Gibson T, Xu BE, Karandikar M (2001). Mitogen-activated protein (MAP) kinase pathways: regulation and physiological functions.. Endocr Rev.

[48] Liu YF, Bertram K, Perides G, McEwen BS, Wang D (2004). Stress induces activation of stress-activated kinases in the mouse brain.. J Neurochem.

[49] Meller E, Shen C, Nikolao TA, Jensen C, Tsimberg Y (2003). Region-specific effects of acute and repeated restraint stress on the phosphorylation of mitogen-activated protein kinases.. Brain Res.

[50] Turner CA, Akil H, Watson SJ, Evans SJ (2006). The fibroblast growth factor system and mood disorders.. Biological Psychiatry.

[51] Gaughran F, Payne J, Sedgwick PM, Cotter D, Berry M (2006). Hippocampal FGF-2 and FGFR1 mRNA expression in major depression, schizophrenia and bipolar disorder.. Brain Res Bull.

[52] Duman RS, Malberg J, Nakagawa S, D'Sa C (2000). Neuronal plasticity and survival in mood disorders.. Biol Psychiatry.

[53] Jacobs BL, van Praag H, Gage FH (2000). Adult brain neurogenesis and psychiatry: a novel theory of depression.. Mol Psychiatry.

[54] Lu B, Pang PT, Woo NH (2005). The yin and yang of neurotrophin action.. Nat Rev Neurosci.

[55] Reichardt LF (2006). Neurotrophin-regulated signalling pathways.. Philos Trans R Soc Lond B Biol Sci.

[56] Barco A, Alarcon JM, Kandel ER (2002). Expression of constitutively active CREB protein facilitates the late phase of long-term potentiation by enhancing synaptic capture.. Cell.

[57] Taliaz D, Stall N, Dar DE, Zangen A (2010). Knockdown of brain-derived neurotrophic factor in specific brain sites precipitates behaviors associated with depression and reduces neurogenesis.. Molecular Psychiatry.

[58] Martinowich K, Manji H, Lu B (2007). New insights into BDNF function in depression and anxiety.. Nat Neurosci.

[59] Woo NH, Teng HK, Siao CJ, Chiaruttini C, Pang PT (2005). Activation of p75NTR by proBDNF facilitates hippocampal long-term depression.. Nat Neurosci.

[60] Xu L, Anwyl R, Rowan MJ (1997). Behavioural stress facilitates the induction of long-term depression in the hippocampus.. Nature.

[61] Holderbach R, Clark K, Moreau JL, Bischofberger J, Normann C (2007). Enhanced long-term synaptic depression in an animal model of depression.. Biol Psychiatry.

[62] Fredericks CA, Drabant EM, Edge MD, Tillie JM, Hallmayer J (2010). Healthy young women with serotonin transporter SS polymorphism show a pro-inflammatory bias under resting and stress conditions.. Brain Behav Immun.

[63] Lotrich FE, Ferrell RE, Rabinovitz M, Pollock BG (2009). Risk for depression during interferon-alpha treatment is affected by the serotonin transporter polymorphism.. Biol Psychiatry.

[64] Tsao CW, Lin YS, Chen CC, Bai CH, Wu SR (2006). Cytokines and serotonin transporter in patients with major depression.. Prog Neuropsychopharmacol Biol Psychiatry.

[65] Dantzer R, O'Connor JC, Freund GG, Johnson RW, Kelley KW (2008). From inflammation to sickness and depression: when the immune system subjugates the brain.. Nat Rev Neurosci.

[66] Cizza G, Marques AH, Eskandari F, Christie IC, Torvik S (2008). Elevated Neuroimmune Biomarkers in Sweat Patches and Plasma of Premenopausal Women with Major Depressive Disorder in Remission: The POWER Study.. Biological Psychiatry.

[67] Capuron L, Su S, Miller AH, Bremner JD, Goldberg J (2008). Depressive symptoms and metabolic syndrome: is inflammation the underlying link?. Biol Psychiatry.

[68] Kenis G, Maes M (2002). Effects of antidepressants on the production of cytokines.. International Journal of Neuropsychopharmacology.

[69] Reichenberg A, Yirmiya R, Schuld A, Kraus T, Haack M (2001). Cytokine-associated emotional and cognitive disturbances in humans.. Archives of General Psychiatry.

[70] Capuron L, Ravaud A, Dantzer R (2000). Early depressive symptoms in cancer patients receiving interleukin 2 and/or interferon alfa-2b therapy.. Journal of Clinical Oncology.

[71] Miller AH, Maletic V, Raison CL (2009). Inflammation and its discontents: the role of cytokines in the pathophysiology of major depression.. Biol Psychiatry.

[72] Raison CL, Borisov AS, Majer M, Drake DF, Pagnoni G (2009). Activation of Central Nervous System Inflammatory Pathways by Interferon-Alpha: Relationship to Monoamines and Depression.. Biological Psychiatry.

[73] Capuron L, Gumnick JF, Musselman DL, Lawson DH, Reemsnyder A (2002). Neurobehavioral effects of interferon-alpha in cancer patients: Phenomenology and paroxetine responsiveness of symptom dimensions.. Neuropsychopharmacology.

[74] Kenis G, Prickaerts J, van Os J, Koek GH, Robaeys G (2010). Depressive symptoms following interferon-alpha therapy: mediated by immune-induced reductions in brain-derived neurotrophic factor?. Int J Neuropsychopharmacol.

[75] Leonard BE, Myint A (2009). The psychoneuroimmunology of depression.. Human Psychopharmacology-Clinical and Experimental.

[76] Robinson CM, Hale PT, Carlin JM (2005). The role of IFN-gamma and TNF-alpha-responsive regulatory elements in the synergistic induction of indoleamine dioxygenase.. J Interferon Cytokine Res.

[77] Schiepers OJ, Wichers MC, Maes M (2005). Cytokines and major depression.. Prog Neuropsychopharmacol Biol Psychiatry.

[78] Wu X, Hsuchou H, Kastin AJ, He Y, Khan RS (2011). Interleukin-15 affects serotonin system and exerts antidepressive effects through IL15Ralpha receptor.. Psychoneuroendocrinology.

[79] Wu X, He Y, Hsuchou H, Kastin AJ, Rood JC (2010). Essential role of interleukin-15 receptor in normal anxiety behavior.. Brain Behav Immun.

[80] Staal FJ, Luis TC, Tiemessen MM (2008). WNT signalling in the immune system: WNT is spreading its wings.. Nat Rev Immunol.

[81] Gould TD, O'Donnell KC, Picchini AM, Dow ER, Chen G (2008). Generation and behavioral characterization of beta-catenin forebrain-specific conditional knock-out mice.. Behavioural Brain Research.

[82] Gould TD, Zarate CA, Manji HK (2004). Glycogen synthase kinase-3: a target for novel bipolar disorder treatments.. J Clin Psychiatry.

[83] Emamian ES, Hall D, Birnbaum MJ, Karayiorgou M, Gogos JA (2004). Convergent evidence for impaired AKT1-GSK3beta signaling in schizophrenia.. Nat Genet.

[84] Wada A (2009). Lithium and neuropsychiatric therapeutics: neuroplasticity via glycogen synthase kinase-3beta, beta-catenin, and neurotrophin cascades.. J Pharmacol Sci.

[85] Chen Q, Schubert D (2002). Presenilin-interacting proteins.. Expert Rev Mol Med.

[86] Arora V, Cheung HH, Plenchette S, Micali OC, Liston P (2007). Degradation of survivin by the x-linked inhibitor of apoptosis (XIAP)-XAF1 complex.. Journal of Biological Chemistry.

[87] Kairisalo M, Korhonen L, Sepp M, Pruunsild P, Kukkonen JP (2009). NF-kappaB-dependent regulation of brain-derived neurotrophic factor in hippocampal neurons by X-linked inhibitor of apoptosis protein.. Eur J Neurosci.

[88] Persico AM, Baldi A, Dell'Acqua ML, Moessner R, Murphy DL (2003). Reduced programmed cell death in brains of serotonin transporter knockout mice.. Neuroreport.

[89] Martins-de-Souza D, Maccarrone G, Wobrock T, Zerr I, Gormanns P (2010). Proteome analysis of the thalamus and cerebrospinal fluid reveals glycolysis dysfunction and potential biomarkers candidates for schizophrenia.. J Psychiatr Res.

[90] Zeng H, Schimpf BA, Rohde AD, Pavlova MN, Gragerov A (2007). Thyrotropin-releasing hormone receptor 1-deficient mice display increased depression and anxiety-like behavior.. Mol Endocrinol.

[91] Patin V, Lordi B, Vincent A, Thoumas JL, Vaudry H (2002). Effects of prenatal stress on maternal behavior in the rat.. Brain Res Dev Brain Res.

