# Correction: Differential Effects of Prenatal Stress in 5-Htt Deficient Mice: Towards Molecular Mechanisms of Gene × Environment Interactions

**DOI:** 10.1371/annotation/8f24480c-d341-48e3-8bb2-5e0275941f87

**Published:** 2011-10-05

**Authors:** Daniel Louis Albert van den Hove, Sissi Brigitte Jakob, Karla-Gerlinde Schraut, Gunter Kenis, Angelika Gertrud Schmitt, Susanne Kneitz, Claus-Jürgen Scholz, Valentina Wiescholleck, Gabriela Ortega, Jos Prickaerts, Harry Steinbusch, Klaus-Peter Lesch

The correct first author name is: Daniel Louis Albert van den Hove

The corrected citation is: van den Hove DLA, Jakob SB, Schraut K-G, Kenis G, Schmitt AG, et al. (2011) Differential Effects of Prenatal Stress in 5-Htt Deficient Mice: Towards Molecular Mechanisms of Gene × Environment Interactions. PLoS ONE 6(8): e22715. doi:10.1371/journal.pone.0022715

