# Supplementary material for: Differential Effects of Prenatal Stress in 5-Htt Deficient Mice: Towards Molecular Mechanisms of Gene × Environment Interactions
Source: PLoS One. 2011 Aug 12;6(8):e22715. doi: 10.1371/journal.pone.0022715 (PMC3155516; doi:10.1371/journal.pone.0022715)
Supplement: Text S1 — (DOC) [file pone.0022715.s001.doc]

**Supplemental material 1**

**Genes affected by the genotype (5-Htt +/- versus wild type)**.

ID: Affymetrix ID; EFFECT: direction of effect; “+”: gene expression upregulated in 5-Htt +/- versus wild type mice; “-“: downregulated in 5-Htt +/- versus wild type mice; FC: fold-change in mRNA expression. Genes have been ordered alphabetically by symbol.

| **#** | **ID** | **SYMBOL** | **GENENAME** | **EFFECT** | **FC** | **P-Value** |
| --- | --- | --- | --- | --- | --- | --- |
| 1 | 1432532_at | 0610025J13Rik | RIKEN cDNA 0610025J13 gene | + | 1.3 | 0.0001 |
| 2 | 1431884_at | 1110019B22Rik | RIKEN cDNA 1110019B22 gene | + | 1.2 | 0.0078 |
| 3 | 1439363_at | 1200014J11Rik | RIKEN cDNA 1200014J11 gene | - | 1.6 | 0.0000 |
| 4 | 1451082_at | 1300018I05Rik | RIKEN cDNA 1300018I05 gene | - | 1.2 | 0.0037 |
| 5 | 1429091_at | 1600002K03Rik | RIKEN cDNA 1600002K03 gene | + | 1.2 | 0.0081 |
| 6 | 1429324_at | 1700012A16Rik | RIKEN cDNA 1700012A16 gene | + | 1.2 | 0.0097 |
| 7 | 1432983_at | 1700026H06Rik | RIKEN cDNA 1700026H06 gene | + | 1.2 | 0.0089 |
| 8 | 1430513_at | 1700055N04Rik | RIKEN cDNA 1700055N04 gene | + | 1.4 | 0.0024 |
| 9 | 1429198_at | 1810030O07Rik | RIKEN cDNA 1810030O07 gene | - | 1.3 | 0.0043 |
| 10 | 1441251_a_at | 2010001A14Rik | RIKEN cDNA 2010001A14 gene | + | 1.3 | 0.0008 |
| 11 | 1439561_at | 2010012O05Rik | RIKEN cDNA 2010012O05 gene | - | 1.2 | 0.0039 |
| 12 | 1460478_at | 2200002J24Rik | RIKEN cDNA 2200002J24 gene | + | 1.2 | 0.0049 |
| 13 | 1437145_s_at | 2310002J15Rik | RIKEN cDNA 2310002J15 gene | + | 1.3 | 0.0095 |
| 14 | 1429844_at | 2310043J07Rik | RIKEN cDNA 2310043J07 gene | + | 1.2 | 0.0075 |
| 15 | 1427922_at | 2310061C15Rik | RIKEN cDNA 2310061C15 gene | + | 1.2 | 0.0062 |
| 16 | 1432650_at | 2510019K15Rik | RIKEN cDNA 2510019K15 gene | + | 1.2 | 0.0086 |
| 17 | 1451078_at | 2510039O18Rik | RIKEN cDNA 2510039O18 gene | - | 1.2 | 0.0026 |
| 18 | 1431679_at | 2510042H12Rik | RIKEN cDNA 2510042H12 gene | + | 1.4 | 0.0046 |
| 19 | 1430876_at | 2610312I10Rik | RIKEN cDNA 2610312I10 gene | + | 1.9 | 0.0039 |
| 20 | 1424984_at | 2700078E11Rik | RIKEN cDNA 2700078E11 gene | - | 1.3 | 0.0045 |
| 21 | 1430178_at | 2810408A11Rik | RIKEN cDNA 2810408A11 gene | + | 1.4 | 0.0023 |
| 22 | 1433387_at | 2900022M07Rik | RIKEN cDNA 2900022M07 gene | + | 1.4 | 0.0011 |
| 23 | 1432861_at | 2900046F13Rik | RIKEN cDNA 2900046F13 gene | + | 1.3 | 0.0033 |
| 24 | 1432995_at | 2900057E15Rik | RIKEN cDNA 2900057E15 gene | + | 1.3 | 0.0099 |
| 25 | 1432797_at | 2900060N12Rik | RIKEN cDNA 2900060N12 gene | + | 1.3 | 0.0036 |
| 26 | 1458869_at | 2900076A13Rik | RIKEN cDNA 2900076A13 gene | + | 1.2 | 0.0057 |
| 27 | 1430162_at | 3830417A13Rik | RIKEN cDNA 3830417A13 gene | - | 1.2 | 0.0077 |
| 28 | 1454439_at | 4833419G08Rik | RIKEN cDNA 4833419G08 gene | + | 1.2 | 0.0055 |
| 29 | 1419636_at | 4833420G17Rik | RIKEN cDNA 4833420G17 gene | - | 1.2 | 0.0063 |
| 30 | 1419635_at | 4833420G17Rik | RIKEN cDNA 4833420G17 gene | - | 1.3 | 0.0072 |
| 31 | 1459879_at | 4921513D23Rik | RIKEN cDNA 4921513D23 gene | + | 1.2 | 0.0056 |
| 32 | 1430732_at | 4921525D07Rik | RIKEN cDNA 4921525D07 gene | + | 1.3 | 0.0096 |
| 33 | 1432123_at | 4930455H04Rik | RIKEN cDNA 4930455H04 gene | - | 1.2 | 0.0033 |
| 34 | 1453549_at | 4930500G05Rik | RIKEN cDNA 4930500G05 gene | + | 1.3 | 0.0042 |
| 35 | 1439133_at | 4930524O07Rik | RIKEN cDNA 4930524O07 gene | + | 1.4 | 0.0014 |
| 36 | 1432364_at | 4930556N13Rik | RIKEN cDNA 4930556N13 gene | + | 1.2 | 0.0061 |
| 37 | 1418870_at | 4930579J09Rik | RIKEN cDNA 4930579J09 gene | - | 1.4 | 0.0032 |
| 38 | 1433056_at | 4931407J08Rik | RIKEN cDNA 4931407J08 gene | + | 1.2 | 0.0097 |
| 39 | 1432882_at | 4932431P20Rik | RIKEN cDNA 4932431P20 gene | + | 1.5 | 0.0032 |
| 40 | 1453652_at | 4933400F21Rik | RIKEN cDNA 4933400F21 gene | + | 1.3 | 0.0039 |
| 41 | 1454539_at | 4933417G07Rik | RIKEN cDNA 4933417G07 gene | + | 1.3 | 0.0002 |
| 42 | 1453894_at | 4933434C23Rik | RIKEN cDNA 4933434C23 gene | + | 1.2 | 0.0037 |
| 43 | 1452696_a_at | 4933439C10Rik | RIKEN cDNA 4933439C10 gene | - | 1.2 | 0.0080 |
| 44 | 1431521_at | 5033428B15Rik | RIKEN cDNA 5033428B15 gene | - | 1.3 | 0.0025 |
| 45 | 1428220_at | 5730419I09Rik | RIKEN cDNA 5730419I09 gene | + | 1.2 | 0.0079 |
| 46 | 1432696_at | 5830431M20Rik | RIKEN cDNA 5830431M20 gene | + | 1.2 | 0.0085 |
| 47 | 1432625_at | 5830487K18Rik | RIKEN cDNA 5830487K18 gene | + | 1.4 | 0.0016 |
| 48 | 1440849_at | 6330417G04Rik | RIKEN cDNA 6330417G04 gene | + | 1.2 | 0.0066 |
| 49 | 1441725_at | 6530402F18Rik | RIKEN cDNA 6530402F18 gene | + | 1.3 | 0.0013 |
| 50 | 1429731_at | 6530403G13Rik | RIKEN cDNA 6530403G13 gene | - | 1.2 | 0.0091 |
| 51 | 1423071_x_at | 6720475J19Rik | RIKEN cDNA 6720475J19 gene | + | 1.3 | 0.0009 |
| 52 | 1429620_at | 8430406I07Rik | RIKEN cDNA 8430406I07 gene | + | 1.3 | 0.0080 |
| 53 | 1430438_at | 8430439j12rik | RIKEN cDNA 8430439J12 gene | + | 1.3 | 0.0034 |
| 54 | 1453638_at | 9030420J04Rik | RIKEN cDNA 9030420J04 gene | + | 1.2 | 0.0055 |
| 55 | 1430103_at | 9030607L20Rik | RIKEN cDNA 9030607L20 gene | + | 1.2 | 0.0009 |
| 56 | 1454361_at | 9230106L01Rik | RIKEN cDNA 9230106L01 gene | + | 1.2 | 0.0057 |
| 57 | 1457671_at | 9330120H11Rik | RIKEN cDNA 9330120H11 gene | + | 1.2 | 0.0071 |
| 58 | 1454588_at | 9430006E15Rik | RIKEN cDNA 9430006E15 gene | + | 1.4 | 0.0089 |
| 59 | 1457161_at | 9530029O12Rik | RIKEN cDNA 9530029O12 gene | + | 1.3 | 0.0012 |
| 60 | 1433289_at | 9530078B04Rik | RIKEN cDNA 9530078B04 gene | + | 1.3 | 0.0008 |
| 61 | 1440884_s_at | A530047J11Rik | RIKEN cDNA A530047J11 gene | + | 1.3 | 0.0046 |
| 62 | 1440413_at | A830006F12Rik | RIKEN cDNA A830006F12 gene | + | 1.4 | 0.0091 |
| 63 | 1445721_at | A830021M18 | hypothetical protein A830021M18 | + | 1.3 | 0.0036 |
| 64 | 1442844_at | A830052D11Rik | RIKEN cDNA A830052D11 gene | + | 1.2 | 0.0033 |
| 65 | 1419797_at | AA672641 | expressed sequence AA672641 | + | 1.2 | 0.0093 |
| 66 | 1430394_a_at | Abcb9 | ATP-binding cassette, sub-family B (MDR/TAP), member 9 | - | 1.3 | 0.0039 |
| 67 | 1421212_at | Abcc6 | ATP-binding cassette, sub-family C (CFTR/MRP), member 6 | - | 1.3 | 0.0036 |
| 68 | 1450141_at | Abcg3 | ATP-binding cassette, sub-family G (WHITE), member 3 | + | 1.3 | 0.0007 |
| 69 | 1447548_at | Abhd5 | abhydrolase domain containing 5 | - | 1.3 | 0.0027 |
| 70 | 1422635_at | Ache | acetylcholinesterase | - | 1.3 | 0.0004 |
| 71 | 1418677_at | Actn3 | actinin alpha 3 | + | 1.2 | 0.0036 |
| 72 | 1419173_at | Acy1 | aminoacylase 1 | - | 1.3 | 0.0040 |
| 73 | 1450801_at | Adam21 | a disintegrin and metallopeptidase domain 21 | + | 1.2 | 0.0075 |
| 74 | 1424798_a_at | Adam5 | a disintegrin and metallopeptidase domain 5 | + | 1.2 | 0.0069 |
| 75 | 1427139_at | Adamts10 | a disintegrin-like and metallopeptidase (reprolysin type) with thrombospondin type 1 motif, 10 | - | 1.3 | 0.0025 |
| 76 | 1446666_at | Adamts12 | a disintegrin-like and metallopeptidase (reprolysin type) with thrombospondin type 1 motif, 12 | - | 1.2 | 0.0096 |
| 77 | 1455965_at | Adamts4 | a disintegrin-like and metallopeptidase (reprolysin type) with thrombospondin type 1 motif, 4 | - | 1.2 | 0.0063 |
| 78 | 1420953_at | Add1 | adducin 1 (alpha) | - | 1.2 | 0.0061 |
| 79 | 1420954_a_at | Add1 | adducin 1 (alpha) | - | 1.3 | 0.0064 |
| 80 | 1416225_at | Adh1 | alcohol dehydrogenase 1 (class I) | - | 1.4 | 0.0006 |
| 81 | 1446675_at | Adk | adenosine kinase | + | 1.3 | 0.0027 |
| 82 | 1443847_x_at | Aff2 | AF4/FMR2 family, member 2 | + | 1.2 | 0.0047 |
| 83 | 1422116_at | Aff2 | AF4/FMR2 family, member 2 | - | 1.2 | 0.0085 |
| 84 | 1431722_a_at | Afmid | arylformamidase | + | 1.3 | 0.0009 |
| 85 | 1420428_at | Ager | advanced glycosylation end product-specific receptor | + | 1.2 | 0.0047 |
| 86 | 1421024_at | Agpat1 | 1-acylglycerol-3-phosphate O-acyltransferase 1 (lysophosphatidic acid acyltransferase, alpha) | - | 1.3 | 0.0033 |
| 87 | 1445647_at | AI447881 | expressed sequence AI447881 | + | 1.2 | 0.0051 |
| 88 | 1438270_at | AI846148 | expressed sequence AI846148 | - | 1.3 | 0.0045 |
| 89 | 1443565_at | AI849538 | expressed sequence AI849538 | + | 1.2 | 0.0097 |
| 90 | 1418279_a_at | Akap1 | A kinase (PRKA) anchor protein 1 | - | 1.2 | 0.0092 |
| 91 | 1452684_at | Akt1s1 | AKT1 substrate 1 (proline-rich) | - | 1.2 | 0.0056 |
| 92 | 1425376_at | Alox8 | arachidonate 8-lipoxygenase | - | 1.3 | 0.0024 |
| 93 | 1449417_at | Ambn | ameloblastin | - | 1.2 | 0.0066 |
| 94 | 1431121_at | Amotl1 | angiomotin-like 1 | - | 1.2 | 0.0043 |
| 95 | 1453307_a_at | Anapc5 | anaphase-promoting complex subunit 5 | - | 1.4 | 0.0003 |
| 96 | 1450085_at | Angptl2 | angiopoietin-like 2 | - | 1.2 | 0.0043 |
| 97 | 1437895_at | Ano8 | anoctamin 8 | - | 1.2 | 0.0047 |
| 98 | 1429246_a_at | Anxa6 | annexin A6 | - | 1.3 | 0.0083 |
| 99 | 1415818_at | Anxa6 | annexin A6 | - | 1.3 | 0.0009 |
| 100 | 1452490_a_at | Ap2a2 | adaptor protein complex AP-2, alpha 2 subunit | - | 1.4 | 0.0007 |
| 101 | 1418383_at | Apcdd1 | adenomatosis polyposis coli down-regulated 1 | - | 1.3 | 0.0081 |
| 102 | 1416134_at | Aplp1 | amyloid beta (A4) precursor-like protein 1 | - | 1.5 | 0.0034 |
| 103 | 1421889_a_at | Aplp2 | amyloid beta (A4) precursor-like protein 2 | - | 1.2 | 0.0015 |
| 104 | 1417561_at | Apoc1 | apolipoprotein C-I | - | 1.3 | 0.0058 |
| 105 | 1446580_at | Apool | apolipoprotein O-like | + | 1.2 | 0.0061 |
| 106 | 1419833_s_at | Arap3 | ArfGAP with RhoGAP domain, ankyrin repeat and PH domain 3 | - | 1.2 | 0.0088 |
| 107 | 1423743_at | Arcn1 | archain 1 | - | 1.3 | 0.0003 |
| 108 | 1438661_a_at | Arf2 | ADP-ribosylation factor 2 | - | 1.4 | 0.0019 |
| 109 | 1457219_at | Arhgef18 | rho/rac guanine nucleotide exchange factor (GEF) 18 | - | 1.4 | 0.0032 |
| 110 | 1429859_a_at | Arl2bp | ADP-ribosylation factor-like 2 binding protein | - | 1.2 | 0.0083 |
| 111 | 1451131_at | Arl6ip1 | ADP-ribosylation factor-like 6 interacting protein 1 | - | 1.2 | 0.0092 |
| 112 | 1429816_at | Armc3 | armadillo repeat containing 3 | - | 1.2 | 0.0084 |
| 113 | 1427002_s_at | Arsg | arylsulfatase G | - | 1.3 | 0.0044 |
| 114 | 1427759_a_at | Art5 | ADP-ribosyltransferase 5 | + | 1.3 | 0.0030 |
| 115 | 1424225_at | Asb8 | ankyrin repeat and SOCS box-containing 8 | - | 1.3 | 0.0062 |
| 116 | 1460703_at | Ascc1 | activating signal cointegrator 1 complex subunit 1 | - | 1.3 | 0.0059 |
| 117 | 1418292_at | Asna1 | arsA arsenite transporter, ATP-binding, homolog 1 (bacterial) | - | 1.4 | 0.0033 |
| 118 | 1420036_at | Ate1 | arginyltransferase 1 | + | 1.3 | 0.0026 |
| 119 | 1416794_at | Atl2 | atlastin GTPase 2 | - | 1.3 | 0.0017 |
| 120 | 1427481_a_at | Atp1a3 | ATPase, Na+/K+ transporting, alpha 3 polypeptide | - | 1.3 | 0.0088 |
| 121 | 1459924_at | Atp6v0a1 | ATPase, H+ transporting, lysosomal V0 subunit A1 | + | 1.3 | 0.0051 |
| 122 | 1416769_s_at | Atp6v0b | ATPase, H+ transporting, lysosomal V0 subunit B | - | 1.4 | 0.0077 |
| 123 | 1444355_at | Atp8a1 | ATPase, aminophospholipid transporter (APLT), class I, type 8A, member 1 | + | 1.3 | 0.0046 |
| 124 | 1421166_at | Atrn | attractin | + | 1.2 | 0.0071 |
| 125 | 1425656_a_at | Baiap2 | brain-specific angiogenesis inhibitor 1-associated protein 2 | - | 1.3 | 0.0008 |
| 126 | 1440645_at | BB114814 | expressed sequence BB114814 | + | 1.3 | 0.0073 |
| 127 | 1427513_at | BC024137 | cDNA sequence BC024137 | + | 1.3 | 0.0019 |
| 128 | 1437122_at | Bcl2 | B-cell leukemia/lymphoma 2 | + | 1.2 | 0.0077 |
| 129 | 1456006_at | Bcl2l11 | BCL2-like 11 (apoptosis facilitator) | + | 1.4 | 0.0049 |
| 130 | 1425532_a_at | Bin1 | bridging integrator 1 | - | 1.2 | 0.0093 |
| 131 | 1451870_a_at | Brd4 | bromodomain containing 4 | - | 1.3 | 0.0013 |
| 132 | 1456616_a_at | Bsg | basigin | - | 1.4 | 0.0010 |
| 133 | 1444666_at | Bxdc1 | brix domain containing 1 | + | 1.5 | 0.0000 |
| 134 | 1451085_at | C030006K11Rik | RIKEN cDNA C030006K11 gene | - | 1.3 | 0.0095 |
| 135 | 1433932_x_at | C030046I01Rik | RIKEN cDNA C030046I01 gene | - | 1.2 | 0.0039 |
| 136 | 1433931_at | C030046I01Rik | RIKEN cDNA C030046I01 gene | - | 1.3 | 0.0043 |
| 137 | 1422772_at | C1galt1 | core 1 synthase, glycoprotein-N-acetylgalactosamine 3-beta-galactosyltransferase, 1 | + | 1.3 | 0.0017 |
| 138 | 1449401_at | C1qc | complement component 1, q subcomponent, C chain | - | 1.2 | 0.0070 |
| 139 | 1449308_at | C6 | complement component 6 | - | 1.2 | 0.0084 |
| 140 | 1419969_at | C77370 | expressed sequence C77370 | + | 1.3 | 0.0047 |
| 141 | 1442540_at | C77609 | expressed sequence C77609 | + | 1.3 | 0.0065 |
| 142 | 1441401_at | C79329 | expressed sequence C79329 | + | 1.6 | 0.0003 |
| 143 | 1421255_a_at | Cabp1 | calcium binding protein 1 | - | 1.3 | 0.0045 |
| 144 | 1425963_at | Cabp7 | calcium binding protein 7 | - | 1.3 | 0.0065 |
| 145 | 1425812_a_at | Cacna1b | calcium channel, voltage-dependent, N type, alpha 1B subunit | - | 1.4 | 0.0043 |
| 146 | 1421297_a_at | Cacna1c | calcium channel, voltage-dependent, L type, alpha 1C subunit | - | 1.2 | 0.0083 |
| 147 | 1428051_a_at | Cacna1d | calcium channel, voltage-dependent, L type, alpha 1D subunit | - | 1.2 | 0.0035 |
| 148 | 1450520_at | Cacng3 | calcium channel, voltage-dependent, gamma subunit 3 | - | 1.3 | 0.0014 |
| 149 | 1453455_at | Camta1 | calmodulin binding transcription activator 1 | + | 1.2 | 0.0085 |
| 150 | 1417462_at | Cap1 | CAP, adenylate cyclase-associated protein 1 (yeast) | - | 1.2 | 0.0029 |
| 151 | 1456848_at | Ccdc123 | coiled-coil domain containing 123 | - | 1.2 | 0.0077 |
| 152 | 1457628_at | Ccdc53 | coiled-coil domain containing 53 | + | 1.4 | 0.0071 |
| 153 | 1430670_at | Ccdc91 | coiled-coil domain containing 91 | + | 1.3 | 0.0047 |
| 154 | 1418459_at | Ccdc91 | coiled-coil domain containing 91 | - | 1.2 | 0.0034 |
| 155 | 1444323_at | Ccnd3 | cyclin D3 | + | 1.2 | 0.0091 |
| 156 | 1415907_at | Ccnd3 | cyclin D3 | - | 1.2 | 0.0060 |
| 157 | 1433741_at | Cd38 | CD38 antigen | - | 1.2 | 0.0085 |
| 158 | 1443976_at | Cdk5rap2 | CDK5 regulatory subunit associated protein 2 | - | 1.3 | 0.0031 |
| 159 | 1419497_at | Cdkn1b | cyclin-dependent kinase inhibitor 1B | - | 1.4 | 0.0003 |
| 160 | 1454891_at | Cds2 | CDP-diacylglycerol synthase (phosphatidate cytidylyltransferase) 2 | - | 1.2 | 0.0087 |
| 161 | 1434533_x_at | Ceacam11 | carcinoembryonic antigen-related cell adhesion molecule 11 | - | 1.2 | 0.0063 |
| 162 | 1418982_at | Cebpa | CCAAT/enhancer binding protein (C/EBP), alpha | - | 1.2 | 0.0088 |
| 163 | 1427844_a_at | Cebpb | CCAAT/enhancer binding protein (C/EBP), beta | - | 1.2 | 0.0039 |
| 164 | 1452242_at | Cep55 | centrosomal protein 55 | - | 1.2 | 0.0072 |
| 165 | 1448052_at | Cgnl1 | cingulin-like 1 | - | 1.2 | 0.0083 |
| 166 | 1451295_a_at | Chd4 | chromodomain helicase DNA binding protein 4 | - | 1.2 | 0.0096 |
| 167 | 1425450_at | Chi3l4 | chitinase 3-like 4 | + | 1.4 | 0.0044 |
| 168 | 1453844_at | Chit1 | chitinase 1 (chitotriosidase) | + | 1.3 | 0.0082 |
| 169 | 1456722_at | Chrdl1 | chordin-like 1 | + | 1.2 | 0.0057 |
| 170 | 1426866_at | Chst14 | carbohydrate (N-acetylgalactosamine 4-0) sulfotransferase 14 | + | 1.2 | 0.0030 |
| 171 | 1448140_at | Ciapin1 | cytokine induced apoptosis inhibitor 1 | - | 1.2 | 0.0037 |
| 172 | 1450014_at | Cldn1 | claudin 1 | + | 1.2 | 0.0036 |
| 173 | 1416003_at | Cldn11 | claudin 11 | - | 1.3 | 0.0088 |
| 174 | 1429574_at | Clic3 | chloride intracellular channel 3 | - | 1.3 | 0.0006 |
| 175 | 1431098_at | Clip1 | CAP-GLY domain containing linker protein 1 | - | 1.2 | 0.0089 |
| 176 | 1425321_a_at | Clmn | calmin | - | 1.2 | 0.0039 |
| 177 | 1416883_at | Clptm1 | cleft lip and palate associated transmembrane protein 1 | - | 1.3 | 0.0012 |
| 178 | 1430519_a_at | Cnot7 | CCR4-NOT transcription complex, subunit 7 | - | 1.4 | 0.0006 |
| 179 | 1449296_a_at | Cnp | 2',3'-cyclic nucleotide 3' phosphodiesterase | - | 1.3 | 0.0033 |
| 180 | 1435166_at | Cntn2 | contactin 2 | - | 1.3 | 0.0034 |
| 181 | 1420384_at | Col4a3bp | collagen, type IV, alpha 3 (Goodpasture antigen) binding protein | + | 1.2 | 0.0081 |
| 182 | 1417752_at | Coro1c | coronin, actin binding protein 1C | - | 1.2 | 0.0017 |
| 183 | 1417747_at | Cplx1 | complexin 1 | - | 1.3 | 0.0015 |
| 184 | 1436383_at | Cplx2 | complexin 2 | - | 1.2 | 0.0057 |
| 185 | 1460176_at | Crk | v-crk sarcoma virus CT10 oncogene homolog (avian) | + | 1.4 | 0.0005 |
| 186 | 1422577_at | Cs | citrate synthase | - | 1.2 | 0.0059 |
| 187 | 1425810_a_at | Csrp1 | cysteine and glycine-rich protein 1 | - | 1.4 | 0.0013 |
| 188 | 1416274_at | Ctns | cystinosis, nephropathic | - | 1.6 | 0.0000 |
| 189 | 1422680_at | Ctr9 | Ctr9, Paf1/RNA polymerase II complex component, homolog (S. cerevisiae) | - | 1.2 | 0.0014 |
| 190 | 1448732_at | Ctsb | cathepsin B | - | 1.4 | 0.0023 |
| 191 | 1419728_at | Cxcl5 | chemokine (C-X-C motif) ligand 5 | - | 1.3 | 0.0008 |
| 192 | 1422186_s_at | Cyb5r3 | cytochrome b5 reductase 3 | - | 1.2 | 0.0065 |
| 193 | 1425329_a_at | Cyb5r3 | cytochrome b5 reductase 3 | - | 1.2 | 0.0064 |
| 194 | 1422185_a_at | Cyb5r3 | cytochrome b5 reductase 3 | - | 1.3 | 0.0007 |
| 195 | 1457271_at | Cym | chymosin | + | 1.4 | 0.0021 |
| 196 | 1448997_at | Cyth1 | cytohesin 1 | - | 1.3 | 0.0024 |
| 197 | 1446958_at | D13Ertd150e | DNA segment, Chr 13, ERATO Doi 150, expressed | + | 1.3 | 0.0044 |
| 198 | 1446564_at | D18Ertd169e | DNA segment, Chr 18, ERATO Doi 169, expressed | + | 1.3 | 0.0081 |
| 199 | 1452343_at | D18Ertd653e | DNA segment, Chr 18, ERATO Doi 653, expressed | - | 1.3 | 0.0057 |
| 200 | 1443458_at | D630033O11Rik | RIKEN cDNA D630033O11 gene | + | 1.3 | 0.0026 |
| 201 | 1442409_at | D9Wsu90e | DNA segment, Chr 9, Wayne State University 90, expressed | + | 1.2 | 0.0097 |
| 202 | 1423872_a_at | Dag1 | dystroglycan 1 | - | 1.2 | 0.0073 |
| 203 | 1423446_at | Dapk3 | death-associated protein kinase 3 | - | 1.2 | 0.0073 |
| 204 | 1455079_at | Dcun1d4 | DCN1, defective in cullin neddylation 1, domain containing 4 (S. cerevisiae) | + | 1.2 | 0.0079 |
| 205 | 1423495_at | Decr2 | 2-4-dienoyl-Coenzyme A reductase 2, peroxisomal | - | 1.3 | 0.0004 |
| 206 | 1422677_at | Dgat2 | diacylglycerol O-acyltransferase 2 | - | 1.3 | 0.0002 |
| 207 | 1416821_at | Dgcr14 | DiGeorge syndrome critical region gene 14 | - | 1.3 | 0.0029 |
| 208 | 1438765_at | Dhx33 | DEAH (Asp-Glu-Ala-His) box polypeptide 33 | - | 1.3 | 0.0008 |
| 209 | 1431258_at | Dido1 | death inducer-obliterator 1 | + | 1.4 | 0.0034 |
| 210 | 1418937_at | Dio2 | deiodinase, iodothyronine, type II | + | 1.2 | 0.0053 |
| 211 | 1426081_a_at | Dio2 | deiodinase, iodothyronine, type II | + | 1.2 | 0.0049 |
| 212 | 1420335_at | Dmc1 | DMC1 dosage suppressor of mck1 homolog, meiosis-specific homologous recombination (yeast) | + | 1.3 | 0.0003 |
| 213 | 1420182_x_at | Dmrt3 | doublesex and mab-3 related transcription factor 3 | - | 1.2 | 0.0091 |
| 214 | 1427252_at | Dmrtb1 | DMRT-like family B with proline-rich C-terminal, 1 | - | 1.2 | 0.0096 |
| 215 | 1436296_x_at | Dnaic2 | dynein, axonemal, intermediate chain 2 | - | 1.3 | 0.0077 |
| 216 | 1420629_a_at | Dnaja3 | DnaJ (Hsp40) homolog, subfamily A, member 3 | - | 1.2 | 0.0007 |
| 217 | 1418592_at | Dnaja4 | DnaJ (Hsp40) homolog, subfamily A, member 4 | - | 1.2 | 0.0055 |
| 218 | 1420165_s_at | Dnajc17 | DnaJ (Hsp40) homolog, subfamily C, member 17 | - | 1.3 | 0.0053 |
| 219 | 1431215_at | Dnajc6 | DnaJ (Hsp40) homolog, subfamily C, member 6 | - | 1.3 | 0.0099 |
| 220 | 1427754_a_at | Dnm1 | dynamin 1 | - | 1.3 | 0.0058 |
| 221 | 1451676_at | Drap1 | Dr1 associated protein 1 (negative cofactor 2 alpha) | - | 1.2 | 0.0080 |
| 222 | 1422830_s_at | Drd4 | dopamine receptor 4 | - | 1.4 | 0.0011 |
| 223 | 1417207_at | Dvl2 | dishevelled 2, dsh homolog (Drosophila) | - | 1.2 | 0.0088 |
| 224 | 1441924_x_at | Edn3 | endothelin 3 | + | 2.1 | 0.0012 |
| 225 | 1416964_at | Eefsec | eukaryotic elongation factor, selenocysteine-tRNA-specific | - | 1.2 | 0.0091 |
| 226 | 1448507_at | Efhd1 | EF hand domain containing 1 | - | 1.2 | 0.0053 |
| 227 | 1444606_at | Efna2 | ephrin A2 | - | 1.2 | 0.0081 |
| 228 | 1432647_at | Egfr | epidermal growth factor receptor | + | 1.3 | 0.0065 |
| 229 | 1435054_at | Eme1 | essential meiotic endonuclease 1 homolog 1 (S. pombe) | + | 1.2 | 0.0090 |
| 230 | 1426541_a_at | Endod1 | endonuclease domain containing 1 | - | 1.3 | 0.0095 |
| 231 | 1425353_at | Enpp3 | ectonucleotide pyrophosphatase/phosphodiesterase 3 | + | 1.2 | 0.0026 |
| 232 | 1425199_a_at | Epb4.1l5 | erythrocyte protein band 4.1-like 5 | - | 1.3 | 0.0004 |
| 233 | 1421815_at | Epdr1 | ependymin related protein 1 (zebrafish) | - | 1.3 | 0.0070 |
| 234 | 1422438_at | Ephx1 | epoxide hydrolase 1, microsomal | - | 1.3 | 0.0057 |
| 235 | 1432235_at | Epsti1 | epithelial stromal interaction 1 (breast) | + | 1.3 | 0.0076 |
| 236 | 1420602_a_at | Esx1 | extraembryonic, spermatogenesis, homeobox 1 | - | 1.2 | 0.0034 |
| 237 | 1433515_s_at | Etnk1 | ethanolamine kinase 1 | - | 1.4 | 0.0036 |
| 238 | 1423587_a_at | Exosc10 | exosome component 10 | - | 1.2 | 0.0016 |
| 239 | 1426960_a_at | Fa2h | fatty acid 2-hydroxylase | - | 1.3 | 0.0021 |
| 240 | 1450682_at | Fabp6 | fatty acid binding protein 6, ileal (gastrotropin) | - | 1.3 | 0.0016 |
| 241 | 1441123_at | Fam120c | family with sequence similarity 120, member C | + | 1.2 | 0.0058 |
| 242 | 1421595_at | Fam184b | family with sequence similarity 184, member B | + | 1.2 | 0.0026 |
| 243 | 1421070_at | Fam48a | family with sequence similarity 48, member A | - | 1.3 | 0.0087 |
| 244 | 1456163_at | Fam72a | family with sequence similarity 72, member A | + | 1.2 | 0.0092 |
| 245 | 1433657_at | Fam78a | family with sequence similarity 78, member A | + | 1.2 | 0.0067 |
| 246 | 1418856_a_at | Fanca | Fanconi anemia, complementation group A | + | 1.2 | 0.0085 |
| 247 | 1446226_at | Fancb | Fanconi anemia, complementation group B | - | 1.3 | 0.0063 |
| 248 | 1424197_s_at | Fance | Fanconi anemia, complementation group E | - | 1.2 | 0.0059 |
| 249 | 1460635_at | Fastk | Fas-activated serine/threonine kinase | - | 1.2 | 0.0072 |
| 250 | 1460208_at | Fbn1 | fibrillin 1 | - | 1.2 | 0.0034 |
| 251 | 1448420_a_at | Fbxl12 | F-box and leucine-rich repeat protein 12 | - | 1.2 | 0.0099 |
| 252 | 1451543_at | Fbxo21 | F-box protein 21 | - | 1.3 | 0.0018 |
| 253 | 1421543_at | Fbxo4 | F-box protein 4 | + | 1.2 | 0.0062 |
| 254 | 1450869_at | Fgf1 | fibroblast growth factor 1 | - | 1.2 | 0.0039 |
| 255 | 1418498_at | Fgf13 | fibroblast growth factor 13 | - | 1.4 | 0.0091 |
| 256 | 1460296_a_at | Fgf22 | fibroblast growth factor 22 | + | 1.3 | 0.0049 |
| 257 | 1425911_a_at | Fgfr1 | fibroblast growth factor receptor 1 | - | 1.3 | 0.0028 |
| 258 | 1427776_a_at | Fgfr4 | fibroblast growth factor receptor 4 | - | 1.3 | 0.0032 |
| 259 | 1416113_at | Fkbp8 | FK506 binding protein 8 | - | 1.4 | 0.0014 |
| 260 | 1437536_at | Fkrp | fukutin related protein | - | 1.2 | 0.0087 |
| 261 | 1426677_at | Flna | filamin, alpha | - | 1.1 | 0.0093 |
| 262 | 1417544_a_at | Flot2 | flotillin 2 | - | 1.2 | 0.0089 |
| 263 | 1451752_at | Foxk1 | forkhead box K1 | + | 1.3 | 0.0056 |
| 264 | 1420765_a_at | Foxp3 | forkhead box P3 | + | 1.4 | 0.0019 |
| 265 | 1427617_at | Fut10 | fucosyltransferase 10 | + | 1.4 | 0.0002 |
| 266 | 1419451_at | Fzr1 | fizzy/cell division cycle 20 related 1 (Drosophila) | - | 1.2 | 0.0047 |
| 267 | 1446657_at | Galnt6 | UDP-N-acetyl-alpha-D-galactosamine:polypeptide N-acetylgalactosaminyltransferase 6 | - | 1.2 | 0.0040 |
| 268 | 1445685_at | Gas7 | growth arrest specific 7 | + | 1.3 | 0.0018 |
| 269 | 1451197_s_at | Gatad2a | GATA zinc finger domain containing 2A | - | 1.2 | 0.0031 |
| 270 | 1437756_at | Gimap9 | GTPase, IMAP family member 9 | + | 1.3 | 0.0018 |
| 271 | 1454082_a_at | Giyd2 | GIY-YIG domain containing 2 | - | 1.2 | 0.0096 |
| 272 | 1448767_s_at | Gjb1 | gap junction protein, beta 1 | - | 1.4 | 0.0032 |
| 273 | 1422179_at | Gjb4 | gap junction protein, beta 4 | - | 1.2 | 0.0070 |
| 274 | 1436210_at | Gk5 | glycerol kinase 5 (putative) | + | 1.2 | 0.0042 |
| 275 | 1428421_a_at | Glod4 | glyoxalase domain containing 4 | - | 1.2 | 0.0035 |
| 276 | 1440361_at | Gm12371 | predicted gene 12371 | + | 1.4 | 0.0039 |
| 277 | 1457933_at | Gm1964 | predicted gene 1964 | + | 1.2 | 0.0022 |
| 278 | 1416188_at | Gm2a | GM2 ganglioside activator protein | - | 1.4 | 0.0022 |
| 279 | 1446741_at | Gm5463 | predicted gene 5463 | + | 1.2 | 0.0065 |
| 280 | 1437004_at | Gm6021 | predicted gene 6021 | + | 1.2 | 0.0093 |
| 281 | 1443268_at | Gm765 | predicted gene 765 | + | 1.2 | 0.0068 |
| 282 | 1443995_at | Gm9 | predicted gene 9 | + | 1.2 | 0.0039 |
| 283 | 1450097_s_at | Gna12 | guanine nucleotide binding protein, alpha 12 | - | 1.3 | 0.0015 |
| 284 | 1419449_a_at | Gnai2 | guanine nucleotide binding protein (G protein), alpha inhibiting 2 | - | 1.3 | 0.0044 |
| 285 | 1460460_a_at | Gorasp2 | golgi reassembly stacking protein 2 | - | 1.4 | 0.0026 |
| 286 | 1416215_at | Gosr1 | golgi SNAP receptor complex member 1 | - | 1.2 | 0.0043 |
| 287 | 1430238_at | Got1l1 | glutamic-oxaloacetic transaminase 1-like 1 | - | 1.2 | 0.0039 |
| 288 | 1417716_at | Got2 | glutamate oxaloacetate transaminase 2, mitochondrial | - | 1.2 | 0.0071 |
| 289 | 1460123_at | Gpr1 | G protein-coupled receptor 1 | + | 1.2 | 0.0031 |
| 290 | 1455828_at | Gprin1 | G protein-regulated inducer of neurite outgrowth 1 | + | 1.4 | 0.0088 |
| 291 | 1424137_at | Gprin1 | G protein-regulated inducer of neurite outgrowth 1 | - | 1.3 | 0.0029 |
| 292 | 1430834_at | Gprin3 | GPRIN family member 3 | + | 1.2 | 0.0091 |
| 293 | 1417796_at | Gps2 | G protein pathway suppressor 2 | - | 1.2 | 0.0022 |
| 294 | 1418492_at | Grem2 | gremlin 2 homolog, cysteine knot superfamily (Xenopus laevis) | + | 1.2 | 0.0073 |
| 295 | 1418784_at | Grik5 | glutamate receptor, ionotropic, kainate 5 (gamma 2) | - | 1.3 | 0.0061 |
| 296 | 1450202_at | Grin1 | glutamate receptor, ionotropic, NMDA1 (zeta 1) | - | 1.3 | 0.0032 |
| 297 | 1453709_at | Grit | Rho GTPase-activating protein | + | 1.2 | 0.0036 |
| 298 | 1419072_at | Gstm7 | glutathione S-transferase, mu 7 | - | 1.2 | 0.0094 |
| 299 | 1443213_at | Gtdc1 | glycosyltransferase-like domain containing 1 | + | 1.3 | 0.0068 |
| 300 | 1460279_a_at | Gtf2i | general transcription factor II I | - | 1.3 | 0.0022 |
| 301 | 1420625_at | Gtf3c6 | general transcription factor IIIC, polypeptide 6, alpha | - | 1.3 | 0.0010 |
| 302 | 1453771_at | Gulp1 | GULP, engulfment adaptor PTB domain containing 1 | - | 1.3 | 0.0029 |
| 303 | 1452544_x_at | H2-D1 | histocompatibility 2, D region locus 1 | - | 1.4 | 0.0000 |
| 304 | 1430869_a_at | Habp4 | hyaluronic acid binding protein 4 | - | 1.3 | 0.0037 |
| 305 | 1447566_at | Hdac4 | histone deacetylase 4 | - | 1.2 | 0.0024 |
| 306 | 1454713_s_at | Hdc | histidine decarboxylase | - | 1.3 | 0.0012 |
| 307 | 1425983_x_at | Hipk2 | homeodomain interacting protein kinase 2 | - | 1.2 | 0.0078 |
| 308 | 1416101_a_at | Hist1h1c | histone cluster 1, H1c | - | 1.2 | 0.0042 |
| 309 | 1450608_at | Hist1h1t | histone cluster 1, H1t | - | 1.2 | 0.0060 |
| 310 | 1420901_a_at | Hk1 | hexokinase 1 | - | 1.3 | 0.0071 |
| 311 | 1416184_s_at | Hmga1 | high mobility group AT-hook 1 | - | 1.2 | 0.0045 |
| 312 | 1421234_at | Hnf1a | HNF1 homeobox A | + | 1.4 | 0.0013 |
| 313 | 1442816_at | Hs3st3b1 | heparan sulfate (glucosamine) 3-O-sulfotransferase 3B1 | + | 1.2 | 0.0039 |
| 314 | 1425786_a_at | Hsf4 | heat shock transcription factor 4 | - | 1.2 | 0.0054 |
| 315 | 1440575_at | Hspa4 | heat shock protein 4 | + | 1.3 | 0.0015 |
| 316 | 1427216_at | Ifnz | interferon zeta | + | 1.2 | 0.0086 |
| 317 | 1421991_a_at | Igfbp4 | insulin-like growth factor binding protein 4 | - | 1.2 | 0.0043 |
| 318 | 1423756_s_at | Igfbp4 | insulin-like growth factor binding protein 4 | - | 1.2 | 0.0082 |
| 319 | 1423757_x_at | Igfbp4 | insulin-like growth factor binding protein 4 | - | 1.2 | 0.0066 |
| 320 | 1421992_a_at | Igfbp4 | insulin-like growth factor binding protein 4 | - | 1.2 | 0.0060 |
| 321 | 1425625_at | Il13ra1 | interleukin 13 receptor, alpha 1 | + | 1.2 | 0.0023 |
| 322 | 1420904_at | Il17ra | interleukin 17 receptor A | + | 1.2 | 0.0018 |
| 323 | 1419671_a_at | Il17rc | interleukin 17 receptor C | - | 1.3 | 0.0040 |
| 324 | 1421473_at | Il1a | interleukin 1 alpha | + | 1.2 | 0.0046 |
| 325 | 1423017_a_at | Il1rn | interleukin 1 receptor antagonist | + | 1.3 | 0.0086 |
| 326 | 1429533_at | Immt | inner membrane protein, mitochondrial | - | 1.3 | 0.0055 |
| 327 | 1418657_at | Ino80b | INO80 complex subunit B | - | 1.4 | 0.0054 |
| 328 | 1438446_x_at | Inpp5k | inositol polyphosphate 5-phosphatase K | - | 1.1 | 0.0079 |
| 329 | 1448668_a_at | Irak1 | interleukin-1 receptor-associated kinase 1 | - | 1.2 | 0.0053 |
| 330 | 1423238_at | Itgb1bp2 | integrin beta 1 binding protein 2 | + | 1.2 | 0.0064 |
| 331 | 1420665_at | Itgb3bp | integrin beta 3 binding protein (beta3-endonexin) | + | 1.2 | 0.0071 |
| 332 | 1427387_a_at | Itgb4 | integrin beta 4 | - | 1.2 | 0.0024 |
| 333 | 1430597_at | Jakmip1 | janus kinase and microtubule interacting protein 1 | - | 1.3 | 0.0086 |
| 334 | 1419288_at | Jam2 | junction adhesion molecule 2 | - | 1.3 | 0.0095 |
| 335 | 1449046_a_at | Josd2 | Josephin domain containing 2 | - | 1.2 | 0.0079 |
| 336 | 1426873_s_at | Jup | junction plakoglobin | - | 1.3 | 0.0081 |
| 337 | 1425885_a_at | Kcnab2 | potassium voltage-gated channel, shaker-related subfamily, beta member 2 | - | 1.3 | 0.0006 |
| 338 | 1425870_a_at | Kcnip2 | Kv channel-interacting protein 2 | - | 1.6 | 0.0006 |
| 339 | 1450185_a_at | Kcnj15 | potassium inwardly-rectifying channel, subfamily J, member 15 | + | 1.3 | 0.0085 |
| 340 | 1455896_a_at | Kcnk1 | potassium channel, subfamily K, member 1 | - | 1.2 | 0.0044 |
| 341 | 1450747_at | Keap1 | kelch-like ECH-associated protein 1 | - | 1.2 | 0.0015 |
| 342 | 1449057_at | Kel | Kell blood group | + | 1.2 | 0.0068 |
| 343 | 1450108_at | Kif1a | kinesin family member 1A | - | 1.3 | 0.0019 |
| 344 | 1450692_at | Kif4 | kinesin family member 4 | + | 1.2 | 0.0072 |
| 345 | 1448541_at | Klc1 | kinesin light chain 1 | - | 1.5 | 0.0048 |
| 346 | 1451739_at | Klf5 | Kruppel-like factor 5 | + | 1.3 | 0.0096 |
| 347 | 1436952_at | Klf9 | Kruppel-like factor 9 | - | 1.4 | 0.0057 |
| 348 | 1448164_at | Klhdc3 | kelch domain containing 3 | - | 1.2 | 0.0078 |
| 349 | 1420770_at | Klk1b24 | kallikrein 1-related peptidase b24 | + | 1.4 | 0.0035 |
| 350 | 1425888_at | Klra17 | killer cell lectin-like receptor, subfamily A, member 17 | + | 1.3 | 0.0019 |
| 351 | 1426136_x_at | Klra21 | killer cell lectin-like receptor subfamily A, member 21 | + | 1.4 | 0.0009 |
| 352 | 1418213_at | Krt23 | keratin 23 | - | 1.2 | 0.0029 |
| 353 | 1450540_x_at | Krtap5-1 | keratin associated protein 5-1 | + | 1.3 | 0.0014 |
| 354 | 1450114_at | Ksr1 | kinase suppressor of ras 1 | - | 1.3 | 0.0085 |
| 355 | 1427009_at | Lama5 | laminin, alpha 5 | - | 1.2 | 0.0059 |
| 356 | 1416343_a_at | Lamp2 | lysosomal-associated membrane protein 2 | - | 1.4 | 0.0098 |
| 357 | 1423955_a_at | Lass2 | LAG1 homolog, ceramide synthase 2 | - | 1.2 | 0.0055 |
| 358 | 1419317_x_at | Lce3c | late cornified envelope 3C | + | 1.2 | 0.0075 |
| 359 | 1435152_at | Leng8 | leukocyte receptor cluster (LRC) member 8 | - | 1.3 | 0.0053 |
| 360 | 1421217_a_at | Lgals9 | lectin, galactose binding, soluble 9 | - | 1.4 | 0.0001 |
| 361 | 1440968_at | Lgtn | ligatin | - | 1.2 | 0.0036 |
| 362 | 1418231_at | Lims1 | LIM and senescent cell antigen-like domains 1 | - | 1.3 | 0.0028 |
| 363 | 1449262_s_at | Lin7c | lin-7 homolog C (C. elegans) | - | 1.2 | 0.0087 |
| 364 | 1443984_at | Lin9 | lin-9 homolog (C. elegans) | + | 1.2 | 0.0076 |
| 365 | 1427998_at | Lsm12 | LSM12 homolog (S. cerevisiae) | - | 1.2 | 0.0076 |
| 366 | 1453304_s_at | Ly6e | lymphocyte antigen 6 complex, locus E | - | 1.3 | 0.0010 |
| 367 | 1431607_at | Lypla1 | lysophospholipase 1 | + | 1.3 | 0.0025 |
| 368 | 1443058_at | Macrod2 | MACRO domain containing 2 | - | 1.2 | 0.0061 |
| 369 | 1460219_at | Mag | myelin-associated glycoprotein | - | 1.3 | 0.0043 |
| 370 | 1449901_a_at | Map3k6 | mitogen-activated protein kinase kinase kinase 6 | + | 1.2 | 0.0073 |
| 371 | 1425679_a_at | Mapk8ip1 | mitogen-activated protein kinase 8 interacting protein 1 | - | 1.2 | 0.0081 |
| 372 | 1416437_a_at | Mapk8ip3 | mitogen-activated protein kinase 8 interacting protein 3 | - | 1.3 | 0.0032 |
| 373 | 1427079_at | Mapre3 | microtubule-associated protein, RP/EB family, member 3 | - | 1.3 | 0.0024 |
| 374 | 1417885_at | Mapt | microtubule-associated protein tau | - | 1.3 | 0.0013 |
| 375 | 1455346_at | Masp1 | mannan-binding lectin serine peptidase 1 | - | 1.4 | 0.0003 |
| 376 | 1454591_at | Mast4 | microtubule associated serine/threonine kinase family member 4 | + | 1.3 | 0.0029 |
| 377 | 1451176_at | Mavs | mitochondrial antiviral signaling protein | - | 1.3 | 0.0006 |
| 378 | 1449490_at | Mbd4 | methyl-CpG binding domain protein 4 | + | 1.3 | 0.0006 |
| 379 | 1451961_a_at | Mbp | myelin basic protein | - | 1.5 | 0.0013 |
| 380 | 1416357_a_at | Mcam | melanoma cell adhesion molecule | - | 1.2 | 0.0023 |
| 381 | 1448374_at | Med28 | mediator of RNA polymerase II transcription, subunit 28 homolog (yeast) | - | 1.2 | 0.0026 |
| 382 | 1426837_at | Metap1 | methionyl aminopeptidase 1 | - | 1.3 | 0.0034 |
| 383 | 1416358_at | Mfsd10 | major facilitator superfamily domain containing 10 | - | 1.2 | 0.0085 |
| 384 | 1431285_at | Mgrn1 | mahogunin, ring finger 1 | - | 1.3 | 0.0018 |
| 385 | 1452592_at | Mgst2 | microsomal glutathione S-transferase 2 | + | 1.5 | 0.0004 |
| 386 | 1451685_at | Mllt6 | myeloid/lymphoid or mixed-lineage leukemia (trithorax homolog, Drosophila); translocated to, 6 | - | 1.2 | 0.0047 |
| 387 | 1421010_at | Mobp | myelin-associated oligodendrocytic basic protein | - | 1.2 | 0.0088 |
| 388 | 1450088_a_at | Mobp | myelin-associated oligodendrocytic basic protein | - | 1.3 | 0.0054 |
| 389 | 1448768_at | Mog | myelin oligodendrocyte glycoprotein | - | 1.3 | 0.0084 |
| 390 | 1440915_at | Mphosph9 | M-phase phosphoprotein 9 | - | 1.2 | 0.0055 |
| 391 | 1430771_a_at | Msh5 | mutS homolog 5 (E. coli) | + | 1.3 | 0.0090 |
| 392 | 1429894_a_at | Mtap7 | microtubule-associated protein 7 | - | 1.2 | 0.0067 |
| 393 | 1421835_at | Mtap7 | microtubule-associated protein 7 | - | 1.3 | 0.0023 |
| 394 | 1421879_at | Mtmr1 | myotubularin related protein 1 | - | 1.2 | 0.0079 |
| 395 | 1419399_at | Mttp | microsomal triglyceride transfer protein | + | 1.2 | 0.0007 |
| 396 | 1436501_at | Mtus1 | mitochondrial tumor suppressor 1 | - | 1.3 | 0.0088 |
| 397 | 1427938_at | Mycbp | c-myc binding protein | - | 1.2 | 0.0047 |
| 398 | 1442858_at | Mycbp2 | MYC binding protein 2 | + | 1.2 | 0.0080 |
| 399 | 1427520_a_at | Myh1 | myosin, heavy polypeptide 1, skeletal muscle, adult | + | 1.2 | 0.0083 |
| 400 | 1428509_at | Myo1e | myosin IE | - | 1.4 | 0.0049 |
| 401 | 1418420_at | Myod1 | myogenic differentiation 1 | + | 1.2 | 0.0048 |
| 402 | 1446189_at | Myst4 | MYST histone acetyltransferase monocytic leukemia 4 | + | 1.5 | 0.0023 |
| 403 | 1435269_at | N6amt2 | N-6 adenine-specific DNA methyltransferase 2 (putative) | - | 1.2 | 0.0043 |
| 404 | 1454378_at | NA | NA | + | 1.8 | 0.0078 |
| 405 | 1441066_at | NA | NA | + | 1.7 | 0.0028 |
| 406 | 1445617_at | NA | NA | + | 1.6 | 0.0000 |
| 407 | 1449727_x_at | NA | NA | + | 1.5 | 0.0029 |
| 408 | 1446567_at | NA | NA | + | 1.5 | 0.0032 |
| 409 | 1430477_s_at | NA | NA | + | 1.5 | 0.0005 |
| 410 | 1446880_at | NA | NA | + | 1.4 | 0.0017 |
| 411 | 1419915_at | NA | NA | + | 1.4 | 0.0050 |
| 412 | 1417711_at | NA | NA | + | 1.4 | 0.0093 |
| 413 | 1442857_at | NA | NA | + | 1.4 | 0.0052 |
| 414 | 1447226_at | NA | NA | + | 1.4 | 0.0097 |
| 415 | 1449790_at | NA | NA | + | 1.4 | 0.0029 |
| 416 | 1442880_at | NA | NA | + | 1.4 | 0.0069 |
| 417 | 1459297_at | NA | NA | + | 1.4 | 0.0066 |
| 418 | 1443090_at | NA | NA | + | 1.4 | 0.0001 |
| 419 | 1432840_at | NA | NA | + | 1.3 | 0.0019 |
| 420 | 1444622_at | NA | NA | + | 1.3 | 0.0044 |
| 421 | 1440606_at | NA | NA | + | 1.3 | 0.0096 |
| 422 | 1458173_at | NA | NA | + | 1.3 | 0.0059 |
| 423 | 1445382_at | NA | NA | + | 1.3 | 0.0074 |
| 424 | 1444286_at | NA | NA | + | 1.3 | 0.0057 |
| 425 | 1445508_at | NA | NA | + | 1.3 | 0.0066 |
| 426 | 1433064_at | NA | NA | + | 1.3 | 0.0032 |
| 427 | 1459435_at | NA | NA | + | 1.3 | 0.0044 |
| 428 | 1444841_at | NA | NA | + | 1.3 | 0.0079 |
| 429 | 1460002_at | NA | NA | + | 1.3 | 0.0021 |
| 430 | 1439053_at | NA | NA | + | 1.3 | 0.0030 |
| 431 | 1458127_at | NA | NA | + | 1.3 | 0.0080 |
| 432 | 1434110_x_at | NA | NA | + | 1.3 | 0.0018 |
| 433 | 1458645_at | NA | NA | + | 1.3 | 0.0025 |
| 434 | 1427854_x_at | NA | NA | + | 1.3 | 0.0071 |
| 435 | 1420318_at | NA | NA | + | 1.3 | 0.0087 |
| 436 | 1445834_at | NA | NA | + | 1.3 | 0.0005 |
| 437 | 1458872_at | NA | NA | + | 1.3 | 0.0030 |
| 438 | 1457503_at | NA | NA | + | 1.3 | 0.0094 |
| 439 | 1444912_at | NA | NA | + | 1.3 | 0.0099 |
| 440 | 1442163_at | NA | NA | + | 1.3 | 0.0035 |
| 441 | 1445238_at | NA | NA | + | 1.3 | 0.0060 |
| 442 | 1447092_at | NA | NA | + | 1.3 | 0.0094 |
| 443 | 1457312_at | NA | NA | + | 1.3 | 0.0049 |
| 444 | 1456650_at | NA | NA | + | 1.3 | 0.0073 |
| 445 | 1458138_at | NA | NA | + | 1.3 | 0.0037 |
| 446 | 1443799_at | NA | NA | + | 1.3 | 0.0004 |
| 447 | 1457016_at | NA | NA | + | 1.2 | 0.0087 |
| 448 | 1436259_at | NA | NA | + | 1.2 | 0.0095 |
| 449 | 1445556_at | NA | NA | + | 1.2 | 0.0058 |
| 450 | 1443390_at | NA | NA | + | 1.2 | 0.0024 |
| 451 | 1441332_at | NA | NA | + | 1.2 | 0.0089 |
| 452 | 1441654_at | NA | NA | + | 1.2 | 0.0011 |
| 453 | 1440515_at | NA | NA | + | 1.2 | 0.0059 |
| 454 | 1430590_at | NA | NA | + | 1.2 | 0.0025 |
| 455 | 1460153_at | NA | NA | + | 1.2 | 0.0013 |
| 456 | 1443243_at | NA | NA | + | 1.2 | 0.0033 |
| 457 | 1441472_at | NA | NA | + | 1.2 | 0.0021 |
| 458 | 1440948_at | NA | NA | + | 1.2 | 0.0042 |
| 459 | 1450607_s_at | NA | NA | + | 1.2 | 0.0080 |
| 460 | 1444344_at | NA | NA | + | 1.2 | 0.0071 |
| 461 | 1446894_at | NA | NA | + | 1.2 | 0.0020 |
| 462 | 1439299_at | NA | NA | + | 1.2 | 0.0068 |
| 463 | 1457880_at | NA | NA | + | 1.2 | 0.0085 |
| 464 | 1447430_at | NA | NA | + | 1.2 | 0.0096 |
| 465 | 1444208_at | NA | NA | + | 1.2 | 0.0071 |
| 466 | 1459908_at | NA | NA | + | 1.2 | 0.0082 |
| 467 | 1442184_at | NA | NA | + | 1.2 | 0.0085 |
| 468 | 1458653_at | NA | NA | + | 1.2 | 0.0056 |
| 469 | 1458604_at | NA | NA | + | 1.2 | 0.0090 |
| 470 | 1446875_at | NA | NA | + | 1.2 | 0.0060 |
| 471 | 1442543_at | NA | NA | + | 1.2 | 0.0091 |
| 472 | 1447491_at | NA | NA | + | 1.2 | 0.0034 |
| 473 | 1444750_at | NA | NA | + | 1.2 | 0.0074 |
| 474 | 1444048_at | NA | NA | + | 1.2 | 0.0092 |
| 475 | 1458075_at | NA | NA | + | 1.2 | 0.0086 |
| 476 | 1440266_at | NA | NA | + | 1.2 | 0.0086 |
| 477 | 1441164_at | NA | NA | + | 1.2 | 0.0081 |
| 478 | 1440080_at | NA | NA | + | 1.2 | 0.0091 |
| 479 | 1430788_at | NA | NA | - | 1.2 | 0.0097 |
| 480 | 1426657_s_at | NA | NA | - | 1.2 | 0.0062 |
| 481 | 1451132_at | NA | NA | - | 1.2 | 0.0071 |
| 482 | 1421816_at | NA | NA | - | 1.2 | 0.0085 |
| 483 | 1456861_at | NA | NA | - | 1.2 | 0.0096 |
| 484 | 1450822_at | NA | NA | - | 1.2 | 0.0032 |
| 485 | 1446730_at | NA | NA | - | 1.2 | 0.0094 |
| 486 | 1450882_s_at | NA | NA | - | 1.2 | 0.0079 |
| 487 | 1429154_at | NA | NA | - | 1.2 | 0.0045 |
| 488 | 1415789_a_at | NA | NA | - | 1.2 | 0.0088 |
| 489 | 1440876_at | NA | NA | - | 1.2 | 0.0059 |
| 490 | 1457343_at | NA | NA | - | 1.2 | 0.0045 |
| 491 | 1435150_at | NA | NA | - | 1.2 | 0.0033 |
| 492 | 1445763_at | NA | NA | - | 1.2 | 0.0081 |
| 493 | 1451392_at | NA | NA | - | 1.2 | 0.0020 |
| 494 | 1459660_at | NA | NA | - | 1.2 | 0.0016 |
| 495 | 1450393_a_at | NA | NA | - | 1.2 | 0.0048 |
| 496 | 1440306_at | NA | NA | - | 1.2 | 0.0034 |
| 497 | 1431314_a_at | NA | NA | - | 1.2 | 0.0027 |
| 498 | 1443623_a_at | NA | NA | - | 1.3 | 0.0053 |
| 499 | 1441797_at | NA | NA | - | 1.3 | 0.0078 |
| 500 | 1447479_at | NA | NA | - | 1.3 | 0.0067 |
| 501 | 1442386_at | NA | NA | - | 1.3 | 0.0079 |
| 502 | 1454336_at | NA | NA | - | 1.3 | 0.0040 |
| 503 | 1422068_at | NA | NA | - | 1.3 | 0.0034 |
| 504 | 1457330_at | NA | NA | - | 1.3 | 0.0035 |
| 505 | 1443354_at | NA | NA | - | 1.3 | 0.0008 |
| 506 | 1442283_at | NA | NA | - | 1.3 | 0.0041 |
| 507 | 1440512_at | NA | NA | - | 1.3 | 0.0099 |
| 508 | 1457082_at | NA | NA | - | 1.3 | 0.0014 |
| 509 | 1434183_at | NA | NA | - | 1.3 | 0.0029 |
| 510 | 1421787_at | NA | NA | - | 1.3 | 0.0004 |
| 511 | 1435629_at | NA | NA | - | 1.3 | 0.0001 |
| 512 | 1446703_at | NA | NA | - | 1.4 | 0.0053 |
| 513 | 1451522_s_at | NA | NA | - | 1.4 | 0.0040 |
| 514 | 1421895_at | NA | NA | - | 1.4 | 0.0025 |
| 515 | 1446515_at | NA | NA | - | 1.4 | 0.0021 |
| 516 | 1421842_a_at | NA | NA | - | 1.4 | 0.0037 |
| 517 | 1442910_at | NA | NA | - | 1.4 | 0.0023 |
| 518 | AFFX-r2-Bs-thr-5_s_at | NA | NA | - | 1.4 | 0.0085 |
| 519 | 1456947_at | NA | NA | - | 1.4 | 0.0002 |
| 520 | 1423916_s_at | NA | NA | - | 1.4 | 0.0013 |
| 521 | AFFX-ThrX-5_at | NA | NA | - | 1.4 | 0.0060 |
| 522 | 1437183_at | NA | NA | - | 1.5 | 0.0020 |
| 523 | 1425174_at | NA | NA | - | 1.5 | 0.0033 |
| 524 | 1441404_at | NA | NA | - | 1.8 | 0.0000 |
| 525 | 1444234_at | Napb | N-ethylmaleimide sensitive fusion protein attachment protein beta | + | 1.5 | 0.0018 |
| 526 | 1424203_at | Ncln | nicalin homolog (zebrafish) | - | 1.2 | 0.0069 |
| 527 | 1423413_at | Ndrg1 | N-myc downstream regulated gene 1 | - | 1.3 | 0.0041 |
| 528 | 1418881_at | Necab2 | N-terminal EF-hand calcium binding protein 2 | - | 1.2 | 0.0090 |
| 529 | 1418961_at | Necap2 | NECAP endocytosis associated 2 | - | 1.2 | 0.0097 |
| 530 | 1450382_at | Nf2 | neurofibromatosis 2 | - | 1.2 | 0.0044 |
| 531 | 1459909_at | Nfix | nuclear factor I/X | - | 1.2 | 0.0038 |
| 532 | 1423516_a_at | Nid2 | nidogen 2 | - | 1.4 | 0.0073 |
| 533 | 1420487_at | Nol7 | nucleolar protein 7 | + | 1.2 | 0.0089 |
| 534 | 1441075_at | Nostrin | nitric oxide synthase trafficker | + | 1.2 | 0.0085 |
| 535 | 1431329_at | Nphp4 | nephronophthisis 4 (juvenile) homolog (human) | + | 1.3 | 0.0025 |
| 536 | 1422790_at | Nppc | natriuretic peptide precursor type C | + | 1.2 | 0.0039 |
| 537 | 1427191_at | Npr2 | natriuretic peptide receptor 2 | - | 1.2 | 0.0022 |
| 538 | 1447211_at | Nrip1 | nuclear receptor interacting protein 1 | - | 1.2 | 0.0052 |
| 539 | 1455499_at | Nrxn2 | neurexin II | - | 1.3 | 0.0092 |
| 540 | 1419433_at | Nthl1 | nth (endonuclease III)-like 1 (E.coli) | + | 1.2 | 0.0021 |
| 541 | 1420837_at | Ntrk2 | neurotrophic tyrosine kinase, receptor, type 2 | - | 1.6 | 0.0003 |
| 542 | 1425070_at | Ntrk3 | neurotrophic tyrosine kinase, receptor, type 3 | - | 1.2 | 0.0078 |
| 543 | 1416903_at | Nucb1 | nucleobindin 1 | - | 1.3 | 0.0015 |
| 544 | 1446400_at | Olfr558 | olfactory receptor 558 | + | 1.3 | 0.0032 |
| 545 | 1422367_at | Olfr70 | olfactory receptor 70 | - | 1.2 | 0.0075 |
| 546 | 1424990_at | Orai1 | ORAI calcium release-activated calcium modulator 1 | - | 1.2 | 0.0071 |
| 547 | 1417705_at | Otub1 | OTU domain, ubiquitin aldehyde binding 1 | - | 1.2 | 0.0088 |
| 548 | 1438985_x_at | Otud5 | OTU domain containing 5 | + | 1.2 | 0.0036 |
| 549 | 1428953_at | Otud7b | OTU domain containing 7B | - | 1.2 | 0.0034 |
| 550 | 1422034_a_at | Palm | paralemmin | - | 1.3 | 0.0066 |
| 551 | 1421987_at | Papss2 | 3'-phosphoadenosine 5'-phosphosulfate synthase 2 | - | 1.2 | 0.0021 |
| 552 | 1455216_at | Paqr6 | progestin and adipoQ receptor family member VI | - | 1.2 | 0.0098 |
| 553 | 1449054_a_at | Pcbp4 | poly(rC) binding protein 4 | - | 1.3 | 0.0035 |
| 554 | 1422890_at | Pcdh18 | protocadherin 18 | - | 1.3 | 0.0057 |
| 555 | 1452913_at | Pcp4l1 | Purkinje cell protein 4-like 1 | - | 1.3 | 0.0031 |
| 556 | 1426184_a_at | Pdcd6ip | programmed cell death 6 interacting protein | - | 1.3 | 0.0064 |
| 557 | 1441967_at | Pddc1 | Parkinson disease 7 domain containing 1 | - | 1.3 | 0.0028 |
| 558 | 1417603_at | Per2 | period homolog 2 (Drosophila) | - | 1.3 | 0.0006 |
| 559 | 1435037_at | Pgap3 | post-GPI attachment to proteins 3 | - | 1.3 | 0.0038 |
| 560 | 1439527_at | Pgr | progesterone receptor | + | 1.2 | 0.0015 |
| 561 | 1418620_at | Phox2a | paired-like homeobox 2a | + | 1.3 | 0.0027 |
| 562 | 1437637_at | Phtf2 | putative homeodomain transcription factor 2 | - | 1.2 | 0.0093 |
| 563 | 1421013_at | Pitpnb | phosphatidylinositol transfer protein, beta | - | 1.4 | 0.0035 |
| 564 | 1420307_a_at | Pitpnb | phosphatidylinositol transfer protein, beta | - | 1.4 | 0.0000 |
| 565 | 1435053_s_at | Plekhh1 | pleckstrin homology domain containing, family H (with MyTH4 domain) member 1 | - | 1.4 | 0.0007 |
| 566 | 1452517_at | Plekhh1 | pleckstrin homology domain containing, family H (with MyTH4 domain) member 1 | - | 1.4 | 0.0016 |
| 567 | 1444817_at | Plekhh2 | pleckstrin homology domain containing, family H (with MyTH4 domain) member 2 | + | 1.6 | 0.0002 |
| 568 | 1448945_at | Pllp | plasma membrane proteolipid | - | 1.3 | 0.0008 |
| 569 | 1425467_a_at | Plp1 | proteolipid protein (myelin) 1 | - | 1.4 | 0.0008 |
| 570 | 1417963_at | Pltp | phospholipid transfer protein | - | 1.5 | 0.0008 |
| 571 | 1418750_at | Plxnb3 | plexin B3 | - | 1.2 | 0.0081 |
| 572 | 1419298_at | Pon3 | paraoxonase 3 | - | 1.4 | 0.0005 |
| 573 | 1448565_at | Ppp1r11 | protein phosphatase 1, regulatory (inhibitor) subunit 11 | - | 1.3 | 0.0054 |
| 574 | 1418086_at | Ppp1r14a | protein phosphatase 1, regulatory (inhibitor) subunit 14A | - | 1.3 | 0.0038 |
| 575 | 1451331_at | Ppp1r1b | protein phosphatase 1, regulatory (inhibitor) subunit 1B | - | 1.9 | 0.0001 |
| 576 | 1415819_a_at | Ppp2r1a | protein phosphatase 2 (formerly 2A), regulatory subunit A (PR 65), alpha isoform | - | 1.3 | 0.0086 |
| 577 | 1425725_s_at | Ppp2r5c | protein phosphatase 2, regulatory subunit B (B56), gamma isoform | - | 1.5 | 0.0006 |
| 578 | 1450368_a_at | Ppp3r1 | protein phosphatase 3, regulatory subunit B, alpha isoform (calcineurin B, type I) | - | 1.4 | 0.0069 |
| 579 | 1456805_a_at | Ppp4r1l | protein phosphatase 4, regulatory subunit 1-like | + | 1.2 | 0.0087 |
| 580 | 1431361_at | Prcp | prolylcarboxypeptidase (angiotensinase C) | + | 1.5 | 0.0020 |
| 581 | 1452457_a_at | Prkab1 | protein kinase, AMP-activated, beta 1 non-catalytic subunit | - | 1.3 | 0.0033 |
| 582 | 1421446_at | Prkcc | protein kinase C, gamma | - | 1.4 | 0.0007 |
| 583 | 1424287_at | Prkx | protein kinase, X-linked | - | 1.3 | 0.0039 |
| 584 | 1431768_a_at | Prmt3 | protein arginine N-methyltransferase 3 | - | 1.3 | 0.0085 |
| 585 | 1452834_at | Prr5l | proline rich 5 like | - | 1.2 | 0.0082 |
| 586 | 1438500_at | Prrt3 | proline-rich transmembrane protein 3 | - | 1.2 | 0.0051 |
| 587 | 1432129_a_at | Prrx1 | paired related homeobox 1 | + | 1.3 | 0.0013 |
| 588 | 1420352_at | Prss22 | protease, serine, 22 | + | 1.2 | 0.0086 |
| 589 | 1449805_at | Prss29 | protease, serine, 29 | + | 1.3 | 0.0031 |
| 590 | 1416240_at | Psmb7 | proteasome (prosome, macropain) subunit, beta type 7 | - | 1.3 | 0.0093 |
| 591 | 1453164_a_at | Ptdss2 | phosphatidylserine synthase 2 | - | 1.3 | 0.0039 |
| 592 | 1453578_at | Pter | phosphotriesterase related | + | 1.3 | 0.0053 |
| 593 | 1429474_at | Ptgr2 | prostaglandin reductase 2 | - | 1.2 | 0.0050 |
| 594 | 1452508_x_at | Ptms | parathymosin | - | 1.2 | 0.0049 |
| 595 | 1418182_at | Ptp4a3 | protein tyrosine phosphatase 4a3 | + | 1.3 | 0.0061 |
| 596 | 1433823_at | Ptpdc1 | protein tyrosine phosphatase domain containing 1 | - | 1.2 | 0.0022 |
| 597 | 1423448_at | Rab11b | RAB11B, member RAS oncogene family | - | 1.2 | 0.0055 |
| 598 | 1455857_a_at | Rab2b | RAB2B, member RAS oncogene family | - | 1.2 | 0.0079 |
| 599 | 1436923_at | Rab2b | RAB2B, member RAS oncogene family | - | 1.3 | 0.0016 |
| 600 | 1416527_at | Rab32 | RAB32, member RAS oncogene family | - | 1.3 | 0.0014 |
| 601 | 1455064_at | Rab36 | RAB36, member RAS oncogene family | - | 1.2 | 0.0035 |
| 602 | 1431136_at | Rab36 | RAB36, member RAS oncogene family | - | 1.3 | 0.0010 |
| 603 | 1453926_at | Rad54l | RAD54 like (S. cerevisiae) | + | 1.4 | 0.0045 |
| 604 | 1438031_at | Rasgrp3 | RAS, guanyl releasing protein 3 | - | 1.3 | 0.0030 |
| 605 | 1416354_at | Rbmx | RNA binding motif protein, X chromosome | - | 1.2 | 0.0034 |
| 606 | 1416601_a_at | Rcan1 | regulator of calcineurin 1 | - | 1.4 | 0.0082 |
| 607 | 1447634_x_at | Rcbtb2 | regulator of chromosome condensation (RCC1) and BTB (POZ) domain containing protein 2 | + | 1.2 | 0.0088 |
| 608 | 1448860_at | Rem2 | rad and gem related GTP binding protein 2 | - | 1.2 | 0.0072 |
| 609 | 1457387_at | Repin1 | replication initiator 1 | + | 1.3 | 0.0034 |
| 610 | 1424715_at | Retsat | retinol saturase (all trans retinol 13,14 reductase) | - | 1.2 | 0.0076 |
| 611 | 1451960_a_at | Rev3l | REV3-like, catalytic subunit of DNA polymerase zeta RAD54 like (S. cerevisiae) | + | 1.2 | 0.0095 |
| 612 | 1421160_a_at | Rfng | RFNG O-fucosylpeptide 3-beta-N-acetylglucosaminyltransferase | - | 1.3 | 0.0013 |
| 613 | 1433586_at | Rgmb | RGM domain family, member B | - | 1.2 | 0.0074 |
| 614 | 1451617_at | Rho | rhodopsin | + | 1.3 | 0.0040 |
| 615 | 1448605_at | Rhoc | ras homolog gene family, member C | - | 1.2 | 0.0084 |
| 616 | 1431684_at | Rlbp1l1 | retinaldehyde binding protein 1-like 1 | - | 1.2 | 0.0039 |
| 617 | 1431030_a_at | Rnf14 | ring finger protein 14 | - | 1.2 | 0.0017 |
| 618 | 1458303_at | Rnf165 | ring finger protein 165 | + | 1.2 | 0.0024 |
| 619 | 1430527_a_at | Rnf167 | ring finger protein 167 | - | 1.3 | 0.0008 |
| 620 | 1451157_at | Rnf187 | ring finger protein 187 | - | 1.4 | 0.0013 |
| 621 | 1432478_a_at | Rnf19b | ring finger protein 19B | - | 1.3 | 0.0007 |
| 622 | 1425793_a_at | Rorc | RAR-related orphan receptor gamma | - | 1.3 | 0.0040 |
| 623 | 1419799_at | Rpl27a | ribosomal protein L27A | + | 1.3 | 0.0083 |
| 624 | 1418896_a_at | Rpn2 | ribophorin II | - | 1.2 | 0.0019 |
| 625 | 1452383_at | Rps6ka3 | ribosomal protein S6 kinase polypeptide 3 | + | 1.2 | 0.0088 |
| 626 | 1422268_a_at | Rps6kb2 | ribosomal protein S6 kinase, polypeptide 2 | - | 1.4 | 0.0001 |
| 627 | 1451293_at | Rrp9 | RRP9, small subunit (SSU) processome component, homolog (yeast) | - | 1.2 | 0.0049 |
| 628 | 1425539_a_at | Rtn3 | reticulon 3 | - | 1.3 | 0.0029 |
| 629 | 1436868_at | Rtn4rl1 | reticulon 4 receptor-like 1 | - | 1.3 | 0.0009 |
| 630 | 1449246_at | Rundc3a | RUN domain containing 3A | - | 1.2 | 0.0086 |
| 631 | 1431137_at | Rusc1 | RUN and SH3 domain containing 1 | - | 1.2 | 0.0028 |
| 632 | 1421126_at | Ryr2 | ryanodine receptor 2, cardiac | + | 1.2 | 0.0032 |
| 633 | 1456977_at | Samd11 | sterile alpha motif domain containing 11 | + | 1.2 | 0.0097 |
| 634 | 1456902_at | Satb1 | special AT-rich sequence binding protein 1 | - | 1.2 | 0.0033 |
| 635 | 1430552_a_at | Sbf1 | SET binding factor 1 | - | 1.3 | 0.0014 |
| 636 | 1434707_at | Sbf1 | SET binding factor 1 | - | 1.3 | 0.0039 |
| 637 | 1424709_at | Sc5d | sterol-C5-desaturase (fungal ERG3, delta-5-desaturase) homolog (S. cerevisae) | - | 1.3 | 0.0010 |
| 638 | 1434908_at | Scaf1 | SR-related CTD-associated factor 1 | - | 1.2 | 0.0034 |
| 639 | 1448404_at | Scamp2 | secretory carrier membrane protein 2 | - | 1.2 | 0.0085 |
| 640 | 1415823_at | Scd2 | stearoyl-Coenzyme A desaturase 2 | - | 1.4 | 0.0054 |
| 641 | 1436646_at | Scn2a1 | sodium channel, voltage-gated, type II, alpha 1 | + | 1.4 | 0.0011 |
| 642 | 1431760_a_at | Sdccag3 | serologically defined colon cancer antigen 3 | - | 1.3 | 0.0025 |
| 643 | 1428695_at | Sdr39u1 | short chain dehydrogenase/reductase family 39U, member 1 | - | 1.3 | 0.0064 |
| 644 | 1451908_a_at | Sec14l1 | SEC14-like 1 (S. cerevisiae) | - | 1.2 | 0.0083 |
| 645 | 1453014_a_at | Sec31a | Sec31 homolog A (S. cerevisiae) | - | 1.2 | 0.0086 |
| 646 | 1416190_a_at | Sec61a1 | Sec61 alpha 1 subunit (S. cerevisiae) | - | 1.3 | 0.0018 |
| 647 | 1459109_at | Sema5a | sema domain, seven thrombospondin repeats (type 1 and type 1-like), transmembrane domain (TM) and short cytoplasmic domain, (semaphorin) 5A | + | 1.2 | 0.0080 |
| 648 | 1415828_a_at | Serp1 | stress-associated endoplasmic reticulum protein 1 | - | 1.4 | 0.0011 |
| 649 | 1416318_at | Serpinb1a | serine (or cysteine) peptidase inhibitor, clade B, member 1a | - | 1.3 | 0.0035 |
| 650 | 1422776_at | Serpinb8 | serine (or cysteine) peptidase inhibitor, clade B, member 8 | - | 1.3 | 0.0070 |
| 651 | 1420885_a_at | Sez6 | seizure related gene 6 | - | 1.3 | 0.0005 |
| 652 | 1456040_at | Sf3b2 | splicing factor 3b, subunit 2 | - | 1.2 | 0.0015 |
| 653 | 1416594_at | Sfrp1 | secreted frizzled-related protein 1 | + | 1.4 | 0.0082 |
| 654 | 1460187_at | Sfrp1 | secreted frizzled-related protein 1 | - | 1.3 | 0.0082 |
| 655 | 1449228_at | Sh3gl2 | SH3-domain GRB2-like 2 | - | 1.4 | 0.0043 |
| 656 | 1451267_at | Sharpin | SHANK-associated RH domain interacting protein | - | 1.2 | 0.0060 |
| 657 | 1424824_at | Slain1 | SLAIN motif family, member 1 | - | 1.2 | 0.0056 |
| 658 | 1445305_at | Slc10a7 | solute carrier family 10 (sodium/bile acid cotransporter family), member 7 | + | 1.3 | 0.0055 |
| 659 | 1436989_s_at | Slc12a6 | solute carrier family 12, member 6 | + | 1.2 | 0.0036 |
| 660 | 1422897_at | Slc22a12 | solute carrier family 22 (organic anion/cation transporter), member 12 | - | 1.4 | 0.0046 |
| 661 | 1416966_at | Slc22a8 | solute carrier family 22 (organic anion transporter), member 8 | - | 1.3 | 0.0091 |
| 662 | 1428190_at | Slc25a1 | solute carrier family 25 (mitochondrial carrier, citrate transporter), member 1 | - | 1.2 | 0.0020 |
| 663 | 1420966_at | Slc25a15 | solute carrier family 25 (mitochondrial carrier ornithine transporter), member 15 | + | 1.2 | 0.0033 |
| 664 | 1453111_a_at | Slc25a39 | solute carrier family 25, member 39 | - | 1.2 | 0.0099 |
| 665 | 1422811_at | Slc27a1 | solute carrier family 27 (fatty acid transporter), member 1 | - | 1.2 | 0.0095 |
| 666 | 1421924_at | Slc2a3 | solute carrier family 2 (facilitated glucose transporter), member 3 | - | 1.2 | 0.0053 |
| 667 | 1419972_at | Slc35a5 | solute carrier family 35, member A5 | - | 1.3 | 0.0071 |
| 668 | 1447248_at | Slc39a12 | solute carrier family 39 (zinc transporter), member 12 | - | 1.4 | 0.0053 |
| 669 | 1438490_at | Slc39a14 | solute carrier family 39 (zinc transporter), member 14 | - | 1.2 | 0.0098 |
| 670 | 1458394_at | Slc6a17 | solute carrier family 6 (neurotransmitter transporter), member 17 | - | 1.2 | 0.0078 |
| 671 | 1417150_at | Slc6a4 | solute carrier family 6 (neurotransmitter transporter, serotonin), member 4 | + | 10.6 | 0.0000 |
| 672 | 1439368_a_at | Slc9a3r2 | solute carrier family 9 (sodium/hydrogen exchanger), member 3 regulator 2 | - | 1.2 | 0.0066 |
| 673 | 1439369_x_at | Slc9a3r2 | solute carrier family 9 (sodium/hydrogen exchanger), member 3 regulator 2 | - | 1.2 | 0.0034 |
| 674 | 1431449_at | Slco6d1 | solute carrier organic anion transporter family, member 6d1 | + | 1.3 | 0.0021 |
| 675 | 1421215_a_at | Slmap | sarcolemma associated protein | - | 1.3 | 0.0067 |
| 676 | 1420030_at | Slu7 | SLU7 splicing factor homolog (S. cerevisiae) | + | 1.3 | 0.0058 |
| 677 | 1423077_at | Snx9 | sorting nexin 9 | - | 1.2 | 0.0094 |
| 678 | 1419329_at | Sorbs3 | sorbin and SH3 domain containing 3 | - | 1.3 | 0.0068 |
| 679 | 1428338_at | Spata2L | spermatogenesis associated 2-like | - | 1.3 | 0.0049 |
| 680 | 1450618_a_at | Sprr2a | small proline-rich protein 2A | + | 1.3 | 0.0080 |
| 681 | 1422401_at | Sprr3 | small proline-rich protein 3 | + | 1.2 | 0.0091 |
| 682 | 1428472_at | Spsb1 | splA/ryanodine receptor domain and SOCS box containing 1 | - | 1.2 | 0.0040 |
| 683 | 1426744_at | Srebf2 | sterol regulatory element binding factor 2 | - | 1.2 | 0.0082 |
| 684 | 1425351_at | Srxn1 | sulfiredoxin 1 homolog (S. cerevisiae) | - | 1.3 | 0.0044 |
| 685 | 1421890_at | St3gal2 | ST3 beta-galactoside alpha-2,3-sialyltransferase 2 | - | 1.3 | 0.0020 |
| 686 | 1419550_a_at | Stk39 | serine/threonine kinase 39, STE20/SPS1 homolog (yeast) | - | 1.4 | 0.0027 |
| 687 | 1419913_at | Strap | serine/threonine kinase receptor associated protein | + | 1.5 | 0.0057 |
| 688 | 1422036_at | Strn | striatin, calmodulin binding protein | + | 1.3 | 0.0007 |
| 689 | 1426343_at | Stt3b | STT3, subunit of the oligosaccharyltransferase complex, homolog B (S. cerevisiae) | - | 1.2 | 0.0093 |
| 690 | 1421606_a_at | Sult4a1 | sulfotransferase family 4A, member 1 | - | 1.3 | 0.0058 |
| 691 | 1453049_at | Svip | small VCP/p97-interacting protein | - | 1.2 | 0.0046 |
| 692 | 1428685_at | Syce1 | synaptonemal complex central element protein 1 | + | 1.3 | 0.0016 |
| 693 | 1448216_at | Syngr3 | synaptogyrin 3 | - | 1.3 | 0.0024 |
| 694 | 1417708_at | Syt3 | synaptotagmin III | - | 1.3 | 0.0077 |
| 695 | 1422531_at | Syt5 | synaptotagmin V | - | 1.4 | 0.0013 |
| 696 | 1424169_at | Tax1bp3 | Tax1 (human T-cell leukemia virus type I) binding protein 3 | - | 1.5 | 0.0001 |
| 697 | 1415750_at | Tbl3 | transducin (beta)-like 3 | - | 1.2 | 0.0030 |
| 698 | 1425158_at | Tbx20 | T-box 20 | + | 1.2 | 0.0085 |
| 699 | 1424531_a_at | Tcea3 | transcription elongation factor A (SII), 3 | - | 1.3 | 0.0078 |
| 700 | 1448981_x_at | Tcl1b1 | T-cell leukemia/lymphoma 1B, 1 | + | 1.6 | 0.0030 |
| 701 | 1423600_a_at | Tcof1 | Treacher Collins Franceschetti syndrome 1, homolog | - | 1.2 | 0.0075 |
| 702 | 1420729_at | Tcstv1 | 2-cell-stage, variable group, member 1 | + | 1.3 | 0.0021 |
| 703 | 1421513_at | Tdrd1 | tudor domain containing 1 | + | 1.3 | 0.0041 |
| 704 | 1418064_at | Tfpt | TCF3 (E2A) fusion partner | - | 1.2 | 0.0071 |
| 705 | 1428425_at | Tgfbrap1 | transforming growth factor, beta receptor associated protein 1 | - | 1.2 | 0.0079 |
| 706 | 1418765_at | Timd2 | T-cell immunoglobulin and mucin domain containing 2 | + | 1.2 | 0.0074 |
| 707 | 1448518_at | Timm22 | translocase of inner mitochondrial membrane 22 homolog (yeast) | + | 1.2 | 0.0028 |
| 708 | 1432282_a_at | Tlcd2 | TLC domain containing 2 | + | 1.3 | 0.0077 |
| 709 | 1448375_at | Tm9sf3 | transmembrane 9 superfamily member 3 | - | 1.2 | 0.0039 |
| 710 | 1458372_at | Tmc8 | transmembrane channel-like gene family 8 | + | 1.2 | 0.0053 |
| 711 | 1451546_s_at | Tmem40 | transmembrane protein 40 | - | 1.2 | 0.0061 |
| 712 | 1423871_at | Tmem63a | transmembrane protein 63a | - | 1.2 | 0.0054 |
| 713 | 1453285_at | Tmem88 | transmembrane protein 88 | - | 1.3 | 0.0014 |
| 714 | 1424133_at | Tmem98 | transmembrane protein 98 | - | 1.5 | 0.0084 |
| 715 | 1423048_a_at | Tollip | toll interacting protein | - | 1.3 | 0.0047 |
| 716 | 1431188_a_at | Tom1 | target of myb1 homolog (chicken) | - | 1.2 | 0.0032 |
| 717 | 1436008_at | Tpd52 | tumor protein D52 | + | 1.3 | 0.0099 |
| 718 | 1449997_at | Tpm3 | tropomyosin 3, gamma | - | 1.2 | 0.0052 |
| 719 | 1427567_a_at | Tpm3 | tropomyosin 3, gamma | - | 1.2 | 0.0094 |
| 720 | 1439043_at | Tra2a | transformer 2 alpha homolog (Drosophila) | + | 1.6 | 0.0031 |
| 721 | 1425546_a_at | Trf | transferrin | - | 1.3 | 0.0026 |
| 722 | 1451663_a_at | Trim3 | tripartite motif-containing 3 | - | 1.2 | 0.0082 |
| 723 | 1425621_at | Trim35 | tripartite motif-containing 35 | - | 1.4 | 0.0005 |
| 724 | 1437314_a_at | Trmt1 | TRM1 tRNA methyltransferase 1 homolog (S. cerevisiae) | - | 1.2 | 0.0050 |
| 725 | 1427739_a_at | Trp53 | transformation related protein 53 | - | 1.3 | 0.0097 |
| 726 | 1452325_at | Trp73 | transformation related protein 73 | - | 1.2 | 0.0076 |
| 727 | 1418783_at | Trpm5 | transient receptor potential cation channel, subfamily M, member 5 | + | 1.3 | 0.0074 |
| 728 | 1443392_at | Trpv1 | transient receptor potential cation channel, subfamily V, member 1 | + | 1.6 | 0.0000 |
| 729 | 1417502_at | Tspan7 | tetraspanin 7 | - | 1.3 | 0.0073 |
| 730 | 1455414_at | Ttc16 | tetratricopeptide repeat domain 16 | - | 1.2 | 0.0031 |
| 731 | 1435456_at | Ttc28 | tetratricopeptide repeat domain 28 | - | 1.2 | 0.0071 |
| 732 | 1433460_at | Ttc7b | tetratricopeptide repeat domain 7B | - | 1.3 | 0.0044 |
| 733 | 1443948_at | Ttc9c | tetratricopeptide repeat domain 9C | - | 1.2 | 0.0095 |
| 734 | 1451694_at | Ttll3 | tubulin tyrosine ligase-like family, member 3 | + | 1.4 | 0.0011 |
| 735 | 1423221_at | Tubb4 | tubulin, beta 4 | - | 1.2 | 0.0099 |
| 736 | 1423035_s_at | Txndc17 | thioredoxin domain containing 17 | - | 1.2 | 0.0068 |
| 737 | 1423036_at | Txndc17 | thioredoxin domain containing 17 | - | 1.3 | 0.0018 |
| 738 | 1439302_at | Uba6 | ubiquitin-like modifier activating enzyme 6 | + | 1.2 | 0.0096 |
| 739 | 1448017_at | Ubap2l | ubiquitin associated protein 2-like | - | 1.2 | 0.0085 |
| 740 | 1430177_at | Ube2b | ubiquitin-conjugating enzyme E2B, RAD6 homology (S. cerevisiae) | - | 1.4 | 0.0001 |
| 741 | 1424062_at | Ube2d1 | ubiquitin-conjugating enzyme E2D 1, UBC4/5 homolog (yeast) | - | 1.2 | 0.0070 |
| 742 | 1448692_at | Ubqln4 | ubiquilin 4 | - | 1.2 | 0.0049 |
| 743 | 1435110_at | Unc5b | unc-5 homolog B (C. elegans) | - | 1.2 | 0.0036 |
| 744 | 1420833_at | Vamp2 | vesicle-associated membrane protein 2 | - | 1.4 | 0.0050 |
| 745 | 1421102_a_at | Vamp3 | vesicle-associated membrane protein 3 | - | 1.4 | 0.0006 |
| 746 | 1417123_at | Vav3 | vav 3 oncogene | - | 1.3 | 0.0012 |
| 747 | 1420909_at | Vegfa | vascular endothelial growth factor A | - | 1.2 | 0.0037 |
| 748 | 1425490_a_at | Wdr13 | WD repeat domain 13 | - | 1.3 | 0.0049 |
| 749 | 1432217_a_at | Wdr16 | WD repeat domain 16 | - | 1.2 | 0.0095 |
| 750 | 1429552_at | Wdr16 | WD repeat domain 16 | - | 1.7 | 0.0007 |
| 751 | 1455278_at | Wdr37 | WD repeat domain 37 | + | 1.2 | 0.0051 |
| 752 | 1460012_at | Wfdc3 | WAP four-disulfide core domain 3 | + | 1.3 | 0.0074 |
| 753 | 1453737_at | Wipf2 | WAS/WASL interacting protein family, member 2 | - | 1.3 | 0.0093 |
| 754 | 1444050_at | Wisp3 | WNT1 inducible signaling pathway protein 3 | - | 1.2 | 0.0096 |
| 755 | 1431942_at | Wwox | WW domain-containing oxidoreductase | + | 1.4 | 0.0006 |
| 756 | 1443621_at | Xaf1 | XIAP associated factor 1 | + | 7.6 | 0.0000 |
| 757 | 1443698_at | Xaf1 | XIAP associated factor 1 | - | 1.7 | 0.0000 |
| 758 | 1420012_at | Xbp1 | X-box binding protein 1 | + | 1.4 | 0.0035 |
| 759 | 1422532_at | Xpc | xeroderma pigmentosum, complementation group C | - | 1.2 | 0.0076 |
| 760 | 1439005_x_at | Ywhaz | tyrosine 3-monooxygenase/tryptophan 5-monooxygenase activation protein, zeta polypeptide | + | 1.3 | 0.0092 |
| 761 | 1448218_s_at | Ywhaz | tyrosine 3-monooxygenase/tryptophan 5-monooxygenase activation protein, zeta polypeptide | - | 1.2 | 0.0043 |
| 762 | 1419899_at | Zc3h7a | zinc finger CCCH type containing 7 A | + | 1.3 | 0.0014 |
| 763 | 1419955_at | Zfand3 | zinc finger, AN1-type domain 3 | + | 1.2 | 0.0081 |
| 764 | 1425097_a_at | Zfp106 | zinc finger protein 106 | - | 1.2 | 0.0046 |
| 765 | 1439698_at | Zfp276 | zinc finger protein (C2H2 type) 276 | + | 1.3 | 0.0051 |
| 766 | 1452519_a_at | Zfp36 | zinc finger protein 36 | - | 1.2 | 0.0043 |
| 767 | 1426563_at | Zfp553 | zinc finger protein 553 | - | 1.3 | 0.0030 |
| 768 | 1436429_at | Zfp606 | zinc finger protein 606 | - | 1.4 | 0.0018 |
| 769 | 1449691_at | Zfp644 | zinc finger protein 644 | + | 1.3 | 0.0017 |
| 770 | 1436020_at | Zfp828 | zinc finger protein 828 | - | 1.3 | 0.0075 |
| 771 | 1449552_at | Zfr | zinc finger RNA binding protein | - | 1.4 | 0.0082 |
| 772 | 1438787_at | Zscan10 | zinc finger and SCAN domain containing 10 | + | 1.2 | 0.0092 |
| 773 | 1438691_at | Zzef1 | zinc finger, ZZ-type with EF hand domain 1 | - | 1.9 | 0.0000 |
