# Supplementary material for: Differential Effects of Prenatal Stress in 5-Htt Deficient Mice: Towards Molecular Mechanisms of Gene × Environment Interactions
Source: PLoS One. 2011 Aug 12;6(8):e22715. doi: 10.1371/journal.pone.0022715 (PMC3155516; doi:10.1371/journal.pone.0022715)
Supplement: Text S2 — (DOC) [file pone.0022715.s002.doc]

**Supplemental material 2**

**Genes affected by the environment (prenatal stress versus control)**.

ID: Affymetrix ID; EFFECT: direction of effect; “+”: gene expression upregulated in prenatally stressed versus control mice; “-“: downregulated in prenatally stressed versus control mice; FC: fold-change in mRNA expression. Genes have been ordered alphabetically.

| **#** | **ID** | **SYMBOL** | **GENENAME** | **Effect** | **FC** | **P.Value** |
| --- | --- | --- | --- | --- | --- | --- |
| 1 | 1452437_at | 1110021J02Rik | RIKEN cDNA 1110021J02 gene | + | 1.5 | 0.0007 |
| 2 | 1437981_x_at | 1110057K04Rik | RIKEN cDNA 1110057K04 gene | + | 1.2 | 0.0016 |
| 3 | 1428922_at | 1200009O22Rik | RIKEN cDNA 1200009O22 gene | - | 1.2 | 0.0013 |
| 4 | 1451082_at | 1300018I05Rik | RIKEN cDNA 1300018I05 gene | - | 1.2 | 0.0091 |
| 5 | 1447537_at | 1500032P08Rik | RIKEN cDNA 1500032P08 gene | + | 1.2 | 0.0092 |
| 6 | 1430261_at | 1700024J04Rik | RIKEN cDNA 1700024J04 gene | + | 1.1 | 0.0090 |
| 7 | 1432116_at | 1700031F05Rik | RIKEN cDNA 1700031F05 gene | + | 1.3 | 0.0058 |
| 8 | 1441406_at | 1700052N19Rik | RIKEN cDNA 1700052N19 gene | + | 1.2 | 0.0044 |
| 9 | 1459809_x_at | 1700063D05Rik | RIKEN cDNA 1700063D05 gene | + | 1.3 | 0.0032 |
| 10 | 1442779_at | 1700071K01Rik | RIKEN cDNA 1700071K01 gene | - | 1.3 | 0.0031 |
| 11 | 1420681_at | 1700120K04Rik | RIKEN cDNA 1700120K04 gene | + | 1.2 | 0.0063 |
| 12 | 1447594_at | 1810012K16Rik | RIKEN cDNA 1810012K16 gene | - | 1.2 | 0.0085 |
| 13 | 1430991_at | 1810014B01Rik | RIKEN cDNA 1810014B01 gene | + | 1.5 | 0.0005 |
| 14 | 1439561_at | 2010012O05Rik | RIKEN cDNA 2010012O05 gene | - | 1.1 | 0.0074 |
| 15 | 1429895_at | 2310010G23Rik | RIKEN cDNA 2310010G23 gene | - | 1.2 | 0.0033 |
| 16 | 1432107_at | 2310010M20Rik | RIKEN cDNA 2310010M20 gene | - | 1.2 | 0.0094 |
| 17 | 1430446_at | 2310057B04Rik | RIKEN cDNA 2310057B04 gene | - | 1.2 | 0.0036 |
| 18 | 1460410_at | 2310079F23Rik | RIKEN cDNA 2310079F23 gene | + | 1.2 | 0.0088 |
| 19 | 1435377_at | 2410002O22Rik | RIKEN cDNA 2410002O22 gene | + | 1.2 | 0.0034 |
| 20 | 1432500_at | 2410006F04Rik | RIKEN cDNA 2410006F04 gene | - | 1.2 | 0.0095 |
| 21 | 1430845_at | 2410018L13Rik | RIKEN cDNA 2410018L13 gene | - | 1.2 | 0.0036 |
| 22 | 1420114_s_at | 2410022L05Rik | RIKEN cDNA 2410022L05 gene | + | 1.4 | 0.0039 |
| 23 | 1429245_at | 2510022D24Rik | RIKEN cDNA 2510022D24 gene | - | 1.2 | 0.0068 |
| 24 | 1453966_at | 2610008G14Rik | RIKEN cDNA 2610008G14 gene | + | 1.2 | 0.0073 |
| 25 | 1432606_at | 2610012C04Rik | RIKEN cDNA 2610012C04 gene | + | 1.2 | 0.0020 |
| 26 | 1452979_at | 2610110G12Rik | RIKEN cDNA 2610110G12 gene | - | 1.3 | 0.0005 |
| 27 | 1428624_at | 2810482I07Rik | RIKEN cDNA 2810482I07 gene | + | 1.2 | 0.0062 |
| 28 | 1433387_at | 2900022M07Rik | RIKEN cDNA 2900022M07 gene | + | 1.4 | 0.0018 |
| 29 | 1420820_at | 2900073G15Rik | RIKEN cDNA 2900073G15 gene | + | 1.2 | 0.0051 |
| 30 | 1430478_at | 3110043A19Rik | RIKEN cDNA 3110043A19 gene | - | 1.3 | 0.0068 |
| 31 | 1430841_at | 3300002P13Rik | RIKEN cDNA 3300002P13 gene | + | 1.2 | 0.0091 |
| 32 | 1430858_at | 4632433K11Rik | RIKEN cDNA 4632433K11 gene | - | 1.2 | 0.0097 |
| 33 | 1430382_at | 4833413G10Rik | RIKEN cDNA 4833413G10 gene | - | 1.2 | 0.0007 |
| 34 | 1436524_at | 4833438C02Rik | RIKEN cDNA 4833438C02 gene | - | 1.3 | 0.0005 |
| 35 | 1423381_at | 4921521F21Rik | RIKEN cDNA 4921521F21 gene | + | 1.3 | 0.0009 |
| 36 | 1453650_at | 4930413F20Rik | RIKEN cDNA 4930413F20 gene | - | 1.2 | 0.0095 |
| 37 | 1440349_at | 4930420K17Rik | RIKEN cDNA 4930420K17 gene | + | 1.1 | 0.0071 |
| 38 | 1433082_at | 4930448K20Rik | glyceraldehyde-3-phosphate dehydrogenase pseudogene | + | 1.6 | 0.0040 |
| 39 | 1429353_a_at | 4930511M11Rik | RIKEN cDNA 4930511M11 gene | + | 1.2 | 0.0034 |
| 40 | 1432223_at | 4930552N02Rik | RIKEN cDNA 4930552N02 gene | + | 1.2 | 0.0002 |
| 41 | 1428375_at | 4932415G12Rik | RIKEN cDNA 4932415G12 gene | - | 1.2 | 0.0024 |
| 42 | 1453828_at | 4932422M17Rik | RIKEN cDNA 4932422M17 gene | + | 1.2 | 0.0031 |
| 43 | 1432882_at | 4932431P20Rik | RIKEN cDNA 4932431P20 gene | + | 1.4 | 0.0020 |
| 44 | 1432127_at | 4933401L05Rik | RIKEN cDNA 4933401L05 gene | + | 1.2 | 0.0096 |
| 45 | 1454101_at | 4933401P06Rik | RIKEN cDNA 4933401P06 gene | + | 1.2 | 0.0077 |
| 46 | 1431853_at | 4933413C19Rik | RIKEN cDNA 4933413C19 gene | + | 1.2 | 0.0059 |
| 47 | 1432197_at | 4933417D19Rik | RIKEN cDNA 4933417D19 gene | + | 1.2 | 0.0048 |
| 48 | 1427065_at | 4933439F18Rik | RIKEN cDNA 4933439F18 gene | + | 1.2 | 0.0084 |
| 49 | 1427068_x_at | 4933439F18Rik | RIKEN cDNA 4933439F18 gene | + | 1.2 | 0.0010 |
| 50 | 1432935_at | 5330433J24Rik | RIKEN cDNA 5330433J24 gene | + | 1.3 | 0.0082 |
| 51 | 1432621_at | 5730410E19Rik | RIKEN cDNA 5730410E19 gene | + | 1.2 | 0.0040 |
| 52 | 1447774_x_at | 5730469M10Rik | RIKEN cDNA 5730469M10 gene | + | 1.2 | 0.0063 |
| 53 | 1453079_at | 5730494M16Rik | RIKEN cDNA 5730494M16 gene | - | 1.3 | 0.0067 |
| 54 | 1431528_at | 5830427D02Rik | RIKEN cDNA 5830427D02 gene | + | 1.2 | 0.0004 |
| 55 | 1432998_at | 5830462O15Rik | RIKEN cDNA 5830462O15 gene | - | 1.3 | 0.0021 |
| 56 | 1432990_at | 5830495A06Rik | RIKEN cDNA 5830495A06 gene | + | 1.2 | 0.0028 |
| 57 | 1446341_at | 6030403N03Rik | RIKEN cDNA 6030403N03 gene | + | 1.2 | 0.0088 |
| 58 | 1460522_at | 6030458E02Rik | RIKEN cDNA 6030458E02 gene | + | 1.2 | 0.0067 |
| 59 | 1437753_at | 6230409E13Rik | RIKEN cDNA 6230409E13 gene | + | 1.1 | 0.0076 |
| 60 | 1456765_at | 6430511F03 | hypothetical protein 6430511F03 | + | 1.1 | 0.0093 |
| 61 | 1452082_at | 6430548M08Rik | RIKEN cDNA 6430548M08 gene | - | 1.2 | 0.0006 |
| 62 | 1459051_at | 6530418L21Rik | RIKEN cDNA 6530418L21 gene | + | 1.2 | 0.0089 |
| 63 | 1444797_at | 8030474K03Rik | RIKEN cDNA 8030474K03 gene | + | 1.3 | 0.0009 |
| 64 | 1454056_at | 9030407P20Rik | RIKEN cDNA 9030407P20 gene | - | 1.2 | 0.0098 |
| 65 | 1433101_at | 9030419F21Rik | RIKEN cDNA 9030419F21 gene | + | 1.2 | 0.0014 |
| 66 | 1424555_at | 9430015G10Rik | RIKEN cDNA 9430015G10 gene | - | 1.2 | 0.0023 |
| 67 | 1435159_at | 9430038I01Rik | RIKEN cDNA 9430038I01 gene | - | 1.2 | 0.0058 |
| 68 | 1445167_at | 9630001P10Rik | RIKEN cDNA 9630001P10 gene | - | 1.3 | 0.0014 |
| 69 | 1456634_at | 9830001H06Rik | RIKEN cDNA 9830001H06 gene | - | 1.2 | 0.0057 |
| 70 | 1433577_at | A730017C20Rik | RIKEN cDNA A730017C20 gene | - | 1.2 | 0.0061 |
| 71 | 1456167_at | AA619741 | expressed sequence AA619741 | - | 1.2 | 0.0055 |
| 72 | 1451377_a_at | Aaas | achalasia, adrenocortical insufficiency, alacrimia | - | 1.2 | 0.0002 |
| 73 | 1419931_at | Abcb7 | ATP-binding cassette, sub-family B (MDR/TAP), member 7 | + | 1.3 | 0.0023 |
| 74 | 1423863_at | Abcf2 | ATP-binding cassette, sub-family F (GCN20), member 2 | - | 1.2 | 0.0042 |
| 75 | 1449827_at | Acan | aggrecan | - | 1.2 | 0.0087 |
| 76 | 1422635_at | Ache | acetylcholinesterase | - | 1.3 | 0.0008 |
| 77 | 1451002_at | Aco2 | aconitase 2, mitochondrial | - | 1.2 | 0.0084 |
| 78 | 1425257_at | Acot5 | acyl-CoA thioesterase 5 | + | 1.2 | 0.0044 |
| 79 | 1418073_at | Acot9 | acyl-CoA thioesterase 9 | - | 1.2 | 0.0030 |
| 80 | 1419734_at | Actb | actin, beta | - | 1.2 | 0.0009 |
| 81 | 1422564_at | Actl6b | actin-like 6B | - | 1.2 | 0.0082 |
| 82 | 1448327_at | Actn2 | actinin alpha 2 | - | 1.2 | 0.0053 |
| 83 | 1452057_at | Actr1b | ARP1 actin-related protein 1 homolog B, centractin beta (yeast) | - | 1.2 | 0.0041 |
| 84 | 1448460_at | Acvr1 | activin A receptor, type 1 | + | 1.1 | 0.0070 |
| 85 | 1416080_at | Adam15 | a disintegrin and metallopeptidase domain 15 (metargidin) | - | 1.2 | 0.0055 |
| 86 | 1458197_x_at | Adam1a | a disintegrin and metallopeptidase domain 1a | + | 1.2 | 0.0012 |
| 87 | 1443378_s_at | Adam1a | a disintegrin and metallopeptidase domain 1a | + | 1.3 | 0.0013 |
| 88 | 1427790_at | Adam1a | a disintegrin and metallopeptidase domain 1a | + | 1.4 | 0.0003 |
| 89 | 1416871_at | Adam8 | a disintegrin and metallopeptidase domain 8 | + | 1.3 | 0.0001 |
| 90 | 1444253_at | Adamts18 | a disintegrin-like and metallopeptidase (reprolysin type) with thrombospondin type 1 motif, 18 | - | 1.3 | 0.0091 |
| 91 | 1455965_at | Adamts4 | a disintegrin-like and metallopeptidase (reprolysin type) with thrombospondin type 1 motif, 4 | + | 1.3 | 0.0009 |
| 92 | 1425638_at | Adap2 | ArfGAP with dual PH domains 2 | - | 1.3 | 0.0011 |
| 93 | 1448490_at | Adck4 | aarF domain containing kinase 4 | - | 1.2 | 0.0014 |
| 94 | 1420970_at | Adcy7 | adenylate cyclase 7 | + | 1.1 | 0.0060 |
| 95 | 1430482_at | Adora3 | adenosine A3 receptor | + | 1.3 | 0.0012 |
| 96 | 1438158_at | Agbl4 | ATP/GTP binding protein-like 4 | - | 1.2 | 0.0043 |
| 97 | 1425362_at | Agfg2 | ArfGAP with FG repeats 2 | + | 1.2 | 0.0009 |
| 98 | 1440138_at | Ahdc1 | AT hook, DNA binding motif, containing 1 | + | 1.3 | 0.0015 |
| 99 | 1444026_at | AI593442 | expressed sequence AI593442 | + | 1.2 | 0.0082 |
| 100 | 1416911_a_at | Akirin1 | akirin 1 | + | 1.2 | 0.0059 |
| 101 | 1448143_at | Aldh2 | aldehyde dehydrogenase 2, mitochondrial | + | 1.2 | 0.0028 |
| 102 | 1434987_at | Aldh2 | aldehyde dehydrogenase 2, mitochondrial | + | 1.3 | 0.0001 |
| 103 | 1434988_x_at | Aldh2 | aldehyde dehydrogenase 2, mitochondrial | + | 1.3 | 0.0001 |
| 104 | 1438941_x_at | Ampd2 | adenosine monophosphate deaminase 2 (isoform L) | + | 1.2 | 0.0018 |
| 105 | 1436967_at | Ankrd11 | ankyrin repeat domain 11 | - | 1.2 | 0.0071 |
| 106 | 1433543_at | Anln | anillin, actin binding protein | + | 1.2 | 0.0062 |
| 107 | 1437895_at | Ano8 | anoctamin 8 | - | 1.2 | 0.0059 |
| 108 | 1415818_at | Anxa6 | annexin A6 | - | 1.3 | 0.0029 |
| 109 | 1416134_at | Aplp1 | amyloid beta (A4) precursor-like protein 1 | - | 1.4 | 0.0066 |
| 110 | 1421889_a_at | Aplp2 | amyloid beta (A4) precursor-like protein 2 | - | 1.2 | 0.0069 |
| 111 | 1418849_x_at | Aqp7 | aquaporin 7 | + | 1.3 | 0.0042 |
| 112 | 1434074_x_at | Arf4 | ADP-ribosylation factor 4 | + | 1.3 | 0.0089 |
| 113 | 1448672_a_at | Arfgap2 | ADP-ribosylation factor GTPase activating protein 2 | - | 1.2 | 0.0041 |
| 114 | 1431133_at | Arhgap18 | Rho GTPase activating protein 18 | + | 1.1 | 0.0084 |
| 115 | 1451320_at | Arhgap8 | Rho GTPase activating protein 8 | + | 1.2 | 0.0049 |
| 116 | 1421164_a_at | Arhgef1 | Rho guanine nucleotide exchange factor (GEF) 1 | - | 1.2 | 0.0023 |
| 117 | 1418523_at | Arih2 | ariadne homolog 2 (Drosophila) | - | 1.3 | 0.0006 |
| 118 | 1424020_at | Arl6ip6 | ADP-ribosylation factor-like 6 interacting protein 6 | + | 1.2 | 0.0018 |
| 119 | 1428399_a_at | Armc9 | armadillo repeat containing 9 | - | 1.2 | 0.0099 |
| 120 | 1460703_at | Ascc1 | activating signal cointegrator 1 complex subunit 1 | - | 1.2 | 0.0097 |
| 121 | 1418292_at | Asna1 | arsA arsenite transporter, ATP-binding, homolog 1 (bacterial) | - | 1.4 | 0.0010 |
| 122 | 1430656_a_at | Asnsd1 | asparagine synthetase domain containing 1 | - | 1.1 | 0.0093 |
| 123 | 1438992_x_at | Atf4 | activating transcription factor 4 | + | 1.3 | 0.0001 |
| 124 | 1439457_x_at | Atg12 | autophagy-related 12 (yeast) | + | 1.2 | 0.0026 |
| 125 | 1435594_at | Atl2 | atlastin GTPase 2 | - | 1.2 | 0.0057 |
| 126 | 1427481_a_at | Atp1a3 | ATPase, Na+/K+ transporting, alpha 3 polypeptide | - | 1.4 | 0.0004 |
| 127 | 1437157_at | Atp1b1 | ATPase, Na+/K+ transporting, beta 1 polypeptide | + | 1.3 | 0.0059 |
| 128 | 1437797_at | Atp2a2 | ATPase, Ca++ transporting, cardiac muscle, slow twitch 2 | + | 1.2 | 0.0032 |
| 129 | 1450124_a_at | Atp2a3 | ATPase, Ca++ transporting, ubiquitous | + | 1.2 | 0.0050 |
| 130 | 1444874_at | Atp5g1 | ATP synthase, H+ transporting, mitochondrial F0 complex, subunit c (subunit 9), isoform 1 | + | 1.2 | 0.0058 |
| 131 | 1437013_x_at | Atp6v0b | ATPase, H+ transporting, lysosomal V0 subunit B | - | 1.2 | 0.0041 |
| 132 | 1425448_x_at | Atp6v0b | ATPase, H+ transporting, lysosomal V0 subunit B | - | 1.2 | 0.0043 |
| 133 | 1448770_a_at | Atpif1 | ATPase inhibitory factor 1 | + | 1.3 | 0.0043 |
| 134 | 1438294_at | Atxn1 | ataxin 1 | + | 1.2 | 0.0089 |
| 135 | 1442081_at | AU017455 | expressed sequence AU017455 | - | 1.2 | 0.0094 |
| 136 | 1442403_at | B130034C11Rik | RIKEN cDNA B130034C11 gene | - | 1.3 | 0.0059 |
| 137 | 1456701_at | B230208H17Rik | RIKEN cDNA B230208H17 gene | + | 1.3 | 0.0044 |
| 138 | 1437433_at | B3galt2 | UDP-Gal:betaGlcNAc beta 1,3-galactosyltransferase, polypeptide 2 | - | 1.3 | 0.0007 |
| 139 | 1437644_at | B3galt2 | UDP-Gal:betaGlcNAc beta 1,3-galactosyltransferase, polypeptide 2 | - | 1.3 | 0.0067 |
| 140 | 1448941_at | B4galt2 | UDP-Gal:betaGlcNAc beta 1,4- galactosyltransferase, polypeptide 2 | - | 1.2 | 0.0073 |
| 141 | 1427230_at | B930041F14Rik | RIKEN cDNA B930041F14 gene | - | 1.2 | 0.0032 |
| 142 | 1425656_a_at | Baiap2 | brain-specific angiogenesis inhibitor 1-associated protein 2 | - | 1.2 | 0.0090 |
| 143 | 1448221_at | Bat1a | HLA-B-associated transcript 1A | - | 1.2 | 0.0016 |
| 144 | 1423315_at | Bbc3 | BCL2 binding component 3 | + | 1.2 | 0.0071 |
| 145 | 1460369_at | BC003267 | cDNA sequence BC003267 | - | 1.2 | 0.0052 |
| 146 | 1425961_at | BC016548 | cDNA sequence BC016548 | + | 1.2 | 0.0096 |
| 147 | 1423919_at | BC023882 | cDNA sequence BC023882 | - | 1.2 | 0.0087 |
| 148 | 1424434_at | BC024814 | cDNA sequence BC024814 | - | 1.2 | 0.0080 |
| 149 | 1437186_at | BC055324 | cDNA sequence BC055324 | + | 1.2 | 0.0047 |
| 150 | 1444632_at | BC064078 | cDNA sequence BC064078 | - | 1.2 | 0.0043 |
| 151 | 1417077_at | Bcap29 | B-cell receptor-associated protein 29 | - | 1.2 | 0.0063 |
| 152 | 1443524_x_at | Bcl10 | B-cell leukemia/lymphoma 10 | + | 1.2 | 0.0032 |
| 153 | 1420887_a_at | Bcl2l1 | BCL2-like 1 | - | 1.3 | 0.0053 |
| 154 | 1450732_a_at | Bicd2 | bicaudal D homolog 2 (Drosophila) | + | 1.3 | 0.0003 |
| 155 | 1432410_a_at | Bmp7 | bone morphogenetic protein 7 | - | 1.2 | 0.0086 |
| 156 | 1449211_at | Bpnt1 | bisphosphate 3'-nucleotidase 1 | + | 1.1 | 0.0074 |
| 157 | 1447941_x_at | Braf | Braf transforming gene | - | 1.2 | 0.0061 |
| 158 | 1416738_at | Brap | BRCA1 associated protein | + | 1.3 | 0.0019 |
| 159 | 1426312_at | Bre | brain and reproductive organ-expressed protein | - | 1.2 | 0.0043 |
| 160 | 1429561_at | Brf2 | BRF2, subunit of RNA polymerase III transcription initiation factor, BRF1-like | - | 1.2 | 0.0054 |
| 161 | 1456616_a_at | Bsg | basigin | - | 1.3 | 0.0073 |
| 162 | 1424921_at | Bst2 | bone marrow stromal cell antigen 2 | + | 1.3 | 0.0051 |
| 163 | 1425884_at | Bxdc1 | brix domain containing 1 | + | 1.3 | 0.0021 |
| 164 | 1433931_at | C030046I01Rik | RIKEN cDNA C030046I01 gene | - | 1.3 | 0.0010 |
| 165 | 1433932_x_at | C030046I01Rik | RIKEN cDNA C030046I01 gene | - | 1.3 | 0.0003 |
| 166 | 1430501_at | C030047K22Rik | RIKEN cDNA C030047K22 gene | - | 1.2 | 0.0023 |
| 167 | 1442941_at | C77027 | expressed sequence C77027 | - | 1.2 | 0.0047 |
| 168 | 1460264_at | C81600 | expressed sequence C81600 | + | 1.2 | 0.0044 |
| 169 | 1417067_s_at | Cabc1 | chaperone, ABC1 activity of bc1 complex like (S. pombe) | + | 1.2 | 0.0049 |
| 170 | 1421255_a_at | Cabp1 | calcium binding protein 1 | - | 1.2 | 0.0091 |
| 171 | 1450520_at | Cacng3 | calcium channel, voltage-dependent, gamma subunit 3 | - | 1.3 | 0.0004 |
| 172 | 1435146_s_at | Cadm2 | cell adhesion molecule 2 | + | 1.2 | 0.0016 |
| 173 | 1456934_at | Calb1 | calbindin 1 | - | 1.2 | 0.0073 |
| 174 | 1449970_at | Capn12 | calpain 12 | + | 1.2 | 0.0069 |
| 175 | 1418671_at | Capn5 | calpain 5 | - | 1.2 | 0.0090 |
| 176 | 1434973_at | Car7 | carbonic anhydrase 7 | - | 1.2 | 0.0020 |
| 177 | 1452484_at | Car7 | carbonic anhydrase 7 | - | 1.2 | 0.0087 |
| 178 | 1436395_at | Card6 | caspase recruitment domain family, member 6 | - | 1.2 | 0.0076 |
| 179 | 1452394_at | Cars | cysteinyl-tRNA synthetase | - | 1.2 | 0.0099 |
| 180 | 1441615_at | Cbfa2t2 | core-binding factor, runt domain, alpha subunit 2, translocated to, 2 (human) | - | 1.2 | 0.0013 |
| 181 | 1427912_at | Cbr3 | carbonyl reductase 3 | + | 1.2 | 0.0028 |
| 182 | 1433861_at | Cc2d1b | coiled-coil and C2 domain containing 1B | - | 1.2 | 0.0016 |
| 183 | 1426097_a_at | Ccdc106 | coiled-coil domain containing 106 | - | 1.1 | 0.0078 |
| 184 | 1434313_at | Ccdc126 | coiled-coil domain containing 126 | + | 1.2 | 0.0083 |
| 185 | 1439538_at | Ccdc127 | coiled-coil domain containing 127 | - | 1.3 | 0.0008 |
| 186 | 1451799_at | Ccdc25 | coiled-coil domain containing 25 | + | 1.2 | 0.0008 |
| 187 | 1438569_at | Ccdc38 | coiled-coil domain containing 38 | + | 1.2 | 0.0046 |
| 188 | 1439109_at | Ccdc68 | coiled-coil domain containing 68 | - | 1.2 | 0.0039 |
| 189 | 1453824_at | Ccdc76 | coiled-coil domain containing 76 | + | 1.3 | 0.0056 |
| 190 | 1453609_s_at | Ccdc9 | coiled-coil domain containing 9 | - | 1.2 | 0.0087 |
| 191 | 1451395_at | Ccdc92 | coiled-coil domain containing 92 | - | 1.2 | 0.0074 |
| 192 | 1417266_at | Ccl6 | chemokine (C-C motif) ligand 6 | + | 1.2 | 0.0099 |
| 193 | 1417420_at | Ccnd1 | cyclin D1 | + | 1.2 | 0.0089 |
| 194 | 1448229_s_at | Ccnd2 | cyclin D2 | + | 1.2 | 0.0002 |
| 195 | 1430997_at | Cd47 | CD47 antigen (Rh-related antigen, integrin-associated signal transducer) | + | 1.2 | 0.0097 |
| 196 | 1448466_at | Cdca5 | cell division cycle associated 5 | - | 1.2 | 0.0063 |
| 197 | 1427095_at | Cdcp1 | CUB domain containing protein 1 | + | 1.4 | 0.0000 |
| 198 | 1418982_at | Cebpa | CCAAT/enhancer binding protein (C/EBP), alpha | - | 1.2 | 0.0013 |
| 199 | 1418901_at | Cebpb | CCAAT/enhancer binding protein (C/EBP), beta | + | 1.2 | 0.0008 |
| 200 | 1424996_at | Cflar | CASP8 and FADD-like apoptosis regulator | + | 1.2 | 0.0031 |
| 201 | 1424528_at | Cgref1 | cell growth regulator with EF hand domain 1 | - | 1.2 | 0.0016 |
| 202 | 1418816_at | Chmp1b | chromatin modifying protein 1B | + | 1.2 | 0.0009 |
| 203 | 1456722_at | Chrdl1 | chordin-like 1 | + | 1.2 | 0.0096 |
| 204 | 1448477_at | Chst12 | carbohydrate sulfotransferase 12 | - | 1.2 | 0.0044 |
| 205 | 1428977_at | Chst8 | carbohydrate (N-acetylgalactosamine 4-0) sulfotransferase 8 | - | 1.2 | 0.0066 |
| 206 | 1436391_s_at | Clcc1 | chloride channel CLIC-like 1 | - | 1.1 | 0.0088 |
| 207 | 1418283_at | Cldn4 | claudin 4 | + | 1.2 | 0.0046 |
| 208 | 1417839_at | Cldn5 | claudin 5 | + | 1.2 | 0.0093 |
| 209 | 1460039_at | Clec1a | C-type lectin domain family 1, member a | - | 1.2 | 0.0090 |
| 210 | 1427428_at | Clec4g | C-type lectin domain family 4, member g | + | 1.2 | 0.0053 |
| 211 | 1435256_at | Clip3 | CAP-GLY domain containing linker protein 3 | - | 1.2 | 0.0088 |
| 212 | 1457789_at | Cln3 | ceroid lipofuscinosis, neuronal 3, juvenile (Batten, Spielmeyer-Vogt disease) | + | 1.2 | 0.0034 |
| 213 | 1459992_x_at | Cln8 | ceroid-lipofuscinosis, neuronal 8 | + | 1.1 | 0.0088 |
| 214 | 1416541_at | Clpb | ClpB caseinolytic peptidase B homolog (E. coli) | - | 1.2 | 0.0026 |
| 215 | 1416883_at | Clptm1 | cleft lip and palate associated transmembrane protein 1 | - | 1.2 | 0.0033 |
| 216 | 1422158_at | Clstn2 | calsyntenin 2 | - | 1.2 | 0.0089 |
| 217 | 1453411_at | Cmc1 | COX assembly mitochondrial protein homolog (S. cerevisiae) | - | 1.2 | 0.0086 |
| 218 | 1423792_a_at | Cmtm6 | CKLF-like MARVEL transmembrane domain containing 6 | + | 1.2 | 0.0016 |
| 219 | 1422181_at | Cnga2 | cyclic nucleotide gated channel alpha 2 | + | 1.3 | 0.0008 |
| 220 | 1459783_s_at | Cno | cappuccino | + | 1.2 | 0.0047 |
| 221 | 1443829_x_at | Coasy | Coenzyme A synthase | + | 1.2 | 0.0055 |
| 222 | 1448081_at | Colq | collagen-like tail subunit (single strand of homotrimer) of asymmetric acetylcholinesterase | + | 1.2 | 0.0041 |
| 223 | 1428491_at | Commd10 | COMM domain containing 10 | - | 1.2 | 0.0060 |
| 224 | 1418701_at | Comt1 | catechol-O-methyltransferase 1 | - | 1.2 | 0.0077 |
| 225 | 1416017_at | Copg | coatomer protein complex, subunit gamma | - | 1.2 | 0.0084 |
| 226 | 1437039_at | Cops2 | COP9 (constitutive photomorphogenic) homolog, subunit 2 (Arabidopsis thaliana) | - | 1.3 | 0.0073 |
| 227 | 1423459_at | Cops2 | COP9 (constitutive photomorphogenic) homolog, subunit 2 (Arabidopsis thaliana) | - | 1.2 | 0.0054 |
| 228 | 1429078_a_at | Cops7a | COP9 (constitutive photomorphogenic) homolog, subunit 7a (Arabidopsis thaliana) | - | 1.2 | 0.0046 |
| 229 | 1437364_at | Coq3 | coenzyme Q3 homolog, methyltransferase (yeast) | - | 1.2 | 0.0014 |
| 230 | 1434755_at | Coro2b | coronin, actin binding protein, 2B | - | 1.3 | 0.0041 |
| 231 | 1417747_at | Cplx1 | complexin 1 | - | 1.2 | 0.0011 |
| 232 | 1436383_at | Cplx2 | complexin 2 | - | 1.2 | 0.0041 |
| 233 | 1449921_s_at | Cpne6 | copine VI | - | 1.2 | 0.0020 |
| 234 | 1433715_at | Cpne7 | copine VII | - | 1.2 | 0.0041 |
| 235 | 1453356_at | Cpsf7 | cleavage and polyadenylation specific factor 7 | + | 1.3 | 0.0027 |
| 236 | 1452754_at | Creld2 | cysteine-rich with EGF-like domains 2 | + | 1.3 | 0.0024 |
| 237 | 1434369_a_at | Cryab | crystallin, alpha B | + | 1.2 | 0.0074 |
| 238 | 1423845_at | Csdc2 | cold shock domain containing C2, RNA binding | - | 1.3 | 0.0057 |
| 239 | 1423593_a_at | Csf1r | colony stimulating factor 1 receptor | - | 1.2 | 0.0018 |
| 240 | 1429928_at | Ctag2 | cancer/testis antigen 2 | + | 1.2 | 0.0054 |
| 241 | 1421315_s_at | Cttn | cortactin | - | 1.2 | 0.0031 |
| 242 | 1435083_at | Ctxn1 | cortexin 1 | - | 1.3 | 0.0015 |
| 243 | 1441884_x_at | Cx3cl1 | chemokine (C-X3-C motif) ligand 1 | + | 1.2 | 0.0034 |
| 244 | 1457644_s_at | Cxcl1 | chemokine (C-X-C motif) ligand 1 | + | 1.3 | 0.0004 |
| 245 | 1417625_s_at | Cxcr7 | chemokine (C-X-C motif) receptor 7 | + | 1.2 | 0.0098 |
| 246 | 1422185_a_at | Cyb5r3 | cytochrome b5 reductase 3 | - | 1.2 | 0.0037 |
| 247 | 1426064_at | Cyp3a44 | cytochrome P450, family 3, subfamily a, polypeptide 44 | + | 1.2 | 0.0070 |
| 248 | 1422533_at | Cyp51 | cytochrome P450, family 51 | + | 1.2 | 0.0040 |
| 249 | 1419070_at | Cys1 | cystin 1 | + | 1.3 | 0.0011 |
| 250 | 1419995_at | D10Ertd641e | DNA segment, Chr 10, ERATO Doi 641, expressed | + | 1.3 | 0.0071 |
| 251 | 1443490_at | D3Ertd34e | DNA segment, Chr 3, ERATO Doi 34, expressed | + | 1.2 | 0.0075 |
| 252 | 1448538_a_at | D4Wsu53e | DNA segment, Chr 4, Wayne State University 53, expressed | + | 1.2 | 0.0089 |
| 253 | 1447526_at | D5Ertd255e | DNA segment, Chr 5, ERATO Doi 255, expressed | + | 1.2 | 0.0088 |
| 254 | 1458022_at | D830039M14Rik | RIKEN cDNA D830039M14 gene | - | 1.2 | 0.0024 |
| 255 | 1420695_at | Dach1 | dachshund 1 (Drosophila) | - | 1.2 | 0.0087 |
| 256 | 1423446_at | Dapk3 | death-associated protein kinase 3 | - | 1.3 | 0.0009 |
| 257 | 1418174_at | Dbp | D site albumin promoter binding protein | - | 1.2 | 0.0028 |
| 258 | 1451641_at | Dbr1 | debranching enzyme homolog 1 (S. cerevisiae) | + | 1.2 | 0.0049 |
| 259 | 1422521_at | Dctn1 | dynactin 1 | - | 1.2 | 0.0089 |
| 260 | 1434281_at | Dda1 | DET1 and DDB1 associated 1 | + | 1.1 | 0.0085 |
| 261 | 1419492_s_at | Defb1 | defensin beta 1 | + | 1.2 | 0.0091 |
| 262 | 1426732_at | Des | desmin | - | 1.2 | 0.0064 |
| 263 | 1459365_at | Dis3l2 | DIS3 mitotic control homolog (S. cerevisiae)-like 2 | - | 1.2 | 0.0081 |
| 264 | 1427252_at | Dmrtb1 | DMRT-like family B with proline-rich C-terminal, 1 | + | 1.2 | 0.0011 |
| 265 | 1421032_a_at | Dnajb12 | DnaJ (Hsp40) homolog, subfamily B, member 12 | - | 1.2 | 0.0022 |
| 266 | 1449373_at | Dnajc3 | DnaJ (Hsp40) homolog, subfamily C, member 3 | + | 1.2 | 0.0083 |
| 267 | 1433887_at | Dnajc3 | DnaJ (Hsp40) homolog, subfamily C, member 3 | + | 1.2 | 0.0041 |
| 268 | 1431215_at | Dnajc6 | DnaJ (Hsp40) homolog, subfamily C, member 6 | - | 1.3 | 0.0069 |
| 269 | 1427754_a_at | Dnm1 | dynamin 1 | - | 1.3 | 0.0038 |
| 270 | 1451676_at | Drap1 | Dr1 associated protein 1 (negative cofactor 2 alpha) | - | 1.2 | 0.0049 |
| 271 | 1422830_s_at | Drd4 | dopamine receptor 4 | + | 1.3 | 0.0032 |
| 272 | 1427392_at | Dscaml1 | Down syndrome cell adhesion molecule-like 1 | - | 1.2 | 0.0094 |
| 273 | 1432196_a_at | Dscaml1 | Down syndrome cell adhesion molecule-like 1 | - | 1.2 | 0.0075 |
| 274 | 1452912_at | Dscc1 | defective in sister chromatid cohesion 1 homolog (S. cerevisiae) | - | 1.2 | 0.0021 |
| 275 | 1426189_at | Dusp15 | dual specificity phosphatase-like 15 | - | 1.2 | 0.0037 |
| 276 | 1422254_a_at | Dyrk1b | dual-specificity tyrosine-(Y)-phosphorylation regulated kinase 1b | + | 1.3 | 0.0036 |
| 277 | 1438277_at | E130308A19Rik | RIKEN cDNA E130308A19 gene | + | 1.4 | 0.0010 |
| 278 | 1456585_x_at | E130309D02Rik | RIKEN cDNA E130309D02 gene | + | 1.2 | 0.0091 |
| 279 | 1433798_a_at | E330034G19Rik | RIKEN cDNA E330034G19 gene | + | 1.2 | 0.0016 |
| 280 | 1418062_at | Eef1a2 | eukaryotic translation elongation factor 1 alpha 2 | - | 1.2 | 0.0044 |
| 281 | 1428135_a_at | Eef1d | eukaryotic translation elongation factor 1 delta (guanine nucleotide exchange protein) | + | 1.2 | 0.0060 |
| 282 | 1437229_at | Efhb | EF hand domain family, member B | + | 1.3 | 0.0015 |
| 283 | 1449549_at | Efnb2 | ephrin B2 | + | 1.4 | 0.0001 |
| 284 | 1438836_at | Eftud2 | elongation factor Tu GTP binding domain containing 2 | + | 1.3 | 0.0004 |
| 285 | 1427797_s_at | EG665955 | predicted gene, EG665955 | - | 1.3 | 0.0045 |
| 286 | 1424932_at | Egfr | epidermal growth factor receptor | - | 1.1 | 0.0070 |
| 287 | 1427683_at | Egr2 | early growth response 2 | + | 1.4 | 0.0032 |
| 288 | 1436329_at | Egr3 | early growth response 3 | + | 1.2 | 0.0060 |
| 289 | 1436006_at | Eif2ak1 | eukaryotic translation initiation factor 2 alpha kinase 1 | - | 1.1 | 0.0073 |
| 290 | 1417712_at | Eif2s2 | eukaryotic translation initiation factor 2, subunit 2 (beta) | - | 1.1 | 0.0079 |
| 291 | 1455994_x_at | Elovl1 | elongation of very long chain fatty acids (FEN1/Elo2, SUR4/Elo3, yeast)-like 1 | + | 1.2 | 0.0080 |
| 292 | 1456258_at | Emx2 | empty spiracles homolog 2 (Drosophila) | + | 1.2 | 0.0026 |
| 293 | 1434580_at | Enpp4 | ectonucleotide pyrophosphatase/phosphodiesterase 4 | + | 1.2 | 0.0006 |
| 294 | 1434606_at | Erbb3 | v-erb-b2 erythroblastic leukemia viral oncogene homolog 3 (avian) | + | 1.2 | 0.0084 |
| 295 | 1440237_at | Ercc4 | excision repair cross-complementing rodent repair deficiency, complementation group 4 | - | 1.1 | 0.0061 |
| 296 | 1425265_a_at | Eri3 | exoribonuclease 3 | - | 1.3 | 0.0021 |
| 297 | 1421244_at | Esr1 | estrogen receptor 1 (alpha) | + | 1.2 | 0.0082 |
| 298 | 1451099_at | Esyt1 | extended synaptotagmin-like protein 1 | - | 1.2 | 0.0042 |
| 299 | 1418462_at | Exosc9 | exosome component 9 | - | 1.2 | 0.0071 |
| 300 | 1418290_a_at | Ezh1 | enhancer of zeste homolog 1 (Drosophila) | + | 1.3 | 0.0083 |
| 301 | 1450779_at | Fabp7 | fatty acid binding protein 7, brain | - | 1.3 | 0.0005 |
| 302 | 1449219_at | Fads3 | fatty acid desaturase 3 | - | 1.3 | 0.0094 |
| 303 | 1429764_at | Fam101b | family with sequence similarity 101, member B | + | 1.2 | 0.0099 |
| 304 | 1436140_at | Fam116b | family with sequence similarity 116, member B | - | 1.2 | 0.0040 |
| 305 | 1433639_at | Fam117a | family with sequence similarity 117, memberA | + | 1.2 | 0.0047 |
| 306 | 1432435_s_at | Fam118b | family with sequence similarity 118, member B | + | 1.2 | 0.0027 |
| 307 | 1457728_at | Fam129c | family with sequence similarity 129, member C | + | 1.2 | 0.0060 |
| 308 | 1447884_x_at | Fam134c | family with sequence similarity 134, member C | + | 1.2 | 0.0077 |
| 309 | 1457246_at | Fam160b2 | family with sequence similarity 160, member B2 | + | 1.2 | 0.0039 |
| 310 | 1443747_at | Fam48a | family with sequence similarity 48, member A | + | 1.3 | 0.0033 |
| 311 | 1431173_at | Fam53b | family with sequence similarity 53, member B | + | 1.2 | 0.0042 |
| 312 | 1420059_at | Fam54b | family with sequence similarity 54, member B | + | 1.4 | 0.0004 |
| 313 | 1452806_at | Fam57b | family with sequence similarity 57, member B | - | 1.2 | 0.0073 |
| 314 | 1423709_s_at | Farsb | phenylalanyl-tRNA synthetase, beta subunit | - | 1.2 | 0.0081 |
| 315 | 1457232_at | Fbxl21 | F-box and leucine-rich repeat protein 21 | - | 1.2 | 0.0053 |
| 316 | 1456038_at | Fbxl4 | F-box and leucine-rich repeat protein 4 | - | 1.1 | 0.0079 |
| 317 | 1426593_a_at | Fbxo22 | F-box protein 22 | + | 1.2 | 0.0024 |
| 318 | 1447671_x_at | Fbxo6 | F-box protein 6 | - | 1.2 | 0.0041 |
| 319 | 1436732_s_at | Fbxw8 | F-box and WD-40 domain protein 8 | - | 1.2 | 0.0039 |
| 320 | 1429991_at | Fezf1 | Fez family zinc finger 1 | + | 1.3 | 0.0024 |
| 321 | 1425911_a_at | Fgfr1 | fibroblast growth factor receptor 1 | - | 1.3 | 0.0055 |
| 322 | 1450728_at | Fjx1 | four jointed box 1 (Drosophila) | - | 1.2 | 0.0005 |
| 323 | 1416113_at | Fkbp8 | FK506 binding protein 8 | - | 1.3 | 0.0016 |
| 324 | 1438167_x_at | Flcn | folliculin | + | 1.1 | 0.0047 |
| 325 | 1426677_at | Flna | filamin, alpha | - | 1.2 | 0.0013 |
| 326 | 1417544_a_at | Flot2 | flotillin 2 | - | 1.2 | 0.0018 |
| 327 | 1440926_at | Flt1 | FMS-like tyrosine kinase 1 | + | 1.2 | 0.0004 |
| 328 | 1423100_at | Fos | FBJ osteosarcoma oncogene | + | 1.8 | 0.0014 |
| 329 | 1454831_at | Foxn2 | forkhead box N2 | + | 1.2 | 0.0021 |
| 330 | 1416983_s_at | Foxo1 | forkhead box O1 | - | 1.2 | 0.0070 |
| 331 | 1438169_a_at | Frmd4b | FERM domain containing 4B | + | 1.2 | 0.0006 |
| 332 | 1449121_at | Fusip1 | FUS interacting protein (serine-arginine rich) 1 | + | 1.2 | 0.0031 |
| 333 | 1419451_at | Fzr1 | fizzy/cell division cycle 20 related 1 (Drosophila) | - | 1.2 | 0.0050 |
| 334 | 1440342_at | G530011O06Rik | RIKEN cDNA G530011O06 gene | + | 1.6 | 0.0057 |
| 335 | 1428245_at | G6pc3 | glucose 6 phosphatase, catalytic, 3 | - | 1.2 | 0.0033 |
| 336 | 1442120_at | G730007D18Rik | RIKEN cDNA G730007D18 gene | + | 1.2 | 0.0004 |
| 337 | 1421263_at | Gabra3 | gamma-aminobutyric acid (GABA) A receptor, subunit alpha 3 | - | 1.3 | 0.0036 |
| 338 | 1421629_at | Gabre | gamma-aminobutyric acid (GABA) A receptor, subunit epsilon | + | 1.2 | 0.0081 |
| 339 | 1449773_s_at | Gadd45b | growth arrest and DNA-damage-inducible 45 beta | + | 1.2 | 0.0025 |
| 340 | 1446657_at | Galnt6 | UDP-N-acetyl-alpha-D-galactosamine:polypeptide N-acetylgalactosaminyltransferase 6 | + | 1.2 | 0.0018 |
| 341 | 1418240_at | Gbp2 | guanylate binding protein 2 | + | 1.2 | 0.0043 |
| 342 | 1455443_at | Gdap1l1 | ganglioside-induced differentiation-associated protein 1-like 1 | - | 1.3 | 0.0007 |
| 343 | 1433622_at | Gemin4 | gem (nuclear organelle) associated protein 4 | - | 1.2 | 0.0093 |
| 344 | 1450835_a_at | Gfra4 | glial cell line derived neurotrophic factor family receptor alpha 4 | - | 1.1 | 0.0092 |
| 345 | 1457472_at | Gigyf2 | GRB10 interacting GYF protein 2 | + | 1.2 | 0.0078 |
| 346 | 1428713_s_at | Gins2 | GINS complex subunit 2 (Psf2 homolog) | + | 1.1 | 0.0069 |
| 347 | 1445790_at | Gipr | gastric inhibitory polypeptide receptor | + | 1.2 | 0.0044 |
| 348 | 1454759_at | Git1 | G protein-coupled receptor kinase-interactor 1 | - | 1.2 | 0.0087 |
| 349 | 1422042_at | Gje1 | gap junction membrane channel protein epsilon 1 | + | 1.2 | 0.0061 |
| 350 | 1445523_at | Gje1 | gap junction membrane channel protein epsilon 1 | + | 1.2 | 0.0006 |
| 351 | 1437340_x_at | Gkn1 | gastrokine 1 | + | 1.2 | 0.0046 |
| 352 | 1449006_at | Gla | galactosidase, alpha | - | 1.2 | 0.0018 |
| 353 | 1442089_at | Gm11563 | predicted gene 11563 | + | 1.3 | 0.0057 |
| 354 | 1435188_at | Gm129 | predicted gene 129 | - | 1.3 | 0.0047 |
| 355 | 1431971_at | Gm14318 | predicted gene 14318 | + | 1.2 | 0.0082 |
| 356 | 1459006_a_at | Gm2824 | predicted gene 2824 | - | 1.2 | 0.0090 |
| 357 | 1455624_at | Gm3764 | predicted gene 3764 | - | 1.3 | 0.0002 |
| 358 | 1459503_at | Gm3764 | predicted gene 3764 | + | 1.2 | 0.0088 |
| 359 | 1443460_at | Gm4250 | predicted gene 4250 | + | 1.3 | 0.0062 |
| 360 | 1460021_at | Gm6658 | predicted gene 6658 | - | 1.2 | 0.0055 |
| 361 | 1426607_at | Gm7120 | predicted gene 7120 | + | 1.5 | 0.0026 |
| 362 | 1442246_at | Gm9054 | predicted gene 9054 | - | 1.3 | 0.0060 |
| 363 | 1453548_at | Gm9558 | predicted gene 9558 | + | 1.3 | 0.0053 |
| 364 | 1417579_x_at | Gmppa | GDP-mannose pyrophosphorylase A | - | 1.2 | 0.0066 |
| 365 | 1455458_x_at | Gmppa | GDP-mannose pyrophosphorylase A | - | 1.1 | 0.0088 |
| 366 | 1440120_at | Gnb2l1 | guanine nucleotide binding protein (G protein), beta polypeptide 2 like 1 | + | 1.2 | 0.0030 |
| 367 | 1453297_at | Golga7b | golgi autoantigen, golgin subfamily a, 7B | - | 1.2 | 0.0057 |
| 368 | 1417716_at | Got2 | glutamate oxaloacetate transaminase 2, mitochondrial | - | 1.2 | 0.0042 |
| 369 | 1450990_at | Gpc3 | glypican 3 | - | 1.4 | 0.0030 |
| 370 | 1460123_at | Gpr1 | G protein-coupled receptor 1 | - | 1.2 | 0.0055 |
| 371 | 1451060_at | Gpr146 | G protein-coupled receptor 146 | - | 1.2 | 0.0051 |
| 372 | 1434672_at | Gpr22 | G protein-coupled receptor 22 | - | 1.4 | 0.0003 |
| 373 | 1424613_at | Gprc5b | G protein-coupled receptor, family C, group 5, member B | + | 1.2 | 0.0050 |
| 374 | 1417796_at | Gps2 | G protein pathway suppressor 2 | - | 1.2 | 0.0040 |
| 375 | 1429387_at | Grap | GRB2-related adaptor protein | - | 1.2 | 0.0052 |
| 376 | 1438866_at | Grin3a | glutamate receptor ionotropic, NMDA3A | - | 1.2 | 0.0049 |
| 377 | 1425626_at | Gstm1 | glutathione S-transferase, mu 1 | + | 1.2 | 0.0086 |
| 378 | 1424835_at | Gstm4 | glutathione S-transferase, mu 4 | - | 1.2 | 0.0011 |
| 379 | 1451899_a_at | Gtf2ird1 | general transcription factor II I repeat domain-containing 1 | - | 1.2 | 0.0024 |
| 380 | 1435735_x_at | H47 | histocompatibility 47 | + | 1.2 | 0.0025 |
| 381 | 1436152_a_at | Hbxip | hepatitis B virus x interacting protein | + | 1.2 | 0.0061 |
| 382 | 1446415_at | Hebp2 | heme binding protein 2 | - | 1.2 | 0.0061 |
| 383 | 1442051_at | Hist2h3c1 | histone cluster 2, H3c1 | + | 1.2 | 0.0034 |
| 384 | 1422155_at | Hist2h3c2 | histone cluster 2, H3c2 | + | 1.3 | 0.0022 |
| 385 | 1435866_s_at | Hist3h2a | histone cluster 3, H2a | + | 1.3 | 0.0004 |
| 386 | 1420901_a_at | Hk1 | hexokinase 1 | - | 1.2 | 0.0045 |
| 387 | 1421234_at | Hnf1a | HNF1 homeobox A | + | 1.3 | 0.0036 |
| 388 | 1448408_at | Hps1 | Hermansky-Pudlak syndrome 1 homolog (human) | - | 1.3 | 0.0004 |
| 389 | 1452318_a_at | Hspa1b | heat shock protein 1B | + | 1.4 | 0.0014 |
| 390 | 1440575_at | Hspa4 | heat shock protein 4 | + | 1.2 | 0.0082 |
| 391 | 1447824_x_at | Hspa5 | heat shock protein 5 | + | 1.3 | 0.0088 |
| 392 | 1427464_s_at | Hspa5 | heat shock protein 5 | + | 1.3 | 0.0016 |
| 393 | 1416064_a_at | Hspa5 | heat shock protein 5 | + | 1.3 | 0.0008 |
| 394 | 1431274_a_at | Hspa9 | heat shock protein 9 | - | 1.2 | 0.0021 |
| 395 | 1417612_at | Ier5 | immediate early response 5 | + | 1.3 | 0.0048 |
| 396 | 1440169_x_at | Ifnar2 | interferon (alpha and beta) receptor 2 | + | 1.3 | 0.0011 |
| 397 | 1441259_s_at | Ift122 | intraflagellar transport 122 homolog (Chlamydomonas) | + | 1.2 | 0.0087 |
| 398 | 1427351_s_at | Igh-6 | immunoglobulin heavy chain 6 (heavy chain of IgM) | - | 1.3 | 0.0078 |
| 399 | 1450091_at | Ighmbp2 | immunoglobulin mu binding protein 2 | + | 1.3 | 0.0013 |
| 400 | 1420177_at | Igll1 | immunoglobulin lambda-like polypeptide 1 | - | 1.2 | 0.0012 |
| 401 | 1460675_at | Igsf8 | immunoglobulin superfamily, member 8 | - | 1.2 | 0.0036 |
| 402 | 1425625_at | Il13ra1 | interleukin 13 receptor, alpha 1 | + | 1.1 | 0.0096 |
| 403 | 1448950_at | Il1r1 | interleukin 1 receptor, type I | + | 1.2 | 0.0061 |
| 404 | 1453305_at | Iqcd | IQ motif containing D | + | 1.3 | 0.0039 |
| 405 | 1422683_at | Irak1bp1 | interleukin-1 receptor-associated kinase 1 binding protein 1 | - | 1.2 | 0.0089 |
| 406 | 1426111_x_at | Irf3 | interferon regulatory factor 3 | + | 1.2 | 0.0077 |
| 407 | 1419357_at | Isy1 | ISY1 splicing factor homolog (S. cerevisiae) | - | 1.2 | 0.0042 |
| 408 | 1436037_at | Itga4 | integrin alpha 4 | - | 1.2 | 0.0079 |
| 409 | 1436579_s_at | Itsn1 | intersectin 1 (SH3 domain protein 1A) | + | 1.2 | 0.0033 |
| 410 | 1435884_at | Itsn1 | intersectin 1 (SH3 domain protein 1A) | + | 1.2 | 0.0020 |
| 411 | 1443724_at | Jph3 | junctophilin 3 | + | 1.2 | 0.0090 |
| 412 | 1417409_at | Jun | Jun oncogene | + | 1.2 | 0.0053 |
| 413 | 1448023_at | Kalrn | kalirin, RhoGEF kinase | + | 1.2 | 0.0063 |
| 414 | 1457690_at | Kalrn | kalirin, RhoGEF kinase | + | 1.2 | 0.0058 |
| 415 | 1418544_at | Kcnip3 | Kv channel interacting protein 3, calsenilin | - | 1.4 | 0.0044 |
| 416 | 1421342_at | Kcns2 | K+ voltage-gated channel, subfamily S, 2 | + | 1.2 | 0.0094 |
| 417 | 1429741_at | Kcnv1 | potassium channel, subfamily V, member 1 | - | 1.2 | 0.0036 |
| 418 | 1417205_at | Kdelr2 | KDEL (Lys-Asp-Glu-Leu) endoplasmic reticulum protein retention receptor 2 | - | 1.2 | 0.0017 |
| 419 | 1440346_at | Kdm6b | KDM1 lysine (K)-specific demethylase 6B | + | 1.3 | 0.0055 |
| 420 | 1450108_at | Kif1a | kinesin family member 1A | - | 1.2 | 0.0045 |
| 421 | 1426493_a_at | Kifc2 | kinesin family member C2 | - | 1.3 | 0.0012 |
| 422 | 1421312_a_at | Kifc2 | kinesin family member C2 | - | 1.2 | 0.0058 |
| 423 | 1448117_at | Kitl | kit ligand | - | 1.3 | 0.0054 |
| 424 | 1448164_at | Klhdc3 | kelch domain containing 3 | - | 1.3 | 0.0001 |
| 425 | 1424887_at | Klhdc4 | kelch domain containing 4 | - | 1.2 | 0.0059 |
| 426 | 1425664_at | Klhl20 | kelch-like 20 (Drosophila) | - | 1.2 | 0.0095 |
| 427 | 1435575_at | Kntc1 | kinetochore associated 1 | - | 1.2 | 0.0043 |
| 428 | 1435472_at | Kremen1 | kringle containing transmembrane protein 1 | + | 1.1 | 0.0087 |
| 429 | 1430669_at | Krtap4-7 | keratin associated protein 4-7 | + | 1.2 | 0.0057 |
| 430 | 1425141_at | Lactb2 | lactamase, beta 2 | - | 1.2 | 0.0091 |
| 431 | 1436915_x_at | Laptm4b | lysosomal-associated protein transmembrane 4B | + | 1.2 | 0.0022 |
| 432 | 1438633_x_at | Lasp1 | LIM and SH3 protein 1 | + | 1.2 | 0.0063 |
| 433 | 1439479_at | Lct | lactase | + | 1.2 | 0.0017 |
| 434 | 1450383_at | Ldlr | low density lipoprotein receptor | - | 1.3 | 0.0002 |
| 435 | 1436900_x_at | Leprot | leptin receptor overlapping transcript | + | 1.2 | 0.0044 |
| 436 | 1435499_at | Letm2 | leucine zipper-EF-hand containing transmembrane protein 2 | - | 1.1 | 0.0096 |
| 437 | 1422662_at | Lgals8 | lectin, galactose binding, soluble 8 | - | 1.1 | 0.0083 |
| 438 | 1456838_at | Lingo3 | leucine rich repeat and Ig domain containing 3 | - | 1.2 | 0.0090 |
| 439 | 1431492_at | Lipn | lipase, family member N | + | 1.3 | 0.0088 |
| 440 | 1428129_at | Lman1 | lectin, mannose-binding, 1 | + | 1.2 | 0.0020 |
| 441 | 1444491_at | LOC100042150 | similar to NTAK alpha2 | + | 1.2 | 0.0023 |
| 442 | 1454666_at | LOC100046855 | similar to BKLF | + | 1.1 | 0.0088 |
| 443 | 1455557_at | LOC553095 | hypothetical LOC553095 | + | 1.2 | 0.0084 |
| 444 | 1429863_at | Lonrf3 | LON peptidase N-terminal domain and ring finger 3 | + | 1.2 | 0.0094 |
| 445 | 1440207_at | Lpcat4 | lysophosphatidylcholine acyltransferase 4 | - | 1.2 | 0.0078 |
| 446 | 1444669_at | Lrfn1 | leucine rich repeat and fibronectin type III domain containing 1 | - | 1.2 | 0.0089 |
| 447 | 1436083_at | Lrp3 | low density lipoprotein receptor-related protein 3 | - | 1.2 | 0.0019 |
| 448 | 1426288_at | Lrp4 | low density lipoprotein receptor-related protein 4 | + | 1.2 | 0.0013 |
| 449 | 1455883_a_at | Lrrtm1 | leucine rich repeat transmembrane neuronal 1 | + | 1.2 | 0.0084 |
| 450 | 1428437_at | Lsm14a | LSM14 homolog A (SCD6, S. cerevisiae) | + | 1.1 | 0.0097 |
| 451 | 1453304_s_at | Ly6e | lymphocyte antigen 6 complex, locus E | - | 1.3 | 0.0009 |
| 452 | 1447945_at | Maf | avian musculoaponeurotic fibrosarcoma (v-maf) AS42 oncogene homolog | - | 1.2 | 0.0056 |
| 453 | 1428112_at | Manf | mesencephalic astrocyte-derived neurotrophic factor | + | 1.2 | 0.0034 |
| 454 | 1453712_a_at | Map2k5 | mitogen-activated protein kinase kinase 5 | - | 1.2 | 0.0098 |
| 455 | 1421416_at | Map2k7 | mitogen-activated protein kinase kinase 7 | + | 1.2 | 0.0038 |
| 456 | 1443540_at | Map3k1 | mitogen-activated protein kinase kinase kinase 1 | + | 1.2 | 0.0092 |
| 457 | 1447667_x_at | Map3k4 | mitogen-activated protein kinase kinase kinase 4 | + | 1.1 | 0.0098 |
| 458 | 1455441_at | Map3k7 | mitogen-activated protein kinase kinase kinase 7 | - | 1.2 | 0.0048 |
| 459 | 1427079_at | Mapre3 | microtubule-associated protein, RP/EB family, member 3 | - | 1.4 | 0.0001 |
| 460 | 1455346_at | Masp1 | mannan-binding lectin serine peptidase 1 | - | 1.2 | 0.0062 |
| 461 | 1455978_a_at | Matn2 | matrilin 2 | + | 1.2 | 0.0045 |
| 462 | 1449490_at | Mbd4 | methyl-CpG binding domain protein 4 | + | 1.2 | 0.0028 |
| 463 | 1418787_at | Mbl2 | mannose-binding lectin (protein C) 2 | - | 1.1 | 0.0068 |
| 464 | 1456381_x_at | Mcl1 | myeloid cell leukemia sequence 1 | + | 1.2 | 0.0089 |
| 465 | 1416006_at | Mdk | midkine | - | 1.3 | 0.0036 |
| 466 | 1425585_at | Med12 | mediator of RNA polymerase II transcription, subunit 12 homolog (yeast) | - | 1.2 | 0.0069 |
| 467 | 1456785_at | Med14 | mediator complex subunit 14 | - | 1.2 | 0.0057 |
| 468 | 1416826_a_at | Med20 | mediator complex subunit 20 | + | 1.3 | 0.0025 |
| 469 | 1426758_s_at | Meg3 | maternally expressed 3 | - | 1.2 | 0.0083 |
| 470 | 1423984_a_at | Meis3 | Meis homeobox 3 | - | 1.2 | 0.0071 |
| 471 | 1426837_at | Metap1 | methionyl aminopeptidase 1 | - | 1.2 | 0.0069 |
| 472 | 1453983_a_at | Mett10d | methyltransferase 10 domain containing | + | 1.2 | 0.0067 |
| 473 | 1447683_x_at | Mettl1 | methyltransferase like 1 | - | 1.2 | 0.0050 |
| 474 | 1416358_at | Mfsd10 | major facilitator superfamily domain containing 10 | - | 1.2 | 0.0061 |
| 475 | 1444199_at | Mfsd4 | major facilitator superfamily domain containing 4 | + | 1.2 | 0.0070 |
| 476 | 1417120_at | Miip | migration and invasion inhibitory protein | - | 1.2 | 0.0076 |
| 477 | 1454014_a_at | Mkks | McKusick-Kaufman syndrome protein | + | 1.2 | 0.0062 |
| 478 | 1427283_at | Mll1 | myeloid/lymphoid or mixed-lineage leukemia 1 | - | 1.2 | 0.0072 |
| 479 | 1459611_at | Mpp3 | membrane protein, palmitoylated 3 (MAGUK p55 subfamily member 3) | - | 1.2 | 0.0077 |
| 480 | 1418356_at | Mpst | mercaptopyruvate sulfurtransferase | + | 1.2 | 0.0058 |
| 481 | 1428167_a_at | Mpzl1 | myelin protein zero-like 1 | + | 1.3 | 0.0000 |
| 482 | 1417146_at | Mri1 | methylthioribose-1-phosphate isomerase homolog (S. cerevisiae) | - | 1.2 | 0.0035 |
| 483 | 1460354_a_at | Mrpl13 | mitochondrial ribosomal protein L13 | + | 1.1 | 0.0045 |
| 484 | 1444306_at | Msi1 | Musashi homolog 1(Drosophila) | + | 1.2 | 0.0019 |
| 485 | 1460566_at | Mtap1a | microtubule-associated protein 1 A | - | 1.2 | 0.0030 |
| 486 | 1429894_a_at | Mtap7 | microtubule-associated protein 7 | + | 1.2 | 0.0080 |
| 487 | 1449199_at | Muc1 | mucin 1, transmembrane | - | 1.2 | 0.0035 |
| 488 | 1430539_at | Mxra7 | matrix-remodelling associated 7 | + | 1.2 | 0.0080 |
| 489 | 1427769_x_at | Myl3 | myosin, light polypeptide 3 | + | 1.3 | 0.0077 |
| 490 | 1460159_at | Mysm1 | myb-like, SWIRM and MPN domains 1 | - | 1.3 | 0.0049 |
| 491 | 1446957_s_at | N4bp1 | NEDD4 binding protein 1 | + | 1.2 | 0.0063 |
| 492 | 1435269_at | N6amt2 | N-6 adenine-specific DNA methyltransferase 2 (putative) | - | 1.2 | 0.0021 |
| 493 | 1455869_at | NA | NA | - | 1.8 | 0.0009 |
| 494 | 1435353_a_at | NA | NA | - | 1.6 | 0.0057 |
| 495 | 1431213_a_at | NA | NA | - | 1.5 | 0.0037 |
| 496 | 1423916_s_at | NA | NA | - | 1.4 | 0.0008 |
| 497 | 1434270_at | NA | NA | - | 1.4 | 0.0003 |
| 498 | 1440030_at | NA | NA | - | 1.4 | 0.0074 |
| 499 | 1450455_s_at | NA | NA | - | 1.3 | 0.0055 |
| 500 | 1456160_at | NA | NA | - | 1.3 | 0.0077 |
| 501 | 1422319_at | NA | NA | - | 1.3 | 0.0002 |
| 502 | 1437183_at | NA | NA | - | 1.3 | 0.0067 |
| 503 | 1459062_x_at | NA | NA | - | 1.3 | 0.0033 |
| 504 | 1447559_at | NA | NA | - | 1.3 | 0.0007 |
| 505 | 1420084_at | NA | NA | - | 1.3 | 0.0011 |
| 506 | 1429546_at | NA | NA | - | 1.3 | 0.0010 |
| 507 | 1440055_at | NA | NA | - | 1.3 | 0.0074 |
| 508 | 1417524_at | NA | NA | - | 1.3 | 0.0027 |
| 509 | 1444495_at | NA | NA | - | 1.3 | 0.0004 |
| 510 | 1446571_at | NA | NA | - | 1.3 | 0.0021 |
| 511 | 1440380_at | NA | NA | - | 1.3 | 0.0094 |
| 512 | 1440099_at | NA | NA | - | 1.2 | 0.0069 |
| 513 | 1442386_at | NA | NA | - | 1.2 | 0.0054 |
| 514 | 1441472_at | NA | NA | - | 1.2 | 0.0007 |
| 515 | 1457920_at | NA | NA | - | 1.2 | 0.0019 |
| 516 | 1418768_at | NA | NA | - | 1.2 | 0.0026 |
| 517 | 1459590_at | NA | NA | - | 1.2 | 0.0017 |
| 518 | 1446182_at | NA | NA | - | 1.2 | 0.0071 |
| 519 | 1448796_s_at | NA | NA | - | 1.2 | 0.0092 |
| 520 | 1455503_at | NA | NA | - | 1.2 | 0.0097 |
| 521 | 1415896_x_at | NA | NA | - | 1.2 | 0.0021 |
| 522 | 1445110_at | NA | NA | - | 1.2 | 0.0055 |
| 523 | 1415895_at | NA | NA | - | 1.2 | 0.0022 |
| 524 | 1445564_at | NA | NA | - | 1.2 | 0.0061 |
| 525 | 1443707_at | NA | NA | - | 1.2 | 0.0029 |
| 526 | 1421181_at | NA | NA | - | 1.2 | 0.0013 |
| 527 | 1444510_at | NA | NA | - | 1.2 | 0.0020 |
| 528 | 1447223_at | NA | NA | - | 1.2 | 0.0027 |
| 529 | 1443544_at | NA | NA | - | 1.2 | 0.0030 |
| 530 | 1421063_s_at | NA | NA | - | 1.2 | 0.0015 |
| 531 | 1426040_a_at | NA | NA | - | 1.2 | 0.0028 |
| 532 | 1452100_at | NA | NA | - | 1.2 | 0.0032 |
| 533 | 1440755_at | NA | NA | - | 1.2 | 0.0065 |
| 534 | 1443038_at | NA | NA | - | 1.2 | 0.0062 |
| 535 | 1436599_at | NA | NA | - | 1.2 | 0.0037 |
| 536 | 1445717_at | NA | NA | - | 1.2 | 0.0099 |
| 537 | 1438780_at | NA | NA | - | 1.2 | 0.0054 |
| 538 | 1419794_at | NA | NA | - | 1.2 | 0.0087 |
| 539 | 1458324_x_at | NA | NA | - | 1.2 | 0.0044 |
| 540 | 1427968_at | NA | NA | - | 1.2 | 0.0096 |
| 541 | 1445485_at | NA | NA | - | 1.2 | 0.0085 |
| 542 | 1445603_at | NA | NA | - | 1.2 | 0.0066 |
| 543 | 1441164_at | NA | NA | - | 1.2 | 0.0048 |
| 544 | 1446372_at | NA | NA | - | 1.2 | 0.0093 |
| 545 | 1435716_x_at | NA | NA | - | 1.2 | 0.0051 |
| 546 | 1458341_x_at | NA | NA | - | 1.2 | 0.0065 |
| 547 | 1460072_at | NA | NA | - | 1.1 | 0.0082 |
| 548 | 1442087_at | NA | NA | + | 1.1 | 0.0081 |
| 549 | 1459367_at | NA | NA | + | 1.1 | 0.0067 |
| 550 | 1454639_x_at | NA | NA | + | 1.1 | 0.0051 |
| 551 | 1434848_at | NA | NA | + | 1.1 | 0.0052 |
| 552 | 1445569_at | NA | NA | + | 1.1 | 0.0076 |
| 553 | 1438553_x_at | NA | NA | + | 1.1 | 0.0058 |
| 554 | 1442681_at | NA | NA | + | 1.2 | 0.0093 |
| 555 | 1443050_at | NA | NA | + | 1.2 | 0.0065 |
| 556 | 1444971_at | NA | NA | + | 1.2 | 0.0078 |
| 557 | 1435129_at | NA | NA | + | 1.2 | 0.0063 |
| 558 | 1459285_at | NA | NA | + | 1.2 | 0.0055 |
| 559 | 1445590_at | NA | NA | + | 1.2 | 0.0099 |
| 560 | 1447000_at | NA | NA | + | 1.2 | 0.0091 |
| 561 | 1438596_at | NA | NA | + | 1.2 | 0.0064 |
| 562 | 1439683_at | NA | NA | + | 1.2 | 0.0070 |
| 563 | 1458406_at | NA | NA | + | 1.2 | 0.0040 |
| 564 | 1444473_at | NA | NA | + | 1.2 | 0.0045 |
| 565 | 1445729_at | NA | NA | + | 1.2 | 0.0045 |
| 566 | 1457637_at | NA | NA | + | 1.2 | 0.0083 |
| 567 | 1457247_at | NA | NA | + | 1.2 | 0.0036 |
| 568 | 1424972_at | NA | NA | + | 1.2 | 0.0083 |
| 569 | 1453682_at | NA | NA | + | 1.2 | 0.0073 |
| 570 | 1437026_at | NA | NA | + | 1.2 | 0.0069 |
| 571 | 1449760_at | NA | NA | + | 1.2 | 0.0090 |
| 572 | 1438908_at | NA | NA | + | 1.2 | 0.0029 |
| 573 | 1443354_at | NA | NA | + | 1.2 | 0.0046 |
| 574 | 1439600_at | NA | NA | + | 1.2 | 0.0065 |
| 575 | 1446784_at | NA | NA | + | 1.2 | 0.0045 |
| 576 | 1445529_at | NA | NA | + | 1.2 | 0.0097 |
| 577 | 1423585_at | NA | NA | + | 1.2 | 0.0070 |
| 578 | 1420128_s_at | NA | NA | + | 1.2 | 0.0079 |
| 579 | 1425872_at | NA | NA | + | 1.2 | 0.0075 |
| 580 | 1447958_at | NA | NA | + | 1.2 | 0.0047 |
| 581 | 1434589_x_at | NA | NA | + | 1.2 | 0.0038 |
| 582 | 1442929_at | NA | NA | + | 1.2 | 0.0029 |
| 583 | 1434150_a_at | NA | NA | + | 1.2 | 0.0061 |
| 584 | 1442527_at | NA | NA | + | 1.2 | 0.0062 |
| 585 | 1435311_s_at | NA | NA | + | 1.2 | 0.0011 |
| 586 | 1459071_at | NA | NA | + | 1.2 | 0.0019 |
| 587 | 1447176_at | NA | NA | + | 1.2 | 0.0047 |
| 588 | 1444057_at | NA | NA | + | 1.2 | 0.0056 |
| 589 | 1457132_at | NA | NA | + | 1.2 | 0.0037 |
| 590 | 1458692_at | NA | NA | + | 1.2 | 0.0012 |
| 591 | 1458598_at | NA | NA | + | 1.2 | 0.0020 |
| 592 | 1458494_at | NA | NA | + | 1.2 | 0.0057 |
| 593 | 1436687_x_at | NA | NA | + | 1.2 | 0.0005 |
| 594 | 1459733_at | NA | NA | + | 1.2 | 0.0086 |
| 595 | 1445735_at | NA | NA | + | 1.2 | 0.0031 |
| 596 | 1459419_at | NA | NA | + | 1.2 | 0.0060 |
| 597 | 1422096_at | NA | NA | + | 1.2 | 0.0030 |
| 598 | 1442435_at | NA | NA | + | 1.2 | 0.0069 |
| 599 | 1446335_at | NA | NA | + | 1.2 | 0.0074 |
| 600 | 1438931_s_at | NA | NA | + | 1.2 | 0.0009 |
| 601 | 1439053_at | NA | NA | + | 1.2 | 0.0082 |
| 602 | 1439265_at | NA | NA | + | 1.2 | 0.0083 |
| 603 | 1438622_x_at | NA | NA | + | 1.2 | 0.0005 |
| 604 | 1445755_at | NA | NA | + | 1.2 | 0.0086 |
| 605 | 1447560_at | NA | NA | + | 1.2 | 0.0060 |
| 606 | 1456909_at | NA | NA | + | 1.2 | 0.0006 |
| 607 | 1458805_at | NA | NA | + | 1.3 | 0.0011 |
| 608 | 1432828_at | NA | NA | + | 1.3 | 0.0092 |
| 609 | 1437767_s_at | NA | NA | + | 1.3 | 0.0009 |
| 610 | 1437455_a_at | NA | NA | + | 1.3 | 0.0021 |
| 611 | 1447479_at | NA | NA | + | 1.3 | 0.0028 |
| 612 | 1447511_at | NA | NA | + | 1.3 | 0.0055 |
| 613 | 1447454_at | NA | NA | + | 1.3 | 0.0077 |
| 614 | 1434029_at | NA | NA | + | 1.3 | 0.0095 |
| 615 | 1456687_at | NA | NA | + | 1.3 | 0.0024 |
| 616 | 1445262_at | NA | NA | + | 1.3 | 0.0066 |
| 617 | 1457015_at | NA | NA | + | 1.3 | 0.0001 |
| 618 | 1443770_x_at | NA | NA | + | 1.3 | 0.0024 |
| 619 | 1422416_s_at | NA | NA | + | 1.3 | 0.0008 |
| 620 | 1419946_s_at | NA | NA | + | 1.3 | 0.0020 |
| 621 | 1438009_at | NA | NA | + | 1.3 | 0.0002 |
| 622 | 1420287_at | NA | NA | + | 1.3 | 0.0007 |
| 623 | 1441530_at | NA | NA | + | 1.3 | 0.0057 |
| 624 | 1449790_at | NA | NA | + | 1.3 | 0.0026 |
| 625 | 1458629_at | NA | NA | + | 1.4 | 0.0017 |
| 626 | 1445977_at | NA | NA | + | 1.4 | 0.0002 |
| 627 | 1444913_at | NA | NA | + | 1.4 | 0.0021 |
| 628 | 1420094_at | NA | NA | + | 1.4 | 0.0001 |
| 629 | 1419937_at | NA | NA | + | 1.4 | 0.0001 |
| 630 | 1454284_at | NA | NA | + | 1.4 | 0.0002 |
| 631 | 1459066_at | NA | NA | + | 1.5 | 0.0026 |
| 632 | 1437729_at | NA | NA | + | 1.5 | 0.0018 |
| 633 | 1449727_x_at | NA | NA | + | 1.5 | 0.0015 |
| 634 | 1419915_at | NA | NA | + | 1.6 | 0.0003 |
| 635 | 1449788_at | NA | NA | + | 1.6 | 0.0002 |
| 636 | 1454378_at | NA | NA | + | 1.8 | 0.0035 |
| 637 | 1420968_at | Nacc1 | nucleus accumbens associated 1, BEN and BTB (POZ) domain containing | + | 1.2 | 0.0020 |
| 638 | 1418570_at | Ncstn | nicastrin | - | 1.2 | 0.0071 |
| 639 | 1453565_at | Ndufab1 | NADH dehydrogenase (ubiquinone) 1, alpha/beta subcomplex, 1 | + | 1.2 | 0.0095 |
| 640 | 1428160_at | Ndufab1 | NADH dehydrogenase (ubiquinone) 1, alpha/beta subcomplex, 1 | + | 1.3 | 0.0040 |
| 641 | 1431670_at | Ndufc2 | NADH dehydrogenase (ubiquinone) 1, subcomplex unknown, 2 | + | 1.3 | 0.0048 |
| 642 | 1418881_at | Necab2 | N-terminal EF-hand calcium binding protein 2 | - | 1.2 | 0.0050 |
| 643 | 1425714_a_at | Nfam1 | Nfat activating molecule with ITAM motif 1 | + | 1.3 | 0.0007 |
| 644 | 1455891_at | Nhedc1 | Na+/H+ exchanger domain containing 1 | + | 1.3 | 0.0033 |
| 645 | 1429145_at | Nhlrc2 | NHL repeat containing 2 | + | 1.2 | 0.0035 |
| 646 | 1456467_s_at | Nlk | nemo like kinase | + | 1.1 | 0.0067 |
| 647 | 1424899_at | Nmnat3 | nicotinamide nucleotide adenylyltransferase 3 | + | 1.2 | 0.0073 |
| 648 | 1423506_a_at | Nnat | neuronatin | - | 1.4 | 0.0013 |
| 649 | 1416105_at | Nnt | nicotinamide nucleotide transhydrogenase | - | 1.2 | 0.0088 |
| 650 | 1420487_at | Nol7 | nucleolar protein 7 | + | 1.2 | 0.0076 |
| 651 | 1453114_at | Nol9 | nucleolar protein 9 | + | 1.2 | 0.0087 |
| 652 | 1425151_a_at | Noxo1 | NADPH oxidase organizer 1 | - | 1.2 | 0.0037 |
| 653 | 1432416_a_at | Npm1 | nucleophosmin 1 | - | 1.2 | 0.0034 |
| 654 | 1452107_s_at | Npnt | nephronectin | + | 1.2 | 0.0027 |
| 655 | 1427191_at | Npr2 | natriuretic peptide receptor 2 | - | 1.3 | 0.0001 |
| 656 | 1454167_at | Nr2c2 | nuclear receptor subfamily 2, group C, member 2 | + | 1.2 | 0.0022 |
| 657 | 1457635_s_at | Nr3c1 | nuclear receptor subfamily 3, group C, member 1 | + | 1.2 | 0.0079 |
| 658 | 1434640_at | Nrsn2 | neurensin 2 | - | 1.3 | 0.0039 |
| 659 | 1455499_at | Nrxn2 | neurexin II | - | 1.4 | 0.0008 |
| 660 | 1455387_at | Nufip2 | nuclear fragile X mental retardation protein interacting protein 2 | + | 1.4 | 0.0004 |
| 661 | 1435216_a_at | Odf2 | outer dense fiber of sperm tails 2 | - | 1.2 | 0.0087 |
| 662 | 1455796_x_at | Olfm1 | olfactomedin 1 | + | 1.1 | 0.0074 |
| 663 | 1426512_at | Olfm3 | olfactomedin 3 | - | 1.3 | 0.0005 |
| 664 | 1455743_at | Olfml2a | olfactomedin-like 2A | + | 1.3 | 0.0054 |
| 665 | 1418212_at | Omg | oligodendrocyte myelin glycoprotein | - | 1.1 | 0.0086 |
| 666 | 1458350_at | Opa3 | optic atrophy 3 (human) | + | 1.2 | 0.0081 |
| 667 | 1459402_at | Oprm1 | opioid receptor, mu 1 | + | 1.2 | 0.0032 |
| 668 | 1424235_at | Ormdl2 | ORM1-like 2 (S. cerevisiae) | + | 1.2 | 0.0055 |
| 669 | 1449627_at | Osbpl6 | oxysterol binding protein-like 6 | + | 1.3 | 0.0008 |
| 670 | 1425540_at | Otc | ornithine transcarbamylase | - | 1.3 | 0.0037 |
| 671 | 1448817_at | Otub1 | OTU domain, ubiquitin aldehyde binding 1 | - | 1.2 | 0.0010 |
| 672 | 1417575_at | Otub2 | OTU domain, ubiquitin aldehyde binding 2 | + | 1.2 | 0.0015 |
| 673 | 1452094_at | P4ha1 | procollagen-proline, 2-oxoglutarate 4-dioxygenase (proline 4-hydroxylase), alpha 1 polypeptide | + | 1.2 | 0.0032 |
| 674 | 1460265_at | Pa2g4 | proliferation-associated 2G4 | + | 1.2 | 0.0018 |
| 675 | 1449381_a_at | Pacsin1 | protein kinase C and casein kinase substrate in neurons 1 | - | 1.3 | 0.0009 |
| 676 | 1455216_at | Paqr6 | progestin and adipoQ receptor family member VI | - | 1.2 | 0.0085 |
| 677 | 1449975_a_at | Park2 | Parkinson disease (autosomal recessive, juvenile) 2, parkin | - | 1.2 | 0.0008 |
| 678 | 1434363_x_at | Paxip1 | PAX interacting (with transcription-activation domain) protein 1 | + | 1.2 | 0.0053 |
| 679 | 1427956_at | Pcgf1 | polycomb group ring finger 1 | - | 1.2 | 0.0004 |
| 680 | 1432108_at | Pcgf6 | polycomb group ring finger 6 | + | 1.2 | 0.0050 |
| 681 | 1454120_a_at | Pcgf6 | polycomb group ring finger 6 | + | 1.2 | 0.0010 |
| 682 | 1425824_a_at | Pcsk4 | proprotein convertase subtilisin/kexin type 4 | - | 1.2 | 0.0033 |
| 683 | 1415956_a_at | Pctk1 | PCTAIRE-motif protein kinase 1 | - | 1.3 | 0.0040 |
| 684 | 1449151_at | Pctk3 | PCTAIRE-motif protein kinase 3 | - | 1.3 | 0.0010 |
| 685 | 1460263_at | Pdcd6ip | programmed cell death 6 interacting protein | + | 1.3 | 0.0007 |
| 686 | 1425100_a_at | Pde6g | phosphodiesterase 6G, cGMP-specific, rod, gamma | + | 1.3 | 0.0029 |
| 687 | 1428308_at | Pdrg1 | p53 and DNA damage regulated 1 | + | 1.2 | 0.0076 |
| 688 | 1438358_x_at | Pfdn5 | prefoldin 5 | + | 1.1 | 0.0052 |
| 689 | 1439148_a_at | Pfkl | phosphofructokinase, liver, B-type | - | 1.2 | 0.0073 |
| 690 | 1449018_at | Pfn1 | profilin 1 | - | 1.3 | 0.0017 |
| 691 | 1437239_x_at | Phc2 | polyhomeotic-like 2 (Drosophila) | + | 1.2 | 0.0010 |
| 692 | 1418620_at | Phox2a | paired-like homeobox 2a | + | 1.5 | 0.0000 |
| 693 | 1427903_at | Phpt1 | phosphohistidine phosphatase 1 | - | 1.2 | 0.0068 |
| 694 | 1421284_at | Pign | phosphatidylinositol glycan anchor biosynthesis, class N | + | 1.2 | 0.0065 |
| 695 | 1437594_x_at | Pigt | phosphatidylinositol glycan anchor biosynthesis, class T | + | 1.4 | 0.0017 |
| 696 | 1455284_x_at | Pigx | phosphatidylinositol glycan anchor biosynthesis, class X | + | 1.2 | 0.0027 |
| 697 | 1425514_at | Pik3r1 | phosphatidylinositol 3-kinase, regulatory subunit, polypeptide 1 (p85 alpha) | + | 1.2 | 0.0049 |
| 698 | 1424954_a_at | Pip5k1c | phosphatidylinositol-4-phosphate 5-kinase, type 1 gamma | - | 1.2 | 0.0085 |
| 699 | 1420307_a_at | Pitpnb | phosphatidylinositol transfer protein, beta | - | 1.3 | 0.0001 |
| 700 | 1452940_x_at | Pitpnc1 | phosphatidylinositol transfer protein, cytoplasmic 1 | + | 1.2 | 0.0049 |
| 701 | 1454838_s_at | Pkdcc | protein kinase domain containing, cytoplasmic | + | 1.2 | 0.0034 |
| 702 | 1434820_s_at | Pkig | protein kinase inhibitor, gamma | + | 1.2 | 0.0024 |
| 703 | 1417308_at | Pkm2 | pyruvate kinase, muscle | - | 1.2 | 0.0077 |
| 704 | 1426208_x_at | Plagl1 | pleiomorphic adenoma gene-like 1 | - | 1.3 | 0.0080 |
| 705 | 1417963_at | Pltp | phospholipid transfer protein | - | 1.3 | 0.0092 |
| 706 | 1448757_at | Pml | promyelocytic leukemia | + | 1.2 | 0.0053 |
| 707 | 1416378_at | Pnkp | polynucleotide kinase 3'- phosphatase | - | 1.2 | 0.0024 |
| 708 | 1433431_at | Pnlip | pancreatic lipase | + | 1.3 | 0.0087 |
| 709 | 1455622_at | Podxl2 | podocalyxin-like 2 | - | 1.2 | 0.0056 |
| 710 | 1420910_at | Ppap2c | phosphatidic acid phosphatase type 2C | + | 1.4 | 0.0029 |
| 711 | 1428344_at | Ppapdc2 | phosphatidic acid phosphatase type 2 domain containing 2 | - | 1.2 | 0.0016 |
| 712 | 1451943_a_at | Ppm1a | protein phosphatase 1A, magnesium dependent, alpha isoform | - | 1.2 | 0.0058 |
| 713 | 1416618_at | Ppox | protoporphyrinogen oxidase | - | 1.2 | 0.0061 |
| 714 | 1431328_at | Ppp1cb | protein phosphatase 1, catalytic subunit, beta isoform | + | 1.2 | 0.0024 |
| 715 | 1448565_at | Ppp1r11 | protein phosphatase 1, regulatory (inhibitor) subunit 11 | - | 1.3 | 0.0016 |
| 716 | 1424554_at | Ppp1r8 | protein phosphatase 1, regulatory (inhibitor) subunit 8 | - | 1.3 | 0.0050 |
| 717 | 1415819_a_at | Ppp2r1a | protein phosphatase 2 (formerly 2A), regulatory subunit A (PR 65), alpha isoform | - | 1.4 | 0.0009 |
| 718 | 1420034_at | Ppp2r2d | protein phosphatase 2, regulatory subunit B, delta isoform | + | 1.4 | 0.0099 |
| 719 | 1455198_a_at | Ppp2r3d | protein phosphatase 2 (formerly 2A), regulatory subunit B'', delta | - | 1.2 | 0.0023 |
| 720 | 1440189_at | Ppp2r5a | protein phosphatase 2, regulatory subunit B (B56), alpha isoform | + | 1.2 | 0.0051 |
| 721 | 1434205_at | Ppp2r5c | protein phosphatase 2, regulatory subunit B (B56), gamma isoform | + | 1.2 | 0.0075 |
| 722 | 1439942_at | Prep | prolyl endopeptidase | - | 1.2 | 0.0002 |
| 723 | 1424119_at | Prkab1 | protein kinase, AMP-activated, beta 1 non-catalytic subunit | - | 1.1 | 0.0081 |
| 724 | 1433533_x_at | Prkag1 | protein kinase, AMP-activated, gamma 1 non-catalytic subunit | + | 1.1 | 0.0076 |
| 725 | 1421446_at | Prkcc | protein kinase C, gamma | - | 1.3 | 0.0014 |
| 726 | 1422657_at | Prl5a1 | prolactin family 5, subfamily a, member 1 | + | 1.2 | 0.0023 |
| 727 | 1454789_x_at | Prpf6 | PRP6 pre-mRNA splicing factor 6 homolog (yeast) | + | 1.2 | 0.0091 |
| 728 | 1432129_a_at | Prrx1 | paired related homeobox 1 | + | 1.2 | 0.0027 |
| 729 | 1433573_x_at | Prss2 | protease, serine, 2 | + | 1.2 | 0.0061 |
| 730 | 1417682_a_at | Prss2 | protease, serine, 2 | + | 1.2 | 0.0037 |
| 731 | 1454607_s_at | Psat1 | phosphoserine aminotransferase 1 | + | 1.1 | 0.0078 |
| 732 | 1420052_x_at | Psmb1 | proteasome (prosome, macropain) subunit, beta type 1 | + | 1.2 | 0.0021 |
| 733 | 1420053_at | Psmb1 | proteasome (prosome, macropain) subunit, beta type 1 | + | 1.2 | 0.0036 |
| 734 | 1416240_at | Psmb7 | proteasome (prosome, macropain) subunit, beta type 7 | - | 1.3 | 0.0063 |
| 735 | 1453854_at | Ptar1 | protein prenyltransferase alpha subunit repeat containing 1 | + | 1.2 | 0.0056 |
| 736 | 1417263_at | Ptgs2 | prostaglandin-endoperoxide synthase 2 | - | 1.2 | 0.0028 |
| 737 | 1428707_at | Ptms | parathymosin | - | 1.2 | 0.0056 |
| 738 | 1428708_x_at | Ptms | parathymosin | - | 1.2 | 0.0040 |
| 739 | 1438058_s_at | Ptov1 | prostate tumor over expressed gene 1 | - | 1.2 | 0.0098 |
| 740 | 1418181_at | Ptp4a3 | protein tyrosine phosphatase 4a3 | - | 1.2 | 0.0019 |
| 741 | 1454726_s_at | Ptpdc1 | protein tyrosine phosphatase domain containing 1 | - | 1.2 | 0.0082 |
| 742 | 1416588_at | Ptprn | protein tyrosine phosphatase, receptor type, N | - | 1.2 | 0.0010 |
| 743 | 1420131_s_at | Pttg1ip | pituitary tumor-transforming 1 interacting protein | + | 1.2 | 0.0031 |
| 744 | 1420132_s_at | Pttg1ip | pituitary tumor-transforming 1 interacting protein | + | 1.2 | 0.0038 |
| 745 | 1450819_at | Pvrl1 | poliovirus receptor-related 1 | + | 1.2 | 0.0028 |
| 746 | 1451253_at | Pxk | PX domain containing serine/threonine kinase | - | 1.2 | 0.0027 |
| 747 | 1423448_at | Rab11b | RAB11B, member RAS oncogene family | - | 1.2 | 0.0073 |
| 748 | 1417214_at | Rab27b | RAB27b, member RAS oncogene family | - | 1.2 | 0.0030 |
| 749 | 1455857_a_at | Rab2b | RAB2B, member RAS oncogene family | - | 1.2 | 0.0005 |
| 750 | 1422589_at | Rab3a | RAB3A, member RAS oncogene family | - | 1.3 | 0.0018 |
| 751 | 1425266_a_at | Rap1gds1 | RAP1, GTP-GDP dissociation stimulator 1 | - | 1.2 | 0.0072 |
| 752 | 1427975_at | Rasl10a | RAS-like, family 10, member A | + | 1.3 | 0.0046 |
| 753 | 1452091_a_at | Rbm28 | RNA binding motif protein 28 | + | 1.1 | 0.0086 |
| 754 | 1429169_at | Rbm3 | RNA binding motif protein 3 | + | 1.3 | 0.0025 |
| 755 | 1418245_a_at | Rbm9 | RNA binding motif protein 9 | + | 1.2 | 0.0088 |
| 756 | 1416354_at | Rbmx | RNA binding motif protein, X chromosome | - | 1.2 | 0.0052 |
| 757 | 1443922_at | Rcor3 | REST corepressor 3 | - | 1.2 | 0.0082 |
| 758 | 1424386_at | Reep2 | receptor accessory protein 2 | - | 1.2 | 0.0058 |
| 759 | 1421160_a_at | Rfng | RFNG O-fucosylpeptide 3-beta-N-acetylglucosaminyltransferase | - | 1.2 | 0.0040 |
| 760 | 1419310_s_at | Rfxank | regulatory factor X-associated ankyrin-containing protein | - | 1.2 | 0.0095 |
| 761 | 1425245_a_at | Rgs11 | regulator of G-protein signaling 11 | - | 1.2 | 0.0050 |
| 762 | 1453129_a_at | Rgs12 | regulator of G-protein signaling 12 | - | 1.2 | 0.0053 |
| 763 | 1422238_at | Rhbdd2 | rhomboid domain containing 2 | + | 1.2 | 0.0086 |
| 764 | 1448605_at | Rhoc | ras homolog gene family, member C | - | 1.2 | 0.0077 |
| 765 | 1428710_at | Rit1 | Ras-like without CAAX 1 | + | 1.2 | 0.0023 |
| 766 | 1440526_at | Rltpr | RGD motif, leucine rich repeats, tropomodulin domain and proline-rich containing | - | 1.2 | 0.0090 |
| 767 | 1439583_x_at | Rltpr | RGD motif, leucine rich repeats, tropomodulin domain and proline-rich containing | - | 1.2 | 0.0046 |
| 768 | 1442441_at | Rnasek | ribonuclease, RNase K | - | 1.3 | 0.0054 |
| 769 | 1456108_x_at | Rnf112 | ring finger protein 112 | + | 1.2 | 0.0016 |
| 770 | 1437949_x_at | Rnf114 | ring finger protein 114 | + | 1.2 | 0.0049 |
| 771 | 1431030_a_at | Rnf14 | ring finger protein 14 | - | 1.2 | 0.0033 |
| 772 | 1430527_a_at | Rnf167 | ring finger protein 167 | - | 1.2 | 0.0025 |
| 773 | 1459877_x_at | Rnf185 | ring finger protein 185 | + | 1.2 | 0.0020 |
| 774 | 1455051_at | Rnf31 | ring finger protein 31 | - | 1.2 | 0.0029 |
| 775 | 1436634_at | Robo3 | roundabout homolog 3 (Drosophila) | + | 1.3 | 0.0037 |
| 776 | 1434635_at | Rph3a | rabphilin 3A | - | 1.1 | 0.0077 |
| 777 | 1429759_at | Rps6ka6 | ribosomal protein S6 kinase polypeptide 6 | - | 1.3 | 0.0001 |
| 778 | 1422268_a_at | Rps6kb2 | ribosomal protein S6 kinase, polypeptide 2 | - | 1.3 | 0.0004 |
| 779 | 1434624_x_at | Rps9 | ribosomal protein S9 | + | 1.2 | 0.0084 |
| 780 | 1453253_a_at | Rpusd1 | RNA pseudouridylate synthase domain containing 1 | - | 1.2 | 0.0029 |
| 781 | 1429442_at | Rpusd2 | RNA pseudouridylate synthase domain containing 2 | + | 1.2 | 0.0085 |
| 782 | 1443187_at | Rspo3 | R-spondin 3 homolog (Xenopus laevis) | - | 1.3 | 0.0001 |
| 783 | 1449246_at | Rundc3a | RUN domain containing 3A | - | 1.2 | 0.0070 |
| 784 | 1431137_at | Rusc1 | RUN and SH3 domain containing 1 | - | 1.2 | 0.0047 |
| 785 | 1438017_at | Rusc1 | RUN and SH3 domain containing 1 | - | 1.2 | 0.0092 |
| 786 | 1452533_at | Ryr3 | ryanodine receptor 3 | - | 1.3 | 0.0096 |
| 787 | 1440185_x_at | Sae1 | SUMO1 activating enzyme subunit 1 | + | 1.2 | 0.0007 |
| 788 | 1436306_at | Saps1 | SAPS domain family, member 1 | - | 1.2 | 0.0059 |
| 789 | 1454030_at | Saps3 | SAPS domain family, member 3 | + | 1.3 | 0.0014 |
| 790 | 1445644_at | Sarm1 | sterile alpha and HEAT/Armadillo motif containing 1 | + | 1.2 | 0.0078 |
| 791 | 1434707_at | Sbf1 | SET binding factor 1 | - | 1.2 | 0.0071 |
| 792 | 1443762_s_at | Sbf2 | SET binding factor 2 | + | 1.2 | 0.0082 |
| 793 | 1451190_a_at | Sbk1 | SH3-binding kinase 1 | + | 1.2 | 0.0036 |
| 794 | 1434908_at | Scaf1 | SR-related CTD-associated factor 1 | - | 1.3 | 0.0003 |
| 795 | 1436044_at | Scn7a | sodium channel, voltage-gated, type VII, alpha | - | 1.3 | 0.0017 |
| 796 | 1419975_at | Scp2 | sterol carrier protein 2, liver | + | 1.2 | 0.0064 |
| 797 | 1457310_x_at | Scrt2 | scratch homolog 2, zinc finger protein (Drosophila) | - | 1.2 | 0.0052 |
| 798 | 1418206_at | Sdf2l1 | stromal cell-derived factor 2-like 1 | + | 1.4 | 0.0006 |
| 799 | 1428695_at | Sdr39u1 | short chain dehydrogenase/reductase family 39U, member 1 | - | 1.3 | 0.0013 |
| 800 | 1449202_at | Sema4g | sema domain, immunoglobulin domain (Ig), transmembrane domain (TM) and short cytoplasmic domain, (semaphorin) 4G | - | 1.3 | 0.0009 |
| 801 | 1435963_at | Sema5b | sema domain, seven thrombospondin repeats (type 1 and type 1-like), transmembrane domain (TM) and short cytoplasmic domain, (semaphorin) 5B | - | 1.2 | 0.0057 |
| 802 | 1448790_at | Sema6b | sema domain, transmembrane domain (TM), and cytoplasmic domain, (semaphorin) 6B | - | 1.2 | 0.0068 |
| 803 | 1454610_at | sept7 | septin 7 | - | 1.2 | 0.0048 |
| 804 | 1423866_at | Serpina3k | serine (or cysteine) peptidase inhibitor, clade A, member 3K | + | 1.2 | 0.0040 |
| 805 | 1420885_a_at | Sez6 | seizure related gene 6 | - | 1.2 | 0.0015 |
| 806 | 1456040_at | Sf3b2 | splicing factor 3b, subunit 2 | - | 1.2 | 0.0049 |
| 807 | 1423796_at | Sfpq | splicing factor proline/glutamine rich (polypyrimidine tract binding protein associated) | + | 1.2 | 0.0042 |
| 808 | 1454683_at | Sfrs8 | splicing factor, arginine/serine-rich 8 | + | 1.3 | 0.0080 |
| 809 | 1426423_at | Shmt2 | serine hydroxymethyltransferase 2 (mitochondrial) | - | 1.2 | 0.0086 |
| 810 | 1428493_at | Sipa1l3 | signal-induced proliferation-associated 1 like 3 | + | 1.1 | 0.0062 |
| 811 | 1426441_at | Slc11a2 | solute carrier family 11 (proton-coupled divalent metal ion transporters), member 2 | - | 1.3 | 0.0012 |
| 812 | 1436989_s_at | Slc12a6 | solute carrier family 12, member 6 | + | 1.3 | 0.0005 |
| 813 | 1428986_at | Slc17a7 | solute carrier family 17 (sodium-dependent inorganic phosphate cotransporter), member 7 | - | 1.3 | 0.0063 |
| 814 | 1425950_at | Slc17a9 | solute carrier family 17, member 9 | + | 1.2 | 0.0085 |
| 815 | 1422203_at | Slc18a3 | solute carrier family 18 (vesicular monoamine), member 3 | - | 1.3 | 0.0069 |
| 816 | 1452653_at | Slc25a22 | solute carrier family 25 (mitochondrial carrier, glutamate), member 22 | - | 1.2 | 0.0025 |
| 817 | 1421445_at | Slc26a3 | solute carrier family 26, member 3 | - | 1.2 | 0.0022 |
| 818 | 1416639_at | Slc2a5 | solute carrier family 2 (facilitated glucose transporter), member 5 | + | 1.2 | 0.0026 |
| 819 | 1448741_at | Slc3a1 | solute carrier family 3, member 1 | + | 1.2 | 0.0033 |
| 820 | 1428065_at | Slc44a2 | solute carrier family 44, member 2 | - | 1.2 | 0.0039 |
| 821 | 1436137_at | Slc6a17 | solute carrier family 6 (neurotransmitter transporter), member 17 | - | 1.2 | 0.0043 |
| 822 | 1455760_at | Slc9a5 | solute carrier family 9 (sodium/hydrogen exchanger), member 5 | - | 1.2 | 0.0033 |
| 823 | 1448377_at | Slpi | secretory leukocyte peptidase inhibitor | + | 1.2 | 0.0082 |
| 824 | 1435856_x_at | Smarcb1 | SWI/SNF related, matrix associated, actin dependent regulator of chromatin, subfamily b, member 1 | + | 1.2 | 0.0011 |
| 825 | 1448913_at | Smarcd1 | SWI/SNF related, matrix associated, actin dependent regulator of chromatin, subfamily d, member 1 | - | 1.2 | 0.0082 |
| 826 | 1429531_at | Smpd4 | sphingomyelin phosphodiesterase 4 | + | 1.2 | 0.0046 |
| 827 | 1432043_at | Smu1 | smu-1 suppressor of mec-8 and unc-52 homolog (C. elegans) | - | 1.2 | 0.0096 |
| 828 | 1451722_s_at | Smyd5 | SET and MYND domain containing 5 | - | 1.2 | 0.0025 |
| 829 | 1452713_a_at | Snrnp40 | small nuclear ribonucleoprotein 40 (U5) | + | 1.2 | 0.0021 |
| 830 | 1449840_at | Sntb2 | syntrophin, basic 2 | + | 1.3 | 0.0003 |
| 831 | 1430037_at | Snx27 | sorting nexin family member 27 | + | 1.3 | 0.0009 |
| 832 | 1433449_at | Snx32 | sorting nexin 32 | - | 1.2 | 0.0038 |
| 833 | 1453002_at | Sox11 | SRY-box containing gene 11 | + | 1.3 | 0.0091 |
| 834 | 1453125_at | Sox11 | SRY-box containing gene 11 | + | 1.3 | 0.0086 |
| 835 | 1436790_a_at | Sox11 | SRY-box containing gene 11 | + | 1.3 | 0.0005 |
| 836 | 1433575_at | Sox4 | SRY-box containing gene 4 | + | 1.2 | 0.0071 |
| 837 | 1458332_x_at | Sox4 | SRY-box containing gene 4 | + | 1.3 | 0.0022 |
| 838 | 1432495_at | Sox7 | SRY-box containing gene 7 | + | 1.3 | 0.0037 |
| 839 | 1418180_at | Sp1 | trans-acting transcription factor 1 | + | 1.2 | 0.0061 |
| 840 | 1454656_at | Spata13 | spermatogenesis associated 13 | + | 1.3 | 0.0014 |
| 841 | 1427889_at | Spna2 | spectrin alpha 2 | - | 1.2 | 0.0087 |
| 842 | 1435026_at | Spock2 | sparc/osteonectin, cwcv and kazal-like domains proteoglycan 2 | - | 1.3 | 0.0092 |
| 843 | 1422307_at | Sppl3 | signal peptide peptidase 3 | + | 1.2 | 0.0027 |
| 844 | 1423160_at | Spred1 | sprouty protein with EVH-1 domain 1, related sequence | + | 1.1 | 0.0097 |
| 845 | 1445669_at | Spry4 | sprouty homolog 4 (Drosophila) | + | 1.3 | 0.0017 |
| 846 | 1447806_s_at | Srpk3 | serine/arginine-rich protein specific kinase 3 | + | 1.2 | 0.0095 |
| 847 | 1450045_at | Srrm1 | serine/arginine repetitive matrix 1 | + | 1.2 | 0.0043 |
| 848 | 1419360_a_at | Ss18 | synovial sarcoma translocation, Chromosome 18 | + | 1.1 | 0.0077 |
| 849 | 1419361_at | Ss18 | synovial sarcoma translocation, Chromosome 18 | + | 1.2 | 0.0055 |
| 850 | 1449843_at | St8sia2 | ST8 alpha-N-acetyl-neuraminide alpha-2,8-sialyltransferase 2 | + | 1.2 | 0.0068 |
| 851 | 1423530_at | Stk32c | serine/threonine kinase 32C | + | 1.2 | 0.0099 |
| 852 | 1433186_at | Strada | STE20-related kinase adaptor alpha | + | 1.2 | 0.0067 |
| 853 | 1419913_at | Strap | serine/threonine kinase receptor associated protein | + | 1.5 | 0.0057 |
| 854 | 1416449_x_at | Stxbp2 | syntaxin binding protein 2 | - | 1.2 | 0.0007 |
| 855 | 1425749_at | Stxbp6 | syntaxin binding protein 6 (amisyn) | + | 1.2 | 0.0002 |
| 856 | 1449534_at | Sycp3 | synaptonemal complex protein 3 | + | 1.2 | 0.0098 |
| 857 | 1431798_a_at | Syde1 | synapse defective 1, Rho GTPase, homolog 1 (C. elegans) | - | 1.2 | 0.0003 |
| 858 | 1451484_a_at | Syn1 | synapsin I | - | 1.3 | 0.0028 |
| 859 | 1448280_at | Syp | synaptophysin | - | 1.2 | 0.0019 |
| 860 | 1417708_at | Syt3 | synaptotagmin III | - | 1.2 | 0.0060 |
| 861 | 1422531_at | Syt5 | synaptotagmin V | - | 1.3 | 0.0026 |
| 862 | 1441927_at | Syt7 | synaptotagmin VII | + | 1.2 | 0.0017 |
| 863 | 1431883_at | Tac1 | tachykinin 1 | - | 1.3 | 0.0090 |
| 864 | 1425694_at | Tbx5 | T-box 5 | - | 1.2 | 0.0035 |
| 865 | 1420123_at | Tcta | T-cell leukemia translocation altered gene | - | 1.1 | 0.0094 |
| 866 | 1430912_a_at | Tectb | tectorin beta | - | 1.2 | 0.0055 |
| 867 | 1452264_at | Tenc1 | tensin like C1 domain-containing phosphatase | - | 1.3 | 0.0013 |
| 868 | 1439464_s_at | Tex10 | testis expressed gene 10 | + | 1.2 | 0.0028 |
| 869 | 1418064_at | Tfpt | TCF3 (E2A) fusion partner | - | 1.3 | 0.0013 |
| 870 | 1419314_at | Tinag | tubulointerstitial nephritis antigen | - | 1.2 | 0.0042 |
| 871 | 1436244_a_at | Tle2 | transducin-like enhancer of split 2, homolog of Drosophila E(spl) | - | 1.3 | 0.0008 |
| 872 | 1451996_at | Tm2d1 | TM2 domain containing 1 | + | 1.3 | 0.0095 |
| 873 | 1425079_at | Tm6sf2 | transmembrane 6 superfamily member 2 | + | 1.3 | 0.0038 |
| 874 | 1438504_x_at | Tm7sf3 | transmembrane 7 superfamily member 3 | + | 1.2 | 0.0076 |
| 875 | 1430818_at | Tmc1 | transmembrane channel-like gene family 1 | + | 1.2 | 0.0090 |
| 876 | 1458372_at | Tmc8 | transmembrane channel-like gene family 8 | + | 1.2 | 0.0033 |
| 877 | 1441890_x_at | Tmeff1 | transmembrane protein with EGF-like and two follistatin-like domains 1 | + | 1.2 | 0.0026 |
| 878 | 1456057_x_at | Tmem109 | transmembrane protein 109 | + | 1.2 | 0.0090 |
| 879 | 1441880_x_at | Tmem149 | transmembrane protein 149 | + | 1.2 | 0.0029 |
| 880 | 1428899_at | Tmem182 | transmembrane protein 182 | - | 1.2 | 0.0047 |
| 881 | 1452053_a_at | Tmem33 | transmembrane protein 33 | + | 1.2 | 0.0051 |
| 882 | 1425568_a_at | Tmem33 | transmembrane protein 33 | + | 1.2 | 0.0053 |
| 883 | 1449677_s_at | Tmem38b | transmembrane protein 38B | + | 1.2 | 0.0019 |
| 884 | 1452825_at | Tmem59l | transmembrane protein 59-like | - | 1.2 | 0.0028 |
| 885 | 1432008_at | Tmem86b | transmembrane protein 86B | + | 1.3 | 0.0065 |
| 886 | 1458305_at | Tmtc3 | transmembrane and tetratricopeptide repeat containing 3 | - | 1.2 | 0.0007 |
| 887 | 1416342_at | Tnc | tenascin C | - | 1.2 | 0.0033 |
| 888 | 1422231_a_at | Tnfrsf25 | tumor necrosis factor receptor superfamily, member 25 | + | 1.4 | 0.0012 |
| 889 | 1460469_at | Tnfrsf9 | tumor necrosis factor receptor superfamily, member 9 | + | 1.2 | 0.0022 |
| 890 | 1431188_a_at | Tom1 | target of myb1 homolog (chicken) | - | 1.2 | 0.0005 |
| 891 | 1439043_at | Tra2a | transformer 2 alpha homolog (Drosophila) | + | 1.6 | 0.0008 |
| 892 | 1449571_at | Trhr | thyrotropin releasing hormone receptor | - | 1.5 | 0.0091 |
| 893 | 1451663_a_at | Trim3 | tripartite motif-containing 3 | - | 1.2 | 0.0019 |
| 894 | 1416118_at | Trim59 | tripartite motif-containing 59 | + | 1.2 | 0.0027 |
| 895 | 1453599_at | Trim71 | tripartite motif-containing 71 | + | 1.2 | 0.0047 |
| 896 | 1459581_at | Trp63 | transformation related protein 63 | + | 1.2 | 0.0044 |
| 897 | 1449431_at | Trpc6 | transient receptor potential cation channel, subfamily C, member 6 | + | 1.2 | 0.0080 |
| 898 | 1427642_at | Trpm6 | transient receptor potential cation channel, subfamily M, member 6 | + | 1.2 | 0.0061 |
| 899 | 1434849_at | Tspyl2 | TSPY-like 2 | - | 1.2 | 0.0022 |
| 900 | 1433460_at | Ttc7b | tetratricopeptide repeat domain 7B | - | 1.2 | 0.0062 |
| 901 | 1427284_a_at | Ttpa | tocopherol (alpha) transfer protein | - | 1.2 | 0.0093 |
| 902 | 1427838_at | Tubb2a | tubulin, beta 2A | + | 1.2 | 0.0027 |
| 903 | 1423221_at | Tubb4 | tubulin, beta 4 | - | 1.3 | 0.0051 |
| 904 | 1439440_x_at | Twf2 | twinfilin, actin-binding protein, homolog 2 (Drosophila) | + | 1.2 | 0.0043 |
| 905 | 1439184_s_at | Txndc17 | thioredoxin domain containing 17 | + | 1.2 | 0.0010 |
| 906 | 1448116_at | Uba1 | ubiquitin-like modifier activating enzyme 1 | - | 1.2 | 0.0025 |
| 907 | 1437278_a_at | Uba2 | ubiquitin-like modifier activating enzyme 2 | + | 1.1 | 0.0055 |
| 908 | 1454373_x_at | Ubc | ubiquitin C | + | 1.5 | 0.0029 |
| 909 | 1452054_at | Ube2w | ubiquitin-conjugating enzyme E2W (putative) | + | 1.2 | 0.0032 |
| 910 | 1448692_at | Ubqln4 | ubiquilin 4 | - | 1.2 | 0.0009 |
| 911 | 1448260_at | Uchl1 | ubiquitin carboxy-terminal hydrolase L1 | - | 1.2 | 0.0014 |
| 912 | 1439028_at | Ufm1 | ubiquitin-fold modifier 1 | + | 1.3 | 0.0023 |
| 913 | 1439211_at | Umodl1 | uromodulin-like 1 | - | 1.2 | 0.0094 |
| 914 | 1426681_at | Unk | unkempt homolog (Drosophila) | - | 1.2 | 0.0061 |
| 915 | 1425023_at | Usp3 | ubiquitin specific peptidase 3 | - | 1.2 | 0.0083 |
| 916 | 1453162_at | Utp11l | UTP11-like, U3 small nucleolar ribonucleoprotein, (yeast) | + | 1.2 | 0.0063 |
| 917 | 1421863_at | Vamp1 | vesicle-associated membrane protein 1 | - | 1.2 | 0.0082 |
| 918 | 1448472_at | Vars | valyl-tRNA synthetase | - | 1.2 | 0.0005 |
| 919 | 1420909_at | Vegfa | vascular endothelial growth factor A | + | 1.2 | 0.0024 |
| 920 | 1419417_at | Vegfc | vascular endothelial growth factor C | - | 1.2 | 0.0068 |
| 921 | 1438881_at | Vezt | vezatin, adherens junctions transmembrane protein | - | 1.2 | 0.0096 |
| 922 | 1452489_at | Vps11 | vacuolar protein sorting 11 (yeast) | + | 1.2 | 0.0072 |
| 923 | 1450055_at | Vsnl1 | visinin-like 1 | - | 1.2 | 0.0066 |
| 924 | 1451230_a_at | Wbp5 | WW domain binding protein 5 | - | 1.1 | 0.0086 |
| 925 | 1456645_at | Wdr25 | WD repeat domain 25 | - | 1.2 | 0.0090 |
| 926 | 1452392_a_at | Wipi1 | WD repeat domain, phosphoinositide interacting 1 | + | 1.3 | 0.0056 |
| 927 | 1457499_at | Wwp2 | WW domain containing E3 ubiquitin protein ligase 2 | + | 1.2 | 0.0075 |
| 928 | 1437155_a_at | Wwtr1 | WW domain containing transcription regulator 1 | + | 1.2 | 0.0025 |
| 929 | 1420012_at | Xbp1 | X-box binding protein 1 | + | 1.6 | 0.0002 |
| 930 | 1439411_a_at | Xpo7 | exportin 7 | + | 1.2 | 0.0098 |
| 931 | 1438839_a_at | Ywhae | tyrosine 3-monooxygenase/tryptophan 5-monooxygenase activation protein, epsilon polypeptide | - | 1.2 | 0.0078 |
| 932 | 1436981_a_at | Ywhaz | tyrosine 3-monooxygenase/tryptophan 5-monooxygenase activation protein, zeta polypeptide | + | 1.3 | 0.0053 |
| 933 | 1439005_x_at | Ywhaz | tyrosine 3-monooxygenase/tryptophan 5-monooxygenase activation protein, zeta polypeptide | + | 1.4 | 0.0006 |
| 934 | 1422570_at | Yy1 | YY1 transcription factor | - | 1.2 | 0.0071 |
| 935 | 1453175_at | Zbtb25 | zinc finger and BTB domain containing 25 | - | 1.2 | 0.0030 |
| 936 | 1426999_at | Zc3h14 | zinc finger CCCH type containing 14 | - | 1.2 | 0.0060 |
| 937 | 1437355_at | Zcchc5 | zinc finger, CCHC domain containing 5 | - | 1.3 | 0.0084 |
| 938 | 1433748_at | Zdhhc18 | zinc finger, DHHC domain containing 18 | + | 1.2 | 0.0100 |
| 939 | 1416084_at | Zfand5 | zinc finger, AN1-type domain 5 | - | 1.2 | 0.0087 |
| 940 | 1425010_at | Zfp119 | zinc finger protein 119 | - | 1.2 | 0.0062 |
| 941 | 1428170_at | Zfp180 | zinc finger protein 180 | + | 1.2 | 0.0026 |
| 942 | 1433953_at | Zfp277 | zinc finger protein 277 | - | 1.2 | 0.0042 |
| 943 | 1455638_at | Zfp319 | zinc finger protein 319 | + | 1.2 | 0.0069 |
| 944 | 1420110_s_at | Zfp334 | zinc finger protein 334 | - | 1.2 | 0.0077 |
| 945 | 1422528_a_at | Zfp36l1 | zinc finger protein 36, C3H type-like 1 | + | 1.3 | 0.0001 |
| 946 | 1421139_a_at | Zfp386 | zinc finger protein 386 (Kruppel-like) | - | 1.3 | 0.0093 |
| 947 | 1436081_a_at | Zfp414 | zinc finger protein 414 | - | 1.3 | 0.0023 |
| 948 | 1425058_at | Zfp472 | zinc finger protein 472 | - | 1.2 | 0.0033 |
| 949 | 1429634_at | Zfp580 | zinc finger protein 580 | - | 1.2 | 0.0029 |
| 950 | 1449691_at | Zfp644 | zinc finger protein 644 | + | 1.3 | 0.0042 |
| 951 | 1434720_at | Zfp652 | zinc finger protein 652 | + | 1.2 | 0.0015 |
| 952 | 1454986_at | Zfp668 | zinc finger protein 668 | - | 1.2 | 0.0098 |
| 953 | 1428687_at | Zfp687 | zinc finger protein 687 | - | 1.1 | 0.0098 |
| 954 | 1451902_at | Zfp758 | zinc finger protein 758 | - | 1.3 | 0.0064 |
| 955 | 1452623_at | Zfp759 | zinc finger protein 759 | - | 1.2 | 0.0013 |
| 956 | 1426292_at | Zfp790 | zinc finger protein 790 | + | 1.1 | 0.0096 |
| 957 | 1427499_at | Zfp81 | zinc finger protein 81 | - | 1.2 | 0.0077 |
| 958 | 1438079_at | Zfp867 | zinc finger protein 867 | - | 1.2 | 0.0009 |
| 959 | 1425531_at | Znhit1 | zinc finger, HIT domain containing 1 | - | 1.2 | 0.0080 |
| 960 | 1433869_at | Zxdc | ZXD family zinc finger C | - | 1.1 | 0.0055 |
