# Supplementary material for: Differential Effects of Prenatal Stress in 5-Htt Deficient Mice: Towards Molecular Mechanisms of Gene × Environment Interactions
Source: PLoS One. 2011 Aug 12;6(8):e22715. doi: 10.1371/journal.pone.0022715 (PMC3155516; doi:10.1371/journal.pone.0022715)
Supplement: Text S3 — (DOC) [file pone.0022715.s003.doc]

**Supplemental material 3**

**Genes regulated at the gene × environment (G×E) level (full list).**

ID: Affymetrix ID; FC: fold-change in mRNA expression. Genes have been ordered alphabetically.

| **#** | **ID** | **SYMBOL** | **GENENAME** | **FC** | **P-Value** |
| --- | --- | --- | --- | --- | --- |
| 1 | 1457715_at | 1010001B22Rik | RIKEN cDNA 1010001B22 gene | 2.3 | 0.006 |
| 2 | 1454286_at | 1110004M10Rik | RIKEN cDNA 1110004M10 gene | 1.6 | 0.003 |
| 3 | 1430012_at | 1110050K14Rik | RIKEN cDNA 1110050K14 gene | 1.4 | 0.008 |
| 4 | 1438288_x_at | 1110059G02Rik | RIKEN cDNA 1110059G02 gene | 1.6 | 0.004 |
| 5 | 1422722_at | 1700001K19Rik | RIKEN cDNA 1700001K19 gene | 1.8 | 0.004 |
| 6 | 1451061_at | 1700018B24Rik | enhancer of rudimentary homolog pseudogene | 1.6 | 0.007 |
| 7 | 1442779_at | 1700071K01Rik | RIKEN cDNA 1700071K01 gene | 1.9 | 0.002 |
| 8 | 1447798_at | 1700123O21Rik | RIKEN cDNA 1700123O21 gene | 2.7 | 0.003 |
| 9 | 1430612_at | 1810033B17Rik | RIKEN cDNA 1810033B17 gene | 1.4 | 0.007 |
| 10 | 1430280_at | 1810062G17Rik | RIKEN cDNA 1810062G17 gene | 1.6 | 0.006 |
| 11 | 1447576_at | 2010001K21Rik | RIKEN cDNA 2010001K21 gene | 1.9 | 0.000 |
| 12 | 1430878_at | 2210406H18Rik | RIKEN cDNA 2210406H18 gene | 1.6 | 0.002 |
| 13 | 1453174_at | 2310076G13Rik | RIKEN cDNA 2310076G13 gene | 1.7 | 0.009 |
| 14 | 1430195_at | 2810043O03Rik | RIKEN cDNA 2810043O03 gene | 1.5 | 0.006 |
| 15 | 1444703_at | 2810403D21Rik | RIKEN cDNA 2810403D21 gene | 1.4 | 0.003 |
| 16 | 1433266_at | 2810416A17Rik | RIKEN cDNA 2810416A17 gene | 1.9 | 0.004 |
| 17 | 1432824_at | 2900018N21Rik | RIKEN cDNA 2900018N21 gene | 1.7 | 0.008 |
| 18 | 1433387_at | 2900022M07Rik | RIKEN cDNA 2900022M07 gene | 2.5 | 0.000 |
| 19 | 1431703_at | 2900027M19Rik | RIKEN cDNA 2900027M19 gene | 2.1 | 0.007 |
| 20 | 1432944_at | 2900046L07Rik | RIKEN cDNA 2900046L07 gene | 1.6 | 0.008 |
| 21 | 1453456_at | 2900084O13Rik | RIKEN cDNA 2900084O13 gene | 2.0 | 0.003 |
| 22 | 1454478_at | 3100002H20Rik | RIKEN cDNA 3100002H20 gene | 1.5 | 0.007 |
| 23 | 1432686_at | 4833406M21Rik | RIKEN cDNA 4833406M21 gene | 1.7 | 0.008 |
| 24 | 1430622_at | 4833423F13Rik | RIKEN cDNA 4833423F13 gene | 1.6 | 0.010 |
| 25 | 1430467_at | 4921511H03Rik | RIKEN cDNA 4921511H03 gene | 1.5 | 0.009 |
| 26 | 1430722_at | 4921515J06Rik | RIKEN cDNA 4921515J06 gene | 1.3 | 0.009 |
| 27 | 1440467_at | 4922501C03Rik | RIKEN cDNA 4922501C03 gene | 1.7 | 0.003 |
| 28 | 1440349_at | 4930420K17Rik | RIKEN cDNA 4930420K17 gene | 1.6 | 0.000 |
| 29 | 1433234_at | 4930424E08Rik | RIKEN cDNA 4930424E08 gene | 1.4 | 0.008 |
| 30 | 1431954_x_at | 4930431C11Rik | RIKEN cDNA 4930431C11 gene | 1.7 | 0.002 |
| 31 | 1431628_at | 4930435H24Rik | RIKEN cDNA 4930435H24 gene | 1.5 | 0.005 |
| 32 | 1442152_at | 4930513N10Rik | RIKEN cDNA 4930513N10 gene | 1.6 | 0.002 |
| 33 | 1433320_at | 4930519N06Rik | RIKEN cDNA 4930519N06 gene | 1.6 | 0.005 |
| 34 | 1440783_at | 4930529M08Rik | RIKEN cDNA 4930529M08 gene | 1.8 | 0.009 |
| 35 | 1431660_at | 4930564D02Rik | RIKEN cDNA 4930564D02 gene | 1.5 | 0.005 |
| 36 | 1439684_at | 4930570G19Rik | RIKEN cDNA 4930570G19 gene | 1.8 | 0.000 |
| 37 | 1430175_at | 4930588G05Rik | RIKEN cDNA 4930588G05 gene | 1.6 | 0.004 |
| 38 | 1436219_at | 4933403F05Rik | RIKEN cDNA 4933403F05 gene | 1.4 | 0.009 |
| 39 | 1453893_at | 4933412O06Rik | RIKEN cDNA 4933412O06 gene | 1.4 | 0.003 |
| 40 | 1431571_at | 4933437I04Rik | RIKEN cDNA 4933437I04 gene | 1.9 | 0.002 |
| 41 | 1427065_at | 4933439F18Rik | RIKEN cDNA 4933439F18 gene | 1.6 | 0.002 |
| 42 | 1431248_at | 5031426D15Rik | RIKEN cDNA 5031426D15 gene | 1.9 | 0.007 |
| 43 | 1431473_at | 5330423I11Rik | RIKEN cDNA 5330423I11 gene | 1.7 | 0.003 |
| 44 | 1439179_a_at | 5830405N20Rik | RIKEN cDNA 5830405N20 gene | 1.6 | 0.010 |
| 45 | 1456296_at | 5830418K08Rik | RIKEN cDNA 5830418K08 gene | 1.8 | 0.009 |
| 46 | 1432696_at | 5830431M20Rik | RIKEN cDNA 5830431M20 gene | 1.6 | 0.001 |
| 47 | 1439512_at | 5830444B04Rik | RIKEN cDNA 5830444B04 gene | 1.6 | 0.009 |
| 48 | 1430585_at | 5930436O19Rik | RIKEN cDNA 5930436O19 gene | 1.4 | 0.002 |
| 49 | 1433202_at | 6030400A10Rik | RIKEN cDNA 6030400A10 gene | 1.6 | 0.009 |
| 50 | 1431242_at | 6330575P09Rik | RIKEN cDNA 6330575P09 gene | 1.5 | 0.003 |
| 51 | 1434797_at | 6720469N11Rik | RIKEN cDNA 6720469N11 gene | 1.6 | 0.004 |
| 52 | 1433184_at | 6720477C19Rik | RIKEN cDNA 6720477C19 gene | 1.9 | 0.009 |
| 53 | 1440542_at | 7420416P09Rik | RIKEN cDNA 7420416P09 gene | 1.3 | 0.009 |
| 54 | 1430877_at | 8030425K09Rik | RIKEN cDNA 8030425K09 gene | 1.6 | 0.005 |
| 55 | 1444797_at | 8030474K03Rik | RIKEN cDNA 8030474K03 gene | 1.6 | 0.002 |
| 56 | 1453638_at | 9030420J04Rik | RIKEN cDNA 9030420J04 gene | 1.4 | 0.005 |
| 57 | 1433002_at | 9030613N10Rik | RIKEN cDNA 9030613N10 gene | 1.7 | 0.005 |
| 58 | 1433261_at | 9430052A13Rik | RIKEN cDNA 9430052A13 gene | 1.7 | 0.007 |
| 59 | 1431491_at | 9430087N24Rik | RIKEN cDNA 9430087N24 gene | 1.6 | 0.010 |
| 60 | 1433195_at | 9530006O14Rik | RIKEN cDNA 9530006O14 gene | 1.6 | 0.003 |
| 61 | 1458113_at | 9530019H20Rik | RIKEN cDNA 9530019H20 gene | 1.5 | 0.002 |
| 62 | 1441179_at | 9530020O07Rik | RIKEN cDNA 9530020O07 gene | 1.5 | 0.004 |
| 63 | 1445167_at | 9630001P10Rik | RIKEN cDNA 9630001P10 gene | 1.5 | 0.004 |
| 64 | 1458123_at | 9630002A11Rik | RIKEN cDNA 9630002A11 gene | 1.9 | 0.002 |
| 65 | 1443127_at | 9630021D06Rik | RIKEN cDNA 9630021D06 gene | 2.3 | 0.000 |
| 66 | 1456634_at | 9830001H06Rik | RIKEN cDNA 9830001H06 gene | 1.5 | 0.003 |
| 67 | 1440671_at | A130012E19Rik | RIKEN cDNA A130012E19 gene | 1.5 | 0.002 |
| 68 | 1443457_at | A230055J12Rik | RIKEN cDNA A230055J12 gene | 1.5 | 0.006 |
| 69 | 1454278_at | A430105D02Rik | RIKEN cDNA A430105D02 gene | 1.6 | 0.005 |
| 70 | 1440884_s_at | A530047J11Rik | RIKEN cDNA A530047J11 gene | 1.7 | 0.002 |
| 71 | 1455203_at | A930003A15Rik | RIKEN cDNA A930003A15 gene | 1.4 | 0.007 |
| 72 | 1459349_at | A930011G23Rik | RIKEN cDNA A930011G23 gene | 1.5 | 0.002 |
| 73 | 1459391_at | Abca5 | ATP-binding cassette, sub-family A (ABC1), member 5 | 1.7 | 0.004 |
| 74 | 1456812_at | Abcd2 | ATP-binding cassette, sub-family D (ALD), member 2 | 1.6 | 0.002 |
| 75 | 1453474_at | Abhd15 | abhydrolase domain containing 15 | 1.6 | 0.009 |
| 76 | 1446433_at | Acbd5 | acyl-Coenzyme A binding domain containing 5 | 1.5 | 0.003 |
| 77 | 1441975_at | Acpp | acid phosphatase, prostate | 1.6 | 0.006 |
| 78 | 1437382_at | Acvr2a | activin receptor IIA | 1.5 | 0.004 |
| 79 | 1448539_a_at | Acy3 | aspartoacylase (aminoacylase) 3 | 1.5 | 0.003 |
| 80 | 1452595_at | Adamts4 | a disintegrin-like and metallopeptidase (reprolysin type) with thrombospondin type 1 motif, 4 | 1.6 | 0.001 |
| 81 | 1451932_a_at | Adamtsl4 | ADAMTS-like 4 | 1.4 | 0.007 |
| 82 | 1416225_at | Adh1 | alcohol dehydrogenase 1 (class I) | 1.7 | 0.004 |
| 83 | 1438759_x_at | Adi1 | acireductone dioxygenase 1 | 1.4 | 0.009 |
| 84 | 1446463_at | Adnp2 | ADNP homeobox 2 | 1.5 | 0.004 |
| 85 | 1435719_at | AI448984 | expressed sequence AI448984 | 1.7 | 0.008 |
| 86 | 1438716_at | AI451617 | expressed sequence AI451617 | 1.9 | 0.003 |
| 87 | 1460003_at | AI956758 | expressed sequence AI956758 | 2.1 | 0.003 |
| 88 | 1442703_at | AK220484 | cDNA sequence AK220484 | 1.5 | 0.004 |
| 89 | 1439066_at | Angpt1 | angiopoietin 1 | 1.8 | 0.003 |
| 90 | 1450085_at | Angptl2 | angiopoietin-like 2 | 1.4 | 0.005 |
| 91 | 1447259_at | Ank3 | ankyrin 3, epithelial | 1.6 | 0.005 |
| 92 | 1437768_at | Ankib1 | ankyrin repeat and IBR domain containing 1 | 1.6 | 0.001 |
| 93 | 1443887_at | Ankrd13c | ankyrin repeat domain 13c | 1.6 | 0.003 |
| 94 | 1451837_at | Ap3b2 | adaptor-related protein complex 3, beta 2 subunit | 1.8 | 0.004 |
| 95 | 1443112_at | Api5 | apoptosis inhibitor 5 | 1.7 | 0.006 |
| 96 | 1418069_at | Apoc2 | apolipoprotein C-II | 1.5 | 0.010 |
| 97 | 1418853_at | Apon | apolipoprotein N | 2.2 | 0.001 |
| 98 | 1450460_at | Aqp3 | aquaporin 3 | 1.5 | 0.002 |
| 99 | 1419549_at | Arg1 | arginase, liver | 1.4 | 0.007 |
| 100 | 1457410_at | Arhgap5 | Rho GTPase activating protein 5 | 1.6 | 0.005 |
| 101 | 1451867_x_at | Arhgap6 | Rho GTPase activating protein 6 | 2.2 | 0.001 |
| 102 | 1441022_at | Arih1 | ariadne ubiquitin-conjugating enzyme E2 binding protein homolog 1 (Drosophila) | 1.4 | 0.007 |
| 103 | 1441190_at | Arpc5l | actin related protein 2/3 complex, subunit 5-like | 1.6 | 0.009 |
| 104 | 1438921_at | Atr | ataxia telangiectasia and Rad3 related | 1.9 | 0.006 |
| 105 | 1458945_at | AU015148 | expressed sequence AU015148 | 1.6 | 0.005 |
| 106 | 1459155_at | AU015696 | expressed sequence AU015696 | 1.4 | 0.006 |
| 107 | 1424266_s_at | AU018778 | expressed sequence AU018778 | 1.6 | 0.002 |
| 108 | 1443399_at | AU021001 | expressed sequence AU021001 | 1.8 | 0.000 |
| 109 | 1418604_at | Avpr1a | arginine vasopressin receptor 1A | 1.9 | 0.001 |
| 110 | 1430000_at | B230117O15Rik | RIKEN cDNA B230117O15 gene | 1.6 | 0.001 |
| 111 | 1442028_at | B4galnt2 | beta-1,4-N-acetyl-galactosaminyl transferase 2 | 1.5 | 0.005 |
| 112 | 1440781_at | B830007D08Rik | RIKEN cDNA B830007D08 gene | 2.5 | 0.005 |
| 113 | 1458376_at | B930025B16Rik | RIKEN cDNA B930025B16 gene | 1.9 | 0.002 |
| 114 | 1446681_at | BB086117 | expressed sequence BB086117 | 1.6 | 0.002 |
| 115 | 1436585_at | BB182297 | expressed sequence BB182297 | 1.7 | 0.001 |
| 116 | 1427041_at | BC013712 | cDNA sequence BC013712 | 1.5 | 0.004 |
| 117 | 1437264_at | BC051142 | cDNA sequence BC051142 | 1.8 | 0.001 |
| 118 | 1424814_a_at | Bcl2l14 | BCL2-like 14 (apoptosis facilitator) | 2.1 | 0.005 |
| 119 | 1422745_at | Bicd2 | bicaudal D homolog 2 (Drosophila) | 1.5 | 0.007 |
| 120 | 1420683_at | Bnipl | BCL2/adenovirus E1B 19kD interacting protein like | 1.3 | 0.006 |
| 121 | 1426129_at | Brms1 | breast cancer metastasis-suppressor 1 | 1.5 | 0.004 |
| 122 | 1439329_a_at | Brsk2 | BR serine/threonine kinase 2 | 1.9 | 0.009 |
| 123 | 1435953_at | Btaf1 | BTAF1 RNA polymerase II, B-TFIID transcription factor-associated, (Mot1 homolog, S. cerevisiae) | 1.4 | 0.008 |
| 124 | 1454342_at | C030007D22Rik | RIKEN cDNA C030007D22 gene | 1.6 | 0.008 |
| 125 | 1432200_at | C030044M21Rik | RIKEN cDNA C030044M21 gene | 1.6 | 0.002 |
| 126 | 1438905_x_at | C030046I01Rik | RIKEN cDNA C030046I01 gene | 1.6 | 0.009 |
| 127 | 1416051_at | C2 | complement component 2 (within H-2S) | 1.4 | 0.004 |
| 128 | 1444340_at | C230066G23Rik | RIKEN cDNA C230066G23 gene | 1.5 | 0.005 |
| 129 | 1457656_s_at | C230085N15Rik | RIKEN cDNA C230085N15 gene | 1.9 | 0.001 |
| 130 | 1438125_at | C230085N15Rik | RIKEN cDNA C230085N15 gene | 1.4 | 0.005 |
| 131 | 1432423_a_at | C530008M17Rik | RIKEN cDNA C530008M17 gene | 1.4 | 0.006 |
| 132 | 1441394_at | C76554 | expressed sequence C76554 | 1.6 | 0.004 |
| 133 | 1444445_at | C77648 | expressed sequence C77648 | 1.5 | 0.007 |
| 134 | 1447182_at | C77815 | expressed sequence C77815 | 2.1 | 0.000 |
| 135 | 1442566_at | C78878 | expressed sequence C78878 | 1.5 | 0.001 |
| 136 | 1447507_at | C80406 | expressed sequence C80406 | 1.5 | 0.006 |
| 137 | 1441477_at | Calu | calumenin | 1.5 | 0.003 |
| 138 | 1422659_at | Camk2d | calcium/calmodulin-dependent protein kinase II, delta | 1.6 | 0.009 |
| 139 | 1439843_at | Camk4 | calcium/calmodulin-dependent protein kinase IV | 1.8 | 0.006 |
| 140 | 1432628_at | Cbx3 | chromobox homolog 3 (Drosophila HP1 gamma) | 1.9 | 0.003 |
| 141 | 1441886_at | Ccdc79 | coiled-coil domain containing 79 | 1.5 | 0.004 |
| 142 | 1436789_at | Ccnjl | cyclin J-like | 1.5 | 0.006 |
| 143 | 1421188_at | Ccr2 | chemokine (C-C motif) receptor 2 | 1.5 | 0.003 |
| 144 | 1427736_a_at | Ccrl2 | chemokine (C-C motif) receptor-like 2 | 1.5 | 0.004 |
| 145 | 1423760_at | Cd44 | CD44 antigen | 1.7 | 0.004 |
| 146 | 1427095_at | Cdcp1 | CUB domain containing protein 1 | 1.5 | 0.003 |
| 147 | 1460045_at | Cdh7 | cadherin 7, type 2 | 1.8 | 0.001 |
| 148 | 1419497_at | Cdkn1b | cyclin-dependent kinase inhibitor 1B | 1.7 | 0.002 |
| 149 | 1425912_at | Cep164 | centrosomal protein 164 | 1.5 | 0.005 |
| 150 | 1425642_at | Cep290 | centrosomal protein 290 | 1.8 | 0.009 |
| 151 | 1419320_at | Chst5 | carbohydrate (N-acetylglucosamine 6-O) sulfotransferase 5 | 1.6 | 0.001 |
| 152 | 1425200_at | Clcc1 | chloride channel CLIC-like 1 | 1.4 | 0.005 |
| 153 | 1421698_a_at | Col19a1 | collagen, type XIX, alpha 1 | 1.6 | 0.002 |
| 154 | 1440911_at | Col23a1 | collagen, type XXIII, alpha 1 | 1.7 | 0.007 |
| 155 | 1419703_at | Col5a3 | collagen, type V, alpha 3 | 1.6 | 0.005 |
| 156 | 1421373_at | Cox4i2 | cytochrome c oxidase subunit IV isoform 2 | 1.5 | 0.002 |
| 157 | 1419960_at | Cphx | cytoplasmic polyadenylated homeobox | 1.6 | 0.004 |
| 158 | 1443909_at | Cstf3 | cleavage stimulation factor, 3' pre-RNA, subunit 3 | 1.8 | 0.004 |
| 159 | 1422680_at | Ctr9 | Ctr9, Paf1/RNA polymerase II complex component, homolog (S. cerevisiae) | 1.4 | 0.003 |
| 160 | 1417590_at | Cyp27a1 | cytochrome P450, family 27, subfamily a, polypeptide 1 | 1.5 | 0.004 |
| 161 | 1438581_at | Cytsa | cytospin A | 1.6 | 0.005 |
| 162 | 1457304_at | D13Ertd787e | DNA segment, Chr 13, ERATO Doi 787, expressed | 1.7 | 0.005 |
| 163 | 1457004_at | D15Wsu169e | DNA segment, Chr 15, Wayne State University 169, expressed | 1.7 | 0.006 |
| 164 | 1446564_at | D18Ertd169e | DNA segment, Chr 18, ERATO Doi 169, expressed | 2.0 | 0.002 |
| 165 | 1441229_at | D230019N24Rik | RIKEN cDNA D230019N24 gene | 1.5 | 0.006 |
| 166 | 1441558_at | D230044B12Rik | RIKEN cDNA D230044B12 gene | 1.7 | 0.002 |
| 167 | 1438222_at | D2Ertd612e | DNA segment, Chr 2, ERATO Doi 612, expressed | 1.8 | 0.008 |
| 168 | 1439691_at | D5Ertd579e | DNA segment, Chr 5, ERATO Doi 579, expressed | 1.4 | 0.004 |
| 169 | 1438788_at | D5Wsu152e | DNA segment, Chr 5, Wayne State University 152, expressed | 1.6 | 0.004 |
| 170 | 1444292_at | D7Ertd143e | DNA segment, Chr 7, ERATO Doi 143, expressed | 1.4 | 0.004 |
| 171 | 1424860_at | D930016D06Rik | RIKEN cDNA D930016D06 gene | 1.7 | 0.007 |
| 172 | 1424861_at | D930016D06Rik | RIKEN cDNA D930016D06 gene | 1.6 | 0.009 |
| 173 | 1457012_at | Dbx1 | developing brain homeobox 1 | 1.4 | 0.007 |
| 174 | 1446190_at | Dclk1 | doublecortin-like kinase 1 | 1.8 | 0.007 |
| 175 | 1458361_at | Dclre1c | DNA cross-link repair 1C, PSO2 homolog (S. cerevisiae) | 1.7 | 0.001 |
| 176 | 1458614_at | Dcp1b | DCP1 decapping enzyme homolog b (S. cerevisiae) | 1.6 | 0.001 |
| 177 | 1418139_at | Dcx | doublecortin | 1.4 | 0.008 |
| 178 | 1445798_at | Dlg1 | discs, large homolog 1 (Drosophila) | 1.7 | 0.001 |
| 179 | 1440639_at | Dlgap1 | discs, large (Drosophila) homolog-associated protein 1 | 1.4 | 0.008 |
| 180 | 1420335_at | Dmc1 | DMC1 dosage suppressor of mck1 homolog, meiosis-specific homologous recombination (yeast) | 1.4 | 0.003 |
| 181 | 1420221_at | Dnajc21 | DnaJ (Hsp40) homolog, subfamily C, member 21 | 1.8 | 0.001 |
| 182 | 1452638_s_at | Dnm1l | dynamin 1-like | 1.6 | 0.003 |
| 183 | 1449052_a_at | Dnmt3b | DNA methyltransferase 3B | 1.5 | 0.004 |
| 184 | 1418351_a_at | Dnmt3b | DNA methyltransferase 3B | 1.5 | 0.007 |
| 185 | 1436661_at | Dpp10 | dipeptidylpeptidase 10 | 1.6 | 0.003 |
| 186 | 1437272_at | Dpy19l2 | dpy-19-like 2 (C. elegans) | 1.5 | 0.001 |
| 187 | 1434534_at | Dsc3 | desmocollin 3 | 1.7 | 0.003 |
| 188 | 1445710_x_at | Duxbl | double homeobox B-like | 1.8 | 0.009 |
| 189 | 1454393_at | Dzip3 | DAZ interacting protein 3, zinc finger | 1.5 | 0.006 |
| 190 | 1430386_at | E030024N20Rik | peptidylprolyl isomerase A pseudogene 8 | 1.4 | 0.009 |
| 191 | 1439571_at | E230008J23Rik | RIKEN cDNA E230008J23 gene | 1.7 | 0.005 |
| 192 | 1424561_at | Ece2 | endothelin converting enzyme 2 | 1.5 | 0.001 |
| 193 | 1421900_at | Eif2ak1 | eukaryotic translation initiation factor 2 alpha kinase 1 | 1.6 | 0.002 |
| 194 | 1420491_at | Eif2s1 | eukaryotic translation initiation factor 2, subunit 1 alpha | 1.7 | 0.007 |
| 195 | 1434489_at | Elmo3 | engulfment and cell motility 3, ced-12 homolog (C. elegans) | 1.6 | 0.006 |
| 196 | 1447857_at | Enox1 | ecto-NOX disulfide-thiol exchanger 1 | 1.6 | 0.006 |
| 197 | 1419276_at | Enpp1 | ectonucleotide pyrophosphatase/phosphodiesterase 1 | 1.6 | 0.009 |
| 198 | 1421406_at | Entpd7 | ectonucleoside triphosphate diphosphohydrolase 7 | 1.4 | 0.008 |
| 199 | 1444150_at | Epb4.1 | erythrocyte protein band 4.1 | 2.2 | 0.001 |
| 200 | 1456481_at | Esyt3 | extended synaptotagmin-like protein 3 | 1.6 | 0.001 |
| 201 | 1449305_at | F10 | coagulation factor X | 1.4 | 0.008 |
| 202 | 1436374_x_at | F11r | F11 receptor | 1.8 | 0.004 |
| 203 | 1421595_at | Fam184b | family with sequence similarity 184, member B | 1.4 | 0.008 |
| 204 | 1435282_at | Fam189a2 | family with sequence similarity 189, member A2 | 1.6 | 0.002 |
| 205 | 1447475_at | Fam189a2 | family with sequence similarity 189, member A2 | 1.6 | 0.003 |
| 206 | 1432884_at | Fam23a | family with sequence similarity 23, member A | 1.6 | 0.004 |
| 207 | 1452513_a_at | Fanca | Fanconi anemia, complementation group A | 1.7 | 0.006 |
| 208 | 1449141_at | Fblim1 | filamin binding LIM protein 1 | 1.5 | 0.006 |
| 209 | 1438336_at | Fbxw11 | F-box and WD-40 domain protein 11 | 1.6 | 0.006 |
| 210 | 1426090_a_at | Fert2 | fer (fms/fps related) protein kinase, testis specific 2 | 1.5 | 0.006 |
| 211 | 1426041_a_at | Fgd4 | FYVE, RhoGEF and PH domain containing 4 | 1.4 | 0.009 |
| 212 | 1429310_at | Flrt3 | fibronectin leucine rich transmembrane protein 3 | 1.5 | 0.006 |
| 213 | 1419924_at | Fnip1 | folliculin interacting protein 1 | 1.5 | 0.009 |
| 214 | 1425291_at | Foxj1 | forkhead box J1 | 2.0 | 0.008 |
| 215 | 1456367_at | Fut8 | fucosyltransferase 8 | 1.7 | 0.007 |
| 216 | 1434788_at | Fzd3 | frizzled homolog 3 (Drosophila) | 1.5 | 0.007 |
| 217 | 1449730_s_at | Fzd3 | frizzled homolog 3 (Drosophila) | 1.4 | 0.007 |
| 218 | 1441911_x_at | Gart | phosphoribosylglycinamide formyltransferase | 1.6 | 0.007 |
| 219 | 1449232_at | Gata1 | GATA binding protein 1 | 1.6 | 0.006 |
| 220 | 1418904_at | Gfpt1 | glutamine fructose-6-phosphate transaminase 1 | 1.4 | 0.006 |
| 221 | 1416715_at | Gjb3 | gap junction protein, beta 3 | 1.6 | 0.001 |
| 222 | 1457933_at | Gm1964 | predicted gene 1964 | 1.4 | 0.003 |
| 223 | 1459561_at | Gm3924 | predicted gene 3924 | 1.4 | 0.009 |
| 224 | 1453869_at | Gm5085 | predicted gene 5085 | 1.5 | 0.008 |
| 225 | 1439156_at | Gm962 | predicted gene 962 | 1.5 | 0.005 |
| 226 | 1438888_at | Gmcl1 | germ cell-less homolog 1 (Drosophila) | 1.5 | 0.010 |
| 227 | 1435978_at | Gmppa | GDP-mannose pyrophosphorylase A | 1.4 | 0.009 |
| 228 | 1422817_at | Gp5 | glycoprotein 5 (platelet) | 1.6 | 0.005 |
| 229 | 1448303_at | Gpnmb | glycoprotein (transmembrane) nmb | 1.5 | 0.003 |
| 230 | 1434725_at | Gramd1c | GRAM domain containing 1C | 1.5 | 0.005 |
| 231 | 1435722_at | Gria4 | glutamate receptor, ionotropic, AMPA4 (alpha 4) | 1.6 | 0.005 |
| 232 | 1439286_at | Grik2 | glutamate receptor, ionotropic, kainate 2 (beta 2) | 1.6 | 0.005 |
| 233 | 1434007_at | Gyltl1b | glycosyltransferase-like 1B | 1.8 | 0.005 |
| 234 | 1438858_x_at | H2-Aa | histocompatibility 2, class II antigen A, alpha | 2.1 | 0.006 |
| 235 | 1434572_at | Hdac9 | histone deacetylase 9 | 1.3 | 0.005 |
| 236 | 1456631_at | Heatr7b1 | HEAT repeat containing 7B1 | 1.7 | 0.002 |
| 237 | 1440656_at | Hecw1 | HECT, C2 and WW domain containing E3 ubiquitin protein ligase 1 | 1.7 | 0.001 |
| 238 | 1450049_a_at | Hira | histone cell cycle regulation defective homolog A (S. cerevisiae) | 1.8 | 0.004 |
| 239 | 1443020_at | Hmbox1 | homeobox containing 1 | 1.5 | 0.008 |
| 240 | 1438532_at | Hmcn1 | hemicentin 1 | 1.5 | 0.003 |
| 241 | 1435157_at | Hook3 | hook homolog 3 (Drosophila) | 1.6 | 0.003 |
| 242 | 1452400_a_at | Hoxa11as | HOXA11 antisense RNA (non-protein coding) | 1.5 | 0.009 |
| 243 | 1427362_x_at | Hoxc6 | homeo box C6 | 1.5 | 0.007 |
| 244 | 1431099_at | Hoxd8 | homeo box D8 | 1.7 | 0.006 |
| 245 | 1422919_at | Hrasls | HRAS-like suppressor | 1.6 | 0.005 |
| 246 | 1425786_a_at | Hsf4 | heat shock transcription factor 4 | 1.4 | 0.008 |
| 247 | 1452956_a_at | Ifi27l1 | interferon, alpha-inducible protein 27 like 1 | 1.5 | 0.010 |
| 248 | 1425120_x_at | Ifi27l2b | interferon, alpha-inducible protein 27 like 2B | 1.5 | 0.005 |
| 249 | 1417292_at | Ifi47 | interferon gamma inducible protein 47 | 1.5 | 0.003 |
| 250 | 1421992_a_at | Igfbp4 | insulin-like growth factor binding protein 4 | 1.5 | 0.006 |
| 251 | 1427850_x_at | Igh-VJ558 | immunoglobulin heavy chain (J558 family) | 2.9 | 0.009 |
| 252 | 1425454_a_at | Il12a | interleukin 12a | 1.5 | 0.004 |
| 253 | 1422397_a_at | Il15ra | interleukin 15 receptor, alpha chain | 1.5 | 0.008 |
| 254 | 1426566_s_at | Il17re | interleukin 17 receptor E | 1.4 | 0.007 |
| 255 | 1421843_at | Il1rap | interleukin 1 receptor accessory protein | 1.5 | 0.005 |
| 256 | 1420462_at | Il1rapl2 | interleukin 1 receptor accessory protein-like 2 | 2.1 | 0.003 |
| 257 | 1449864_at | Il4 | interleukin 4 | 1.4 | 0.009 |
| 258 | 1446750_at | Impact | imprinted and ancient | 1.4 | 0.005 |
| 259 | 1460491_at | Invs | inversin | 1.8 | 0.001 |
| 260 | 1427387_a_at | Itgb4 | integrin beta 4 | 1.5 | 0.003 |
| 261 | 1431416_a_at | Jam2 | junction adhesion molecule 2 | 1.5 | 0.009 |
| 262 | 1458161_at | Kcnq1ot1 | KCNQ1 overlapping transcript 1 | 1.7 | 0.002 |
| 263 | 1457482_at | Kdm5b | lysine (K)-specific demethylase 5B | 1.3 | 0.010 |
| 264 | 1415855_at | Kitl | kit ligand | 1.7 | 0.006 |
| 265 | 1425192_at | Klhl25 | kelch-like 25 (Drosophila) | 1.4 | 0.009 |
| 266 | 1425123_at | Klhl36 | kelch-like 36 (Drosophila) | 1.7 | 0.010 |
| 267 | 1420770_at | Klk1b24 | kallikrein 1-related peptidase b24 | 1.9 | 0.007 |
| 268 | 1455888_at | Lingo2 | leucine rich repeat and Ig domain containing 2 | 1.5 | 0.006 |
| 269 | 1416304_at | Litaf | LPS-induced TN factor | 1.7 | 0.006 |
| 270 | 1443907_at | Lnpep | leucyl/cystinyl aminopeptidase | 1.8 | 0.007 |
| 271 | 1438127_at | LOC552906 | hypothetical LOC552906 | 1.5 | 0.003 |
| 272 | 1442320_at | LOC553096 | hypothetical LOC553096 | 1.6 | 0.006 |
| 273 | 1445422_at | LOC621549 | hypothetical protein LOC621549 | 1.6 | 0.001 |
| 274 | 1426110_a_at | Lpar1 | lysophosphatidic acid receptor 1 | 1.5 | 0.007 |
| 275 | 1434761_at | Lrrtm3 | leucine rich repeat transmembrane neuronal 3 | 1.4 | 0.003 |
| 276 | 1453528_at | Lta4h | leukotriene A4 hydrolase | 1.6 | 0.005 |
| 277 | 1449789_x_at | Ly6g6c | lymphocyte antigen 6 complex, locus G6C | 1.5 | 0.008 |
| 278 | 1447684_at | Lzic | leucine zipper and CTNNBIP1 domain containing | 1.6 | 0.001 |
| 279 | 1426648_at | Mapkapk2 | MAP kinase-activated protein kinase 2 | 1.3 | 0.010 |
| 280 | 1459387_at | Mast4 | microtubule associated serine/threonine kinase family member 4 | 1.6 | 0.004 |
| 281 | 1417675_a_at | Mdn1 | midasin homolog (yeast) | 1.5 | 0.003 |
| 282 | 1438538_at | Mecp2 | methyl CpG binding protein 2 | 1.6 | 0.006 |
| 283 | 1440091_at | Meis2 | Meis homeobox 2 | 2.1 | 0.001 |
| 284 | 1458919_at | Mkln1 | muskelin 1, intracellular mediator containing kelch motifs | 1.7 | 0.008 |
| 285 | 1441535_at | Mllt3 | myeloid/lymphoid or mixed-lineage leukemia (trithorax homolog, Drosophila); translocated to, 3 | 1.8 | 0.002 |
| 286 | 1440920_at | Mmp14 | matrix metallopeptidase 14 (membrane-inserted) | 1.6 | 0.004 |
| 287 | 1441185_at | Msi2 | Musashi homolog 2 (Drosophila) | 1.5 | 0.009 |
| 288 | 1449559_at | Msx2 | homeobox, msh-like 2 | 1.5 | 0.009 |
| 289 | 1445151_at | Mtf2 | metal response element binding transcription factor 2 | 1.7 | 0.008 |
| 290 | 1420466_at | Mucl1 | mucin-like 1 | 1.5 | 0.001 |
| 291 | 1454946_at | Mybl2 | myeloblastosis oncogene-like 2 | 1.4 | 0.003 |
| 292 | 1447150_at | Mycbp2 | MYC binding protein 2 | 1.7 | 0.008 |
| 293 | 1459679_s_at | Myo1b | myosin IB | 1.8 | 0.002 |
| 294 | 1446407_at | Mysm1 | myb-like, SWIRM and MPN domains 1 | 1.5 | 0.007 |
| 295 | 1430599_at | Myt1l | myelin transcription factor 1-like | 1.9 | 0.008 |
| 296 | 1457804_at | NA | NA | 3.1 | 0.000 |
| 297 | 1418638_at | NA | NA | 2.7 | 0.000 |
| 298 | 1425174_at | NA | NA | 2.6 | 0.001 |
| 299 | 1440425_at | NA | NA | 2.5 | 0.007 |
| 300 | 1445090_at | NA | NA | 2.5 | 0.000 |
| 301 | 1460097_at | NA | NA | 2.5 | 0.000 |
| 302 | 1442491_at | NA | NA | 2.4 | 0.001 |
| 303 | 1439290_at | NA | NA | 2.3 | 0.000 |
| 304 | 1443237_at | NA | NA | 2.3 | 0.003 |
| 305 | 1444622_at | NA | NA | 2.3 | 0.000 |
| 306 | 1459126_at | NA | NA | 2.3 | 0.001 |
| 307 | 1440630_at | NA | NA | 2.2 | 0.008 |
| 308 | 1443179_at | NA | NA | 2.1 | 0.007 |
| 309 | 1446571_at | NA | NA | 2.1 | 0.000 |
| 310 | 1459675_at | NA | NA | 2.1 | 0.008 |
| 311 | 1439123_at | NA | NA | 2.1 | 0.000 |
| 312 | 1439412_at | NA | NA | 2.1 | 0.001 |
| 313 | 1457973_at | NA | NA | 2.0 | 0.004 |
| 314 | 1446799_at | NA | NA | 2.0 | 0.002 |
| 315 | 1442622_at | NA | NA | 2.0 | 0.001 |
| 316 | 1458230_at | NA | NA | 2.0 | 0.000 |
| 317 | 1460158_at | NA | NA | 2.0 | 0.002 |
| 318 | 1457322_at | NA | NA | 2.0 | 0.010 |
| 319 | 1443751_at | NA | NA | 2.0 | 0.000 |
| 320 | 1447527_at | NA | NA | 2.0 | 0.000 |
| 321 | 1456787_at | NA | NA | 2.0 | 0.008 |
| 322 | 1443238_at | NA | NA | 2.0 | 0.001 |
| 323 | 1458360_at | NA | NA | 1.9 | 0.001 |
| 324 | 1441718_at | NA | NA | 1.9 | 0.000 |
| 325 | 1441629_at | NA | NA | 1.9 | 0.009 |
| 326 | 1440650_at | NA | NA | 1.9 | 0.007 |
| 327 | 1439195_at | NA | NA | 1.9 | 0.002 |
| 328 | 1430477_s_at | NA | NA | 1.9 | 0.003 |
| 329 | 1443161_at | NA | NA | 1.9 | 0.010 |
| 330 | 1420068_at | NA | NA | 1.9 | 0.005 |
| 331 | 1441584_at | NA | NA | 1.9 | 0.005 |
| 332 | 1442715_at | NA | NA | 1.9 | 0.007 |
| 333 | 1439929_at | NA | NA | 1.8 | 0.002 |
| 334 | 1427841_at | NA | NA | 1.8 | 0.002 |
| 335 | 1438072_at | NA | NA | 1.8 | 0.002 |
| 336 | 1446406_at | NA | NA | 1.8 | 0.005 |
| 337 | 1457370_at | NA | NA | 1.8 | 0.000 |
| 338 | 1459238_at | NA | NA | 1.8 | 0.002 |
| 339 | 1440465_at | NA | NA | 1.8 | 0.003 |
| 340 | 1458135_at | NA | NA | 1.8 | 0.001 |
| 341 | 1445779_at | NA | NA | 1.8 | 0.005 |
| 342 | 1439711_at | NA | NA | 1.8 | 0.003 |
| 343 | 1440810_x_at | NA | NA | 1.8 | 0.008 |
| 344 | 1457367_at | NA | NA | 1.8 | 0.009 |
| 345 | 1442924_at | NA | NA | 1.8 | 0.001 |
| 346 | 1443075_at | NA | NA | 1.8 | 0.002 |
| 347 | 1439336_at | NA | NA | 1.8 | 0.008 |
| 348 | 1458556_at | NA | NA | 1.8 | 0.007 |
| 349 | 1446809_at | NA | NA | 1.8 | 0.002 |
| 350 | 1446286_at | NA | NA | 1.8 | 0.004 |
| 351 | 1447060_at | NA | NA | 1.8 | 0.001 |
| 352 | 1443410_at | NA | NA | 1.8 | 0.005 |
| 353 | 1443396_at | NA | NA | 1.8 | 0.001 |
| 354 | 1455977_x_at | NA | NA | 1.8 | 0.000 |
| 355 | 1444126_at | NA | NA | 1.8 | 0.008 |
| 356 | 1443018_at | NA | NA | 1.8 | 0.001 |
| 357 | 1447378_at | NA | NA | 1.8 | 0.002 |
| 358 | 1447546_s_at | NA | NA | 1.8 | 0.006 |
| 359 | 1458018_at | NA | NA | 1.8 | 0.003 |
| 360 | 1459702_at | NA | NA | 1.8 | 0.010 |
| 361 | 1442620_at | NA | NA | 1.8 | 0.005 |
| 362 | 1444869_at | NA | NA | 1.7 | 0.001 |
| 363 | 1431237_at | NA | NA | 1.7 | 0.006 |
| 364 | 1443628_at | NA | NA | 1.7 | 0.004 |
| 365 | 1440728_at | NA | NA | 1.7 | 0.003 |
| 366 | 1442910_at | NA | NA | 1.7 | 0.007 |
| 367 | 1447312_at | NA | NA | 1.7 | 0.003 |
| 368 | 1444345_at | NA | NA | 1.7 | 0.002 |
| 369 | 1459746_at | NA | NA | 1.7 | 0.001 |
| 370 | 1456720_at | NA | NA | 1.7 | 0.006 |
| 371 | 1443270_at | NA | NA | 1.7 | 0.005 |
| 372 | 1447581_at | NA | NA | 1.7 | 0.006 |
| 373 | 1449787_at | NA | NA | 1.7 | 0.009 |
| 374 | 1440682_at | NA | NA | 1.7 | 0.003 |
| 375 | 1445336_at | NA | NA | 1.7 | 0.004 |
| 376 | 1449601_x_at | NA | NA | 1.7 | 0.002 |
| 377 | 1436449_at | NA | NA | 1.7 | 0.003 |
| 378 | 1458586_at | NA | NA | 1.7 | 0.003 |
| 379 | 1440660_at | NA | NA | 1.7 | 0.000 |
| 380 | 1419801_x_at | NA | NA | 1.7 | 0.001 |
| 381 | 1419937_at | NA | NA | 1.7 | 0.005 |
| 382 | 1427572_at | NA | NA | 1.7 | 0.007 |
| 383 | 1421674_at | NA | NA | 1.7 | 0.008 |
| 384 | 1458290_at | NA | NA | 1.7 | 0.007 |
| 385 | 1443389_at | NA | NA | 1.7 | 0.009 |
| 386 | 1447062_at | NA | NA | 1.7 | 0.001 |
| 387 | 1458075_at | NA | NA | 1.7 | 0.000 |
| 388 | 1438891_at | NA | NA | 1.7 | 0.002 |
| 389 | 1443072_at | NA | NA | 1.7 | 0.005 |
| 390 | 1446447_at | NA | NA | 1.7 | 0.002 |
| 391 | 1446515_at | NA | NA | 1.7 | 0.008 |
| 392 | 1442049_at | NA | NA | 1.7 | 0.010 |
| 393 | 1440694_at | NA | NA | 1.7 | 0.006 |
| 394 | 1443606_at | NA | NA | 1.6 | 0.006 |
| 395 | 1459409_at | NA | NA | 1.6 | 0.004 |
| 396 | 1443164_at | NA | NA | 1.6 | 0.007 |
| 397 | 1442886_at | NA | NA | 1.6 | 0.009 |
| 398 | 1440954_at | NA | NA | 1.6 | 0.007 |
| 399 | 1442126_at | NA | NA | 1.6 | 0.006 |
| 400 | 1446223_at | NA | NA | 1.6 | 0.001 |
| 401 | 1444609_at | NA | NA | 1.6 | 0.004 |
| 402 | 1446327_at | NA | NA | 1.6 | 0.004 |
| 403 | 1459278_at | NA | NA | 1.6 | 0.007 |
| 404 | 1457175_at | NA | NA | 1.6 | 0.001 |
| 405 | 1459703_at | NA | NA | 1.6 | 0.004 |
| 406 | 1458309_at | NA | NA | 1.6 | 0.002 |
| 407 | 1443138_at | NA | NA | 1.6 | 0.005 |
| 408 | 1455385_at | NA | NA | 1.6 | 0.008 |
| 409 | 1443166_at | NA | NA | 1.6 | 0.010 |
| 410 | 1458002_at | NA | NA | 1.6 | 0.002 |
| 411 | 1438813_at | NA | NA | 1.6 | 0.001 |
| 412 | 1420062_at | NA | NA | 1.6 | 0.001 |
| 413 | 1441538_at | NA | NA | 1.6 | 0.006 |
| 414 | 1445567_at | NA | NA | 1.6 | 0.005 |
| 415 | 1458885_at | NA | NA | 1.6 | 0.002 |
| 416 | AFFX-r2-Bs-phe-M_at | NA | NA | 1.6 | 0.008 |
| 417 | 1445020_at | NA | NA | 1.6 | 0.004 |
| 418 | 1437219_at | NA | NA | 1.6 | 0.005 |
| 419 | 1456933_at | NA | NA | 1.6 | 0.002 |
| 420 | 1444069_at | NA | NA | 1.6 | 0.010 |
| 421 | 1444735_at | NA | NA | 1.6 | 0.002 |
| 422 | 1444732_at | NA | NA | 1.6 | 0.000 |
| 423 | 1440657_at | NA | NA | 1.6 | 0.004 |
| 424 | 1438543_at | NA | NA | 1.6 | 0.005 |
| 425 | 1444641_at | NA | NA | 1.6 | 0.008 |
| 426 | 1453615_at | NA | NA | 1.6 | 0.005 |
| 427 | 1446488_at | NA | NA | 1.6 | 0.002 |
| 428 | 1457617_at | NA | NA | 1.6 | 0.008 |
| 429 | 1443445_at | NA | NA | 1.6 | 0.002 |
| 430 | 1456688_at | NA | NA | 1.6 | 0.007 |
| 431 | 1442704_at | NA | NA | 1.6 | 0.005 |
| 432 | 1445379_at | NA | NA | 1.6 | 0.003 |
| 433 | 1449652_at | NA | NA | 1.6 | 0.005 |
| 434 | 1438762_at | NA | NA | 1.6 | 0.005 |
| 435 | 1443799_at | NA | NA | 1.6 | 0.001 |
| 436 | 1444365_at | NA | NA | 1.6 | 0.008 |
| 437 | 1446324_at | NA | NA | 1.6 | 0.008 |
| 438 | 1459485_at | NA | NA | 1.6 | 0.002 |
| 439 | 1441203_at | NA | NA | 1.5 | 0.004 |
| 440 | 1440433_at | NA | NA | 1.5 | 0.005 |
| 441 | 1436082_at | NA | NA | 1.5 | 0.007 |
| 442 | 1446104_at | NA | NA | 1.5 | 0.005 |
| 443 | 1459571_at | NA | NA | 1.5 | 0.007 |
| 444 | 1443697_at | NA | NA | 1.5 | 0.010 |
| 445 | 1441498_at | NA | NA | 1.5 | 0.007 |
| 446 | 1436087_at | NA | NA | 1.5 | 0.008 |
| 447 | 1459008_at | NA | NA | 1.5 | 0.009 |
| 448 | 1441392_at | NA | NA | 1.5 | 0.004 |
| 449 | 1458916_at | NA | NA | 1.5 | 0.007 |
| 450 | 1443232_at | NA | NA | 1.5 | 0.004 |
| 451 | 1438393_at | NA | NA | 1.5 | 0.001 |
| 452 | 1441775_at | NA | NA | 1.5 | 0.010 |
| 453 | 1442509_at | NA | NA | 1.5 | 0.006 |
| 454 | 1441351_at | NA | NA | 1.5 | 0.002 |
| 455 | 1441425_at | NA | NA | 1.5 | 0.001 |
| 456 | 1457847_at | NA | NA | 1.5 | 0.005 |
| 457 | 1447096_at | NA | NA | 1.5 | 0.004 |
| 458 | 1447322_at | NA | NA | 1.5 | 0.007 |
| 459 | 1443526_at | NA | NA | 1.5 | 0.002 |
| 460 | 1447144_at | NA | NA | 1.5 | 0.008 |
| 461 | 1449580_s_at | NA | NA | 1.5 | 0.005 |
| 462 | 1446138_at | NA | NA | 1.5 | 0.010 |
| 463 | 1444458_at | NA | NA | 1.5 | 0.005 |
| 464 | 1440511_at | NA | NA | 1.5 | 0.010 |
| 465 | 1447123_at | NA | NA | 1.5 | 0.004 |
| 466 | 1458946_at | NA | NA | 1.5 | 0.003 |
| 467 | 1427600_at | NA | NA | 1.5 | 0.005 |
| 468 | 1435839_at | NA | NA | 1.5 | 0.002 |
| 469 | 1443983_at | NA | NA | 1.5 | 0.005 |
| 470 | 1458328_x_at | NA | NA | 1.5 | 0.010 |
| 471 | 1445869_at | NA | NA | 1.5 | 0.006 |
| 472 | 1440196_at | NA | NA | 1.5 | 0.004 |
| 473 | 1458257_at | NA | NA | 1.5 | 0.005 |
| 474 | 1444772_at | NA | NA | 1.5 | 0.007 |
| 475 | 1445129_at | NA | NA | 1.5 | 0.006 |
| 476 | 1441674_at | NA | NA | 1.5 | 0.009 |
| 477 | 1441701_at | NA | NA | 1.5 | 0.007 |
| 478 | 1441267_at | NA | NA | 1.5 | 0.003 |
| 479 | 1458357_x_at | NA | NA | 1.5 | 0.006 |
| 480 | 1441475_at | NA | NA | 1.5 | 0.004 |
| 481 | 1446504_at | NA | NA | 1.5 | 0.004 |
| 482 | 1427827_at | NA | NA | 1.4 | 0.004 |
| 483 | 1439652_at | NA | NA | 1.4 | 0.006 |
| 484 | 1446412_at | NA | NA | 1.4 | 0.006 |
| 485 | 1441299_at | NA | NA | 1.4 | 0.003 |
| 486 | 1447661_at | NA | NA | 1.4 | 0.009 |
| 487 | 1444971_at | NA | NA | 1.4 | 0.005 |
| 488 | 1457938_at | NA | NA | 1.4 | 0.007 |
| 489 | 1422948_s_at | NA | NA | 1.4 | 0.010 |
| 490 | 1445561_at | NA | NA | 1.4 | 0.010 |
| 491 | 1446422_at | NA | NA | 1.4 | 0.010 |
| 492 | 1459689_at | NA | NA | 1.4 | 0.005 |
| 493 | 1458891_at | NA | NA | 1.4 | 0.006 |
| 494 | 1459371_at | NA | NA | 1.4 | 0.009 |
| 495 | 1447951_at | NA | NA | 1.4 | 0.003 |
| 496 | 1443024_at | NA | NA | 1.4 | 0.008 |
| 497 | 1440533_at | NA | NA | 1.4 | 0.007 |
| 498 | 1441164_at | NA | NA | 1.4 | 0.007 |
| 499 | 1456223_at | NA | NA | 1.4 | 0.007 |
| 500 | 1432638_at | NA | NA | 1.4 | 0.009 |
| 501 | 1458432_at | NA | NA | 1.4 | 0.004 |
| 502 | 1442241_at | NA | NA | 1.4 | 0.009 |
| 503 | 1456928_at | NA | NA | 1.4 | 0.008 |
| 504 | 1456669_at | NA | NA | 1.4 | 0.009 |
| 505 | 1456801_at | NA | NA | 1.4 | 0.006 |
| 506 | 1450822_at | NA | NA | 1.3 | 0.008 |
| 507 | 1441020_at | NA | NA | 1.3 | 0.009 |
| 508 | 1441444_at | Nbeal1 | neurobeachin like 1 | 1.6 | 0.002 |
| 509 | 1448428_at | Nbl1 | neuroblastoma, suppression of tumorigenicity 1 | 1.4 | 0.007 |
| 510 | 1448746_at | Nbn | nibrin | 1.4 | 0.006 |
| 511 | 1439556_at | Ncam1 | neural cell adhesion molecule 1 | 1.7 | 0.004 |
| 512 | 1450976_at | Ndrg1 | N-myc downstream regulated gene 1 | 1.6 | 0.004 |
| 513 | 1444003_at | Neurl3 | neuralized homolog 3 homolog (Drosophila) | 1.4 | 0.009 |
| 514 | 1447549_x_at | Ninj1 | ninjurin 1 | 1.4 | 0.006 |
| 515 | 1434275_at | Nkd2 | naked cuticle 2 homolog (Drosophila) | 1.5 | 0.007 |
| 516 | 1437660_at | Nktr | natural killer tumor recognition sequence | 1.8 | 0.000 |
| 517 | 1422346_at | Nkx2-1 | NK2 homeobox 1 | 1.4 | 0.008 |
| 518 | 1427420_at | Nkx6-2 | NK6 homeobox 2 | 1.6 | 0.007 |
| 519 | 1441447_at | Nol4 | nucleolar protein 4 | 2.0 | 0.001 |
| 520 | 1422142_at | Nphs1 | nephrosis 1 homolog, nephrin (human) | 1.9 | 0.008 |
| 521 | 1428534_at | Nr2c2ap | nuclear receptor 2C2-associated protein | 1.4 | 0.007 |
| 522 | 1421515_at | Nr6a1 | nuclear receptor subfamily 6, group A, member 1 | 1.6 | 0.001 |
| 523 | 1456773_at | Nupl2 | nucleoporin like 2 | 1.4 | 0.007 |
| 524 | 1429753_at | Nxph4 | neurexophilin 4 | 1.8 | 0.005 |
| 525 | 1454424_at | Odz2 | odd Oz/ten-m homolog 2 (Drosophila) | 1.9 | 0.001 |
| 526 | 1422370_at | Olfr49 | olfactory receptor 49 | 1.5 | 0.007 |
| 527 | 1451710_at | Oscar | osteoclast associated receptor | 1.7 | 0.006 |
| 528 | 1423002_at | Pag1 | phosphoprotein associated with glycosphingolipid microdomains 1 | 1.5 | 0.006 |
| 529 | 1446159_at | Pak7 | p21 protein (Cdc42/Rac)-activated kinase 7 | 2.0 | 0.002 |
| 530 | 1444317_at | Pcdh15 | protocadherin 15 | 1.6 | 0.009 |
| 531 | 1450263_at | Pcdhb5 | protocadherin beta 5 | 1.5 | 0.007 |
| 532 | 1427160_at | Pcf11 | cleavage and polyadenylation factor subunit homolog (S. cerevisiae) | 1.4 | 0.008 |
| 533 | 1446272_at | Pctk2 | PCTAIRE-motif protein kinase 2 | 1.7 | 0.002 |
| 534 | 1436966_at | Peli2 | pellino 2 | 1.4 | 0.009 |
| 535 | 1453283_at | Pgm1 | phosphoglucomutase 1 | 1.4 | 0.008 |
| 536 | 1439527_at | Pgr | progesterone receptor | 1.4 | 0.005 |
| 537 | 1441145_at | Phf21a | PHD finger protein 21A | 1.6 | 0.007 |
| 538 | 1454999_at | Phf21b | PHD finger protein 21B | 1.4 | 0.008 |
| 539 | 1438535_at | Phip | pleckstrin homology domain interacting protein | 1.7 | 0.005 |
| 540 | 1457640_x_at | Pigs | phosphatidylinositol glycan anchor biosynthesis, class S | 1.5 | 0.007 |
| 541 | 1451335_at | Plac8 | placenta-specific 8 | 1.6 | 0.001 |
| 542 | 1452517_at | Plekhh1 | pleckstrin homology domain containing, family H (with MyTH4 domain) member 1 | 1.7 | 0.008 |
| 543 | 1418595_at | Plin4 | perilipin 4 | 1.5 | 0.003 |
| 544 | 1450952_at | Pln | phospholamban | 1.4 | 0.005 |
| 545 | 1425371_at | Polb | polymerase (DNA directed), beta | 1.5 | 0.005 |
| 546 | 1427094_at | Pole2 | polymerase (DNA directed), epsilon 2 (p59 subunit) | 1.5 | 0.006 |
| 547 | 1453420_at | Pou5f2 | POU domain class 5, transcription factor 2 | 1.7 | 0.004 |
| 548 | 1435861_at | Ppfia1 | protein tyrosine phosphatase, receptor type, f polypeptide (PTPRF), interacting protein, alpha 1 | 1.7 | 0.007 |
| 549 | 1419681_a_at | Prok2 | prokineticin 2 | 1.8 | 0.005 |
| 550 | 1446560_at | Prss23 | protease, serine, 23 | 1.9 | 0.000 |
| 551 | 1425549_at | Psen1 | presenilin 1 | 1.4 | 0.005 |
| 552 | 1450375_at | Pspn | persephin | 1.6 | 0.007 |
| 553 | 1448816_at | Ptgis | prostaglandin I2 (prostacyclin) synthase | 1.7 | 0.007 |
| 554 | 1449798_at | Rab3c | RAB3C, member RAS oncogene family | 1.8 | 0.000 |
| 555 | 1417620_at | Rac2 | RAS-related C3 botulinum substrate 2 | 1.8 | 0.000 |
| 556 | 1435826_at | Rad18 | RAD18 homolog (S. cerevisiae) | 1.4 | 0.004 |
| 557 | 1442775_at | Rangap1 | RAN GTPase activating protein 1 | 1.4 | 0.004 |
| 558 | 1438828_at | Rapgef6 | Rap guanine nucleotide exchange factor (GEF) 6 | 1.8 | 0.009 |
| 559 | 1429024_at | Rbm20 | RNA binding motif protein 20 | 1.6 | 0.009 |
| 560 | 1443715_at | Rbm24 | RNA binding motif protein 24 | 1.5 | 0.003 |
| 561 | 1444765_at | Rbpms | RNA binding protein gene with multiple splicing | 1.6 | 0.004 |
| 562 | 1456105_at | Rc3h2 | ring finger and CCCH-type zinc finger domains 2 | 1.4 | 0.009 |
| 563 | 1457019_s_at | Rdh14 | retinol dehydrogenase 14 (all-trans and 9-cis) | 1.4 | 0.005 |
| 564 | 1418892_at | Rhoj | ras homolog gene family, member J | 1.6 | 0.001 |
| 565 | 1449914_at | Ribc1 | RIB43A domain with coiled-coils 1 | 1.6 | 0.002 |
| 566 | 1429490_at | Rif1 | Rap1 interacting factor 1 homolog (yeast) | 1.9 | 0.003 |
| 567 | 1429491_s_at | Rif1 | Rap1 interacting factor 1 homolog (yeast) | 1.9 | 0.005 |
| 568 | 1434684_at | Rin3 | Ras and Rab interactor 3 | 1.6 | 0.002 |
| 569 | 1431100_at | Rinl | Ras and Rab interactor-like | 1.4 | 0.006 |
| 570 | 1452361_at | Rnf20 | ring finger protein 20 | 1.5 | 0.003 |
| 571 | 1441162_at | Rock1 | Rho-associated coiled-coil containing protein kinase 1 | 1.5 | 0.007 |
| 572 | 1440310_at | Runx1t1 | runt-related transcription factor 1; translocated to, 1 (cyclin D-related) | 1.8 | 0.003 |
| 573 | 1439161_at | Saps3 | SAPS domain family, member 3 | 1.6 | 0.004 |
| 574 | 1441573_at | Scmh1 | sex comb on midleg homolog 1 | 1.8 | 0.006 |
| 575 | 1436646_at | Scn2a1 | sodium channel, voltage-gated, type II, alpha 1 | 1.7 | 0.004 |
| 576 | 1459269_at | Sdr39u1 | short chain dehydrogenase/reductase family 39U, member 1 | 1.6 | 0.003 |
| 577 | 1441636_at | Sec14l5 | SEC14-like 5 (S. cerevisiae) | 1.4 | 0.008 |
| 578 | 1448415_a_at | Sema3b | sema domain, immunoglobulin domain (Ig), short basic domain, secreted, (semaphorin) 3B | 2.4 | 0.008 |
| 579 | 1425840_a_at | Sema3f | sema domain, immunoglobulin domain (Ig), short basic domain, secreted, (semaphorin) 3F | 1.4 | 0.010 |
| 580 | 1435946_at | Sepsecs | Sep (O-phosphoserine) tRNA:Sec (selenocysteine) tRNA synthase | 1.5 | 0.003 |
| 581 | 1431300_at | Sgip1 | SH3-domain GRB2-like (endophilin) interacting protein 1 | 1.7 | 0.008 |
| 582 | 1426551_at | Sidt1 | SID1 transmembrane family, member 1 | 1.4 | 0.005 |
| 583 | 1449163_at | Sigirr | single immunoglobulin and toll-interleukin 1 receptor (TIR) domain | 1.5 | 0.007 |
| 584 | 1456508_at | Skint10 | selection and upkeep of intraepithelial T cells 10 | 1.7 | 0.004 |
| 585 | 1425570_at | Slamf1 | signaling lymphocytic activation molecule family member 1 | 1.5 | 0.005 |
| 586 | 1420445_at | Slc16a8 | solute carrier family 16 (monocarboxylic acid transporters), member 8 | 2.0 | 0.007 |
| 587 | 1420966_at | Slc25a15 | solute carrier family 25 (mitochondrial carrier ornithine transporter), member 15 | 1.6 | 0.002 |
| 588 | 1445528_at | Slc28a3 | solute carrier family 28 (sodium-coupled nucleoside transporter), member 3 | 1.8 | 0.000 |
| 589 | 1422788_at | Slc43a3 | solute carrier family 43, member 3 | 1.5 | 0.006 |
| 590 | 1451055_at | Slc45a2 | solute carrier family 45, member 2 | 1.7 | 0.001 |
| 591 | 1444242_at | Slco2a1 | solute carrier organic anion transporter family, member 2a1 | 1.5 | 0.009 |
| 592 | 1422771_at | Smad6 | MAD homolog 6 (Drosophila) | 1.9 | 0.000 |
| 593 | 1458479_at | Smc2 | structural maintenance of chromosomes 2 | 1.7 | 0.006 |
| 594 | 1428539_at | Smg1 | SMG1 homolog, phosphatidylinositol 3-kinase-related kinase (C. elegans) | 1.6 | 0.007 |
| 595 | 1426320_at | Snx29 | sorting nexin 29 | 1.8 | 0.006 |
| 596 | 1423077_at | Snx9 | sorting nexin 9 | 1.5 | 0.007 |
| 597 | 1437865_at | Spata13 | spermatogenesis associated 13 | 1.5 | 0.003 |
| 598 | 1421277_at | Spna1 | spectrin alpha 1 | 1.4 | 0.006 |
| 599 | 1441653_at | Srcap | Snf2-related CREBBP activator protein | 2.0 | 0.008 |
| 600 | 1425829_a_at | Steap4 | STEAP family member 4 | 1.5 | 0.005 |
| 601 | 1427291_at | Sycp1 | synaptonemal complex protein 1 | 1.4 | 0.009 |
| 602 | 1431798_a_at | Syde1 | synapse defective 1, Rho GTPase, homolog 1 (C. elegans) | 1.4 | 0.004 |
| 603 | 1421248_at | Syn3 | synapsin III | 1.5 | 0.006 |
| 604 | 1438282_at | Syt1 | synaptotagmin I | 2.0 | 0.002 |
| 605 | 1439090_at | Tbc1d23 | TBC1 domain family, member 23 | 1.6 | 0.002 |
| 606 | 1421909_at | Tcf20 | transcription factor 20 | 1.9 | 0.002 |
| 607 | 1445895_at | Tcrb-V8.2 | T-cell receptor beta, variable 8.2 | 1.6 | 0.007 |
| 608 | 1423224_at | Tctn2 | tectonic family member 2 | 1.6 | 0.001 |
| 609 | 1425169_at | Tessp2 | testis serine protease 2 | 1.9 | 0.001 |
| 610 | 1452978_at | Tmem138 | transmembrane protein 138 | 1.4 | 0.010 |
| 611 | 1441811_x_at | Tmem176a | transmembrane protein 176A | 1.6 | 0.005 |
| 612 | 1456963_at | Tmem214 | transmembrane protein 214 | 1.5 | 0.008 |
| 613 | 1460451_at | Tmem52 | transmembrane protein 52 | 1.6 | 0.009 |
| 614 | 1458106_at | Tmem60 | transmembrane protein 60 | 1.6 | 0.007 |
| 615 | 1458342_at | Tmem90a | transmembrane protein 90a | 1.5 | 0.008 |
| 616 | 1456344_at | Tnc | tenascin C | 1.5 | 0.001 |
| 617 | 1418424_at | Tnfaip6 | tumor necrosis factor alpha induced protein 6 | 1.6 | 0.004 |
| 618 | 1430259_at | Tnfrsf11a | tumor necrosis factor receptor superfamily, member 11a | 1.5 | 0.003 |
| 619 | 1417291_at | Tnfrsf1a | tumor necrosis factor receptor superfamily, member 1a | 1.4 | 0.006 |
| 620 | 1448951_at | Tnfrsf1b | tumor necrosis factor receptor superfamily, member 1b | 1.4 | 0.007 |
| 621 | 1450272_at | Tnfsf8 | tumor necrosis factor (ligand) superfamily, member 8 | 1.4 | 0.009 |
| 622 | 1450813_a_at | Tnni1 | troponin I, skeletal, slow 1 | 1.8 | 0.002 |
| 623 | 1421998_at | Tor3a | torsin family 3, member A | 1.5 | 0.007 |
| 624 | 1439043_at | Tra2a | transformer 2 alpha homolog (Drosophila) | 2.2 | 0.008 |
| 625 | 1425161_a_at | Trabd | TraB domain containing | 1.7 | 0.002 |
| 626 | 1439910_a_at | Tradd | TNFRSF1A-associated via death domain | 1.6 | 0.007 |
| 627 | 1449571_at | Trhr | thyrotropin releasing hormone receptor | 3.3 | 0.001 |
| 628 | 1443989_at | Trim9 | tripartite motif-containing 9 | 1.4 | 0.009 |
| 629 | 1430920_at | Trmt11 | tRNA methyltransferase 11 homolog (S. cerevisiae) | 1.7 | 0.006 |
| 630 | 1442316_x_at | Trp53bp1 | transformation related protein 53 binding protein 1 | 1.6 | 0.003 |
| 631 | 1431355_s_at | Trpm7 | transient receptor potential cation channel, subfamily M, member 7 | 1.7 | 0.000 |
| 632 | 1445727_at | Ube3a | ubiquitin protein ligase E3A | 1.4 | 0.010 |
| 633 | 1418132_a_at | Ubfd1 | ubiquitin family domain containing 1 | 1.5 | 0.009 |
| 634 | 1437022_at | Ubn2 | ubinuclein 2 | 1.6 | 0.005 |
| 635 | 1426105_a_at | Ubxn11 | UBX domain protein 11 | 1.6 | 0.002 |
| 636 | 1450001_a_at | Ush1c | Usher syndrome 1C homolog (human) | 1.7 | 0.003 |
| 637 | 1428193_at | Usp9x | ubiquitin specific peptidase 9, X chromosome | 1.6 | 0.009 |
| 638 | 1438579_at | Utp14b | UTP14, U3 small nucleolar ribonucleoprotein, homolog B (yeast) | 1.6 | 0.002 |
| 639 | 1451314_a_at | Vcam1 | vascular cell adhesion molecule 1 | 1.7 | 0.002 |
| 640 | 1455299_at | Vgll3 | vestigial like 3 (Drosophila) | 3.6 | 0.000 |
| 641 | 1453593_at | Vgll3 | vestigial like 3 (Drosophila) | 2.3 | 0.002 |
| 642 | 1444050_at | Wisp3 | WNT1 inducible signaling pathway protein 3 | 1.4 | 0.006 |
| 643 | 1443924_at | Wnk3 | WNK lysine deficient protein kinase 3 | 1.6 | 0.007 |
| 644 | 1437822_at | Yme1l1 | YME1-like 1 (S. cerevisiae) | 1.8 | 0.005 |
| 645 | 1431781_at | Ypel1 | yippee-like 1 (Drosophila) | 1.6 | 0.009 |
| 646 | 1422570_at | Yy1 | YY1 transcription factor | 1.5 | 0.009 |
| 647 | 1438269_at | Zbtb38 | zinc finger and BTB domain containing 38 | 1.9 | 0.009 |
| 648 | 1425097_a_at | Zfp106 | zinc finger protein 106 | 1.7 | 0.000 |
| 649 | 1438479_at | Zfp213 | zinc finger protein 213 | 1.9 | 0.004 |
| 650 | 1456023_at | Zfp846 | zinc finger protein 846 | 1.4 | 0.003 |
| 651 | 1455290_at | Znrf2 | zinc and ring finger 2 | 1.5 | 0.003 |
